# How common is hyperkalaemia? A systematic review and meta-analysis of the prevalence and incidence of hyperkalaemia reported in observational studies

**DOI:** 10.1093/ckj/sfab243

**Published:** 2021-12-02

**Authors:** Toby Humphrey, Mogamat Razeen Davids, Mogamat-Yazied Chothia, Roberto Pecoits-Filho, Carol Pollock, Glen James

**Affiliations:** Division of Experimental Medicine and Immunotherapeutics, Department of Medicine, University of Cambridge, Cambridge, UK; Department of Medicine, Stellenbosch University, Stellenbosch, South Africa; Division of Nephrology, Tygerberg Hospital, Cape Town, South Africa; Department of Medicine, Stellenbosch University, Stellenbosch, South Africa; Division of Nephrology, Tygerberg Hospital, Cape Town, South Africa; School of Medicine, Pontifícia Universidade Católica do Paraná, Curitiba, Brazil; Arbor Research Collaborative, Ann Arbor, MI, USA; Kolling Institute, Royal North Shore Hospital, University of Sydney, Camperdown, NSW, Australia; BioPharmaceuticals Medical, AstraZeneca, Cambridge, UK

**Keywords:** hyperkalaemia, incidence, meta-analysis, prevalence, systematic literature review

## Abstract

**Background:**

The prevalence and incidence of hyperkalaemia, a potassium abnormality that can potentially have life-threatening consequences, are unclear.

**Methods:**

The objective was to provide the most comprehensive overview of the epidemiology of hyperkalaemia to date within the general population, across different continents, in different healthcare settings and within pre-specified subgroups. Embase and MEDLINE were searched from database inception to 2 February 2021 using the Ovid SP platform. Relevant congress proceedings from 2018 to 2020 were also reviewed for inclusion. There was no language constraint applied. Observational studies from any time period and language reporting prevalence or incidence of hyperkalaemia within both adult and paediatric populations. Four investigators independently screened abstracts and assessed study quality of those meeting the pre-determined inclusion/exclusion criteria. Data extraction was conducted by the lead author with oversight from the senior author and data were pooled using a random-effects model. The measures assessed were the prevalence and incidence of hyperkalaemia. Prevalence was reported as a percentage, whilst incidence was reported as the rate per 100 person years.

**Results:**

In total, 542 articles were included from an initial search of 14 112 articles. Across all adult studies, we report a prevalence of hyperkalaemia (by any definition/threshold) of 6.3% [95% confidence interval (CI): 5.8–6.8%], with an incidence of hyperkalaemia in the adult population of 2.8 (2.3–3.3) cases per 100 person years. Prevalence within the general population was 1.3% (1.0–1.8%), whilst incidence was 0.4 (0.2–0.8) cases per 100 person years. There was a variation by sex with a prevalence of 6.3% (4.9–8.0%) in males and 5.1% (4.0–6.6%) in females. Prevalence also varied according to the definition/threshold of hyperkalaemia used: >5 mmol/L—8.0% (7.2–8.9), ≥5.5 mmol/L—5.9% (3.5–10.0) and ≥6.0 mmol/L—1.0% (0.8–1.4); hyperkalaemia (by any definition/threshold) was highest amongst patients with end-stage kidney disease (21.5%; 18.3–25.3), kidney transplant patients (21.8%; 16.1–29.5) and patients with acute kidney injury (24.3%; 19.3–30.7).

**Conclusions:**

This novel review provides a comprehensive and valuable resource on the prevalence and incidence of hyperkalaemia to better inform clinicians, healthcare providers and health policy makers on the burden of hyperkalaemia across different healthcare settings, patient populations and continents.

## Graphical Abstract

**Graphical Abstract:**
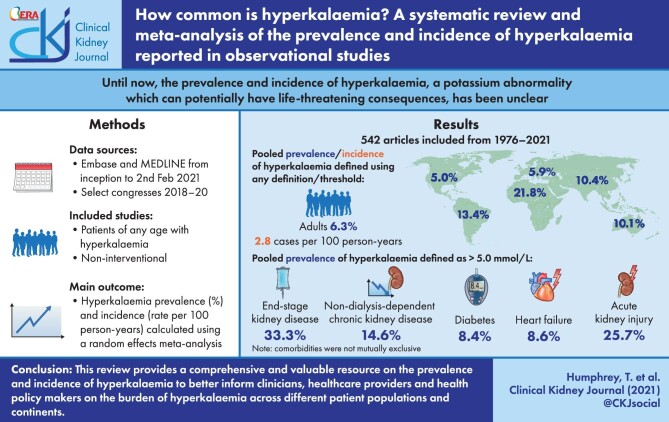

## INTRODUCTION

Hyperkalaemia (HK) is the term used to describe raised serum potassium (sK^+^) concentration within the blood. It is often classified as mild (>5.0–5.9 mmol/L), moderate (6.0–6.4 mmol/L) and severe (≥6.5 mmol/L) [1].

When classifying severity of HK, the rate of change in sK^+^ and the presence of electrocardiogram (ECG) changes are of clinical importance [1] since it is the sK^+^ concentration that determines the resting membrane potential of cells. When sK^+^ is elevated, it can impair muscle function and importantly can cause a reduction in myocardial excitability, with depression of both pacemaking and conducting tissues in the heart leading to life-threatening arrhythmias and sudden cardiac death.

HK most commonly develops in patients with impaired kidney function such as those with acute kidney injury (AKI) or chronic kidney disease (CKD). This can be due to a variety of reasons including (i) increased K^+^ intake from the diet; (ii) alterations in K^+^ homeostasis due to insufficient renal clearance of K^+^ or pharmacological treatments that interfere with renal K^+^ elimination such as renin–angiotensin–aldosterone system inhibitors (RAASi), beta-blockers and K^+^-sparing diuretics such as mineralocorticoid receptor antagonists (MRAs) that are commonly prescribed to patients with CKD, diabetes mellitus and heart failure; and (iii) shift of K^+^ from the intracellular to the extracellular space seen during haemolysis, tissue injury or metabolic acidosis, for example.

The Kidney Disease: Improving Global Outcomes 2021 blood pressure guidelines [2] recommend targeting lower blood pressure targets in patients with CKD, avoiding RAASi discontinuation where possible and encouraging the use of RAASi in broader groups of patients. HK is implicated in limiting the optimal use of these medications [3] that are recommended for use in patients with heart failure, CKD and diabetes [2, 4, 5], and such limitations of optimal therapy are associated with adverse clinical outcomes and increased mortality [6–9]. The publication of the Efficacy and Safety of Finerenone in Subjects With Type 2 Diabetes Mellitus and Diabetic Kidney Disease trial results [10] demonstrated that prescribing of the non-steroidal MRA, finerenone, alongside angiotensin-converting enzyme inhibitor (ACEi)/angiotensin-2-receptor blocker (ARB) therapy lowered risk of CKD progression but resulted in an increase in HK incidence.

Despite the clinical importance of HK, there has remained uncertainty regarding the prevalence and incidence of HK, with many reviews and guidelines acknowledging this when discussing the epidemiology of HK. This is due to the different definitions and thresholds by which studies report HK and the wide range of patient populations described in observational studies. This uncertainty limits awareness of the true burden of disease caused by HK.

As a result of the factors described above, there is a wide variation in the global estimates of the prevalence and incidence of HK. Although studies investigating HK have increased over the last 5 years, there remains no systematic review and meta-analysis of the prevalence and incidence of HK. Therefore, the objective of this systematic literature review (SLR) and meta-analysis is to provide the most comprehensive overview of the epidemiology of HK to date using a range of HK thresholds across different healthcare settings, diseases and continents, to raise awareness and better inform healthcare providers and patients of the burden of HK.

## MATERIALS AND METHODS

This SLR was conducted in accordance with the Preferred Reporting Items for Systematic Reviews and Meta-Analyses (PRISMA) statement with a pre-specified protocol (Supplementary Appendix). The SLR is registered with the International Prospective Register of Systematic Reviews (PROSPERO) (www.crd.york.ac.uk/PROSPERO—ID: CRD4202020631).

A comprehensive, systematic search of the literature was conducted using Embase and MEDLINE, which were searched from database inception to 2 February 2021 (the original search was performed from inception to 31 July 2020 and then updated on 4 February 2021) using the Ovid SP platform. The complete search strategy for MEDLINE and Embase is outlined in the Supplementary Appendix.

In addition, conference proceedings (American Society of Nephrology, European Renal Association–European Dialysis and Transplant Association, International Society of Nephrology, European Society for Diabetes, American Heart Association, European Society of Cardiology, National Kidney Foundation and European Society of Cardiology—Heart Failure) were manually searched for the last 3 years (i.e. 2018–20) to identify relevant abstracts.

The eligibility criteria for the SLR are presented in Table 1.

**Table 1.** Eligibility criteria for the identification of studies reporting HK prevalence or incidence

Category	Inclusion criteria	Exclusion criteria
Population	• Patients with HK	Animal/*in vitro* studies
Study type	• Non-interventional studies, e.g. observational studies and population surveys	
	• Meta-analyses of relevant study designs	Other study types, e.g. randomized controlled trials, case series or reports
Publication type	• Original research studies	
	• Conference abstracts	
	• SLRs of relevant primary publications (these will be considered relevant at the title/abstract review stage and hand-searched for relevant primary studies, but will be excluded during the full-text review stage)	N/A
Outcomes	• Incidence or prevalence or rate or occurrence or frequency of patients with HK	No reporting of relevant epidemiological outcomes
Date limits	• Conference abstracts will be limited to those published in the last 3 years (i.e. in 2018 or later)	N/A
Language	• Any	N/A
Geographic region	• Any	N/A

### Study selection

Four investigators independently screened abstracts for eligibility against pre-determined inclusion/exclusion criteria using the Rayyan online tool (https://rayyan.qcri.org). Following abstract screening, the full text articles were screened by one author and included if eligibility criteria were met.

### Data collection process

Data extraction was undertaken by one investigator, with oversight and quality assurance from the senior author, into a pre-specified data inventory. Relevant information on prevalence and incidence, study characteristics (country, continent and type of study), cohort details, including age of participants, co-morbidities and medications, and HK definition/threshold were extracted.

Quality assessment of included studies was assessed by four investigators using the Joanna Briggs Institute Critical Appraisal Checklist for Prevalence Studies [11] with data collected using a standardized process.

### Summary measures

Studies reporting a specific threshold for defining HK were included in the review and the HK definition/threshold was recorded as such. When studies did not provide an HK definition/threshold, then they were included in the review if they reported the proportion of patients within their study with sK^+^ concentrations at least >5.0 mmol/L. Where studies reported patients with HK but did not provide a definition/threshold for HK, this was recorded as not reported. Both manuscripts and supplemental material (where available) were searched to identify data.

The principal summary measures extracted were the proportion of patients with HK expressed as a percentage (prevalence) and the incidence rate of HK per 100 person years.

Prevalence was calculated using patients with HK as the numerator and total study population as the denominator, multiplied by 100 to give a percentage. Where possible, this method was also used to determine HK prevalence for all subgroups assessed—for example, patients with CKD with HK divided by total patients with CKD for individual studies.

Incidence rate was calculated (if not already reported) by using population number, number of cases of HK and total patient years included in the study and was expressed as a rate per 100 person years. If total patient years were not reported, then the median (or mean) follow-up time was multiplied by the number of patients.

General population was defined as studies reporting patients from an outpatient, registry or primary care setting where the study cohort did not initially specify exact comorbidities nor report patients as taking any specific medications affecting K^+^ homeostasis.

### Synthesis of results

All prevalence and incidence data, respectively, were log transformed and pooled prevalence and pooled incidence rates [with 95% confidence interval (CI)] calculated using a DerSimonian and Laird random effects meta-analysis [12]. Pooled results were calculated for adult, paediatric and neonatal studies separately. Heterogeneity between studies was assessed using the *I^2^* statistic.

To ensure a comprehensive summary of results and to help account for potential small study bias, we descriptively assessed study characteristics and created small and large study categories based on the median cohort size (*N* = 1250) across all studies.

### Risk of bias across studies

Small study bias was assessed using Egger's linear regression test [13, 14], producing funnel plots and 95% CIs. For all statistical tests, P < 0.05 was considered statistically significant.

### Additional analyses

Pooled prevalence and pooled incidence rates were calculated for pre-specified adult subgroups both amongst all studies and also differentiated by HK definition/threshold, study setting and geographical area.

### Statistical analysis

Data management and analysis was conducted using Stata IC 15.1 (StataCorp, College Station, TX, USA).

## RESULTS

**FIGURE 1:**
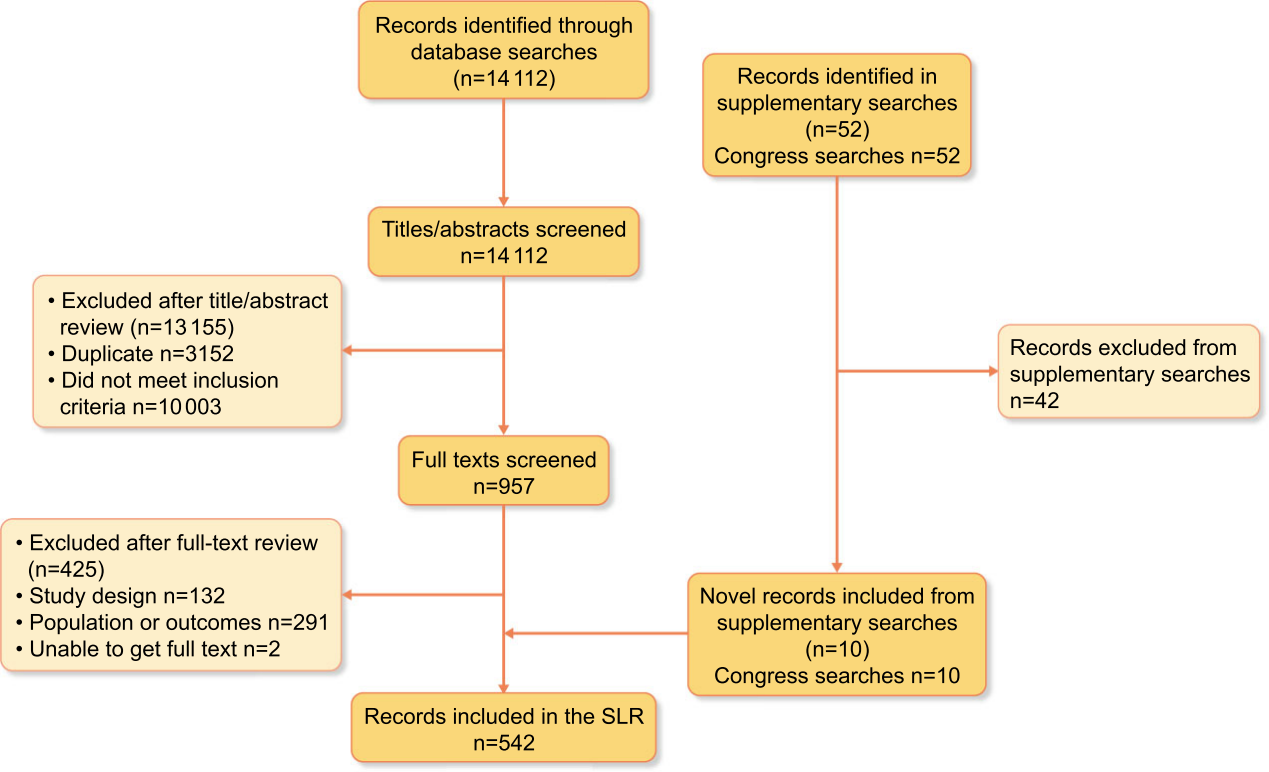
PRISMA flow diagram illustrating the study selection process.

A total of 14 112 abstracts and 52 congress abstracts were identified for review, from which 542 articles were included. The PRISMA flow detailing study selection including reasons for exclusion is found in Figure 1 and a complete list of references for the included studies is found in the Supplementary Appendix.

Papers from any language were included—there were 523 (96.5%) articles published in English. The remaining 3.5% consisted of four papers each in Spanish and German, two papers each in French, Portuguese and Japanese, and one paper each in Chinese, Czech, Danish, Italian and Polish.

In total, there were 13 different definitions/thresholds for HK used amongst the included articles, and studies could include more than one definition/threshold. A breakdown of the number of studies and years of coverage including these definitions/thresholds is provided in Supplementary data, Table S5. The most commonly used definitions/thresholds were sK^+^ measurements of >5.0 mmol/L in 203 studies, >5.5 mmol/L in 241 studies and >6.0 mmol/L in 100 studies.

Included papers spanned the years 1976–2021, with 385 (71.2%) studies published on or after 2012. Pooled prevalence and incidence rate were 8.6% and 4.8 per 100 person years in studies published before 2012 versus 6.2% and 2.6 per 100 person years in studies published on or after 2012. A complete breakdown of pooled prevalence and incidence by decade is provided in Supplementary data, Table S6.

### Overall prevalence of HK

A total of 527 (97.2%) studies reported prevalence data including any population (adult, neonatal and paediatric) from 63 countries between 1976 and 2021 with individual study size ranging from 18 to 32 910 413 patients. Of these 527 studies, 491 (93.1%) were retrospective and 36 (6.9%) were prospective in design. The overall pooled mean prevalence across all studies and definitions/thresholds of HK was 6.6% (95% CI: 6.1–7.1%; Table 2).

**Table 2.** Pooled mean prevalence for all adult studies, healthcare settings, geographical areas and subgroups stratified by HK definition/threshold

	HK by any definition/threshold	>5.0 mmol/L	≥5.5 mmol/L	≥6.0 mmol/L
All adult studies (*n*)	478	193	221	87
Percentage of population affected (95% CI)	6.3 (5.8–6.8)	8.0 (7.2–8.9)	5.9 (3.5–10.0)	1.0 (0.8–1.4)
*I^2^*-test for heterogeneity	100%	100%	100%	99.9%
General population	39	20	15	5
	1.3 (1.0–1.8)	3.8 (3.2–4.4)	1.3 (0.9–1.9)	0.4 (0.2–0.9)
	100%	100%	100%	100%
Sex				
Male	134	68	64	17
	6.3 (4.9–8.0)	9.0 (7.2–11.2)	6.5 (4.5–9.4)	1.6 (0.6–4.1)
	100%	100%	100%	100%
Female	132	67	64	16
	5.1 (4.0–6.6)	7.4 (5.9–9.1)	5.3 (3.8–7.5)	1.4 (0.5–3.6)
	100%	100%	100%	100%
Study type				
Single centre	304	92	139	52
	9.9 (9.1–10.9)	13.3 (11.8–14.9)	11.1 (9.7–12.8)	5.1 (3.6–7.1)
	99.6%	99.5%	99.6%	99.9%
Multi-centre/registry/database	223	106	92	45
	5.1 (4.6–5.6)	8.5 (7.6–9.6)	5.4 (4.5–6.4)	2.0 (1.5–2.5)
	100%	100%	100%	99.9%
Healthcare setting				
Outpatient/primary care	251	110	105	49
	5.0 (4.5–5.5)	8.7 (7.8–9.8)	5.9 (4.9–7.1)	1.7 (1.3–2.3)
	100%	100%	100%	99.9%
Emergency admissions	49	15	18	7
	7.7 (6.1–9.8)	10.5 (8.1–13.7)	10.4 (7.4–14.7)	2.3 (1.5–3.5)
	99.8%	99.7%	99.8%	99.3%
Hospital inpatients	144	40	65	17
	8.7 (7.8–9.7)	12.5 (10.1–15.5)	8.6 (7.4–9.9)	7.5 (5.4–10.5)
	99.9%	99.9%	99.8%	99.5%
Intensive care^a^	28	14	13	4
	7.1 (5.9–8.6)	7.9 (6.5–9.7)	6.6 (4.1–10.6)	6.5 (4.4–9.4)
	99.7%	99.5%	99.5%	98.7%
Dialysis^b^	48	14	22	11
	20.7 (17.4–24.7)	28.4 (22.6–35.6)	21.2 (19.3–23.4)	12.2 (9.8–15.2)
	100%	100%	99.5%	99.6%
Haemodialysis	38	11	18	10
	23.1 (19.1–28.0)	36.2 (28.4–46.2)	23.4 (21.2–25.7)	12.9 (10.3–16.3)
	100%	100%	99.5%	99.6%
Peritoneal dialysis	9	4	4	2
	11.4 (7.7–16.9)	13.8 (8.1–23.4)	12.3 (5.1–29.6)	4.3 (0.5–35.4)
	99.1%	99.4%	98.8%	98.9%
Continent				
Africa	14	5	3	1
	21.8 (14.4–32.9)	12.1 (6.5–22.5)	36.7 (24.0–55.9)	11.5 (6.2–21.3)
	92.6%	88.0%	81.6%	-
Asia	148	41	54	8
	10.4 (9.2–11.7)	11.6 (9.7–13.9)	11.2 (7.9–15.8)	9.4 (4.2–20.8)
	99.9%	100%	99.9%	99.7%
Australasia	9	3	3	3
	10.1 (8.4–12.0)	23.3 (21.0–25.8)	7.3 (5.2–10.4)	4.6 (3.3–6.5)
	99.5%	97.9%	99.1%	99.2%
Europe	175	79	80	39
	5.9 (5.3–6.6)	7.8 (6.8–9.0)	7.3 (6.0–9.0)	2.8 (1.9–4.2)
	100%	100%	99.9%	99.9%
North America	176	59	72	29
	5.0 (4.4–5.8)	9.3 (7.9–11.0)	5.4 (4.4–6.6)	1.6 (1.2–2.3)
	100%	100%	100%	99.9%
South America	14	4	9	5
	13.4 (10.2–17.5)	22.8 (12.3–42.6)	14.9 (10.7–20.9)	6.2 (3.7–10.5)
	96.7%	98.3%	96.0%	86.7%
Global^c^	10	4	5	4
	6.7 (4.1–11.0)	16.5 (3.5–58.0)	8.9 (3.9–20.6)	3.5 (2.7–4.5)
	100%	100%	100%	98.8%
Comorbidity				
CKD non-dialysis^d^	119	57	54	21
	8.5 (7.8–9.3)	14.6 (12.7–16.8)	8.9 (7.6–10.4)	2.5 (1.9–3.3)
	99.9%	99.9%	99.9%	99.7%
End-stage kidney disease^e^	60	17	29	12
	21.5 (18.3–25.3)	33.3 (27.2–40.7)	23.0 (21.0–25.2)	11.6 (9.4–14.3)
	100%	99.9%	99.4%	99.6%
Kidney transplant	18	2	7	4
	21.8 (16.1–29.5)	21.8 (7.0–60.8)	30.8 (20.1–47.2)	12.7 (6.1–26.4)
	98.4%	88.5%	98.2%	92.9%
Diabetes mellitus	64	37	29	11
	5.3 (4.2–6.6)	8.4 (6.3–11.3)	7.2 (4.9–10.8)	1.3 (0.7–2.3)
	99.9%	99.9%	100%	99.5%
Heart failure	104	49	53	23
	6.5 (5.6–7.7)	8.6 (6.7–11.0)	8.0 (6.5–9.8)	3.1 (2.3–4.2)
	99.9%	99.9%	99.7%	99.2%
Hypertension	39	17	13	2
	4.7 (3.9–5.7)	5.1 (3.8–6.8)	3.6 (2.6–4.9)	2.8 (0.5–16.5)
	99.9%	99.9%	99.9%	84.8%
AKI	28	7	11	3
	24.3 (19.3–30.7)	25.7 (16.1–41.2)	31.8 (21.4–47.3)	7.8 (3.5–17.5)
	99.5%	99.7%	98.8%	98.6%
COVID-19 infection	7			
	10.4 (6.8–15.9)			
	74.8%			
Medications				
RAASi^f^	151	53	67	31
	5.8 (5.1–6.6)	9.7 (8.3–11.5)	7.9 (6.6–9.5)	2.5 (1.7–3.7)
	99.9%	99.9%	99.8%	99.7%
ACEi	49	18	25	9
	5.0 (4.0–6.2)	7.9 (5.8–10.8)	7.6 (5.7–10.0)	2.0 (0.8–5.4)
	99.9%	99.9%	99.4%	99.4%
ARB	66	25	27	8
	5.5 (4.1–7.3)	6.7 (4.8–9.3)	8.5 (6.2–11.7)	3.2 (1.1–9.3)
	99.9%	99.9%	99.4%	99.4%
ACEI/ARB plus MRA	9	1	8	2
	14.6 (9.6–22.0)	11.2 (8.7–14.5)	12.8 (7.3–22.2)	21.9 (16.9–28.4)
	95.8%	One study	96.9%	0%
MRA	54	20	22	12
	8.9 (7.2–11.0)	10.1 (7.3–14.1)	11.6 (8.7–15.3)	5.9 (3.9–9.0)
	99.1%	98.6%	96.4%	96.5%
Diuretics	22	13	10	2
	6.6 (5.2–8.3)	8.1 (6.4–10.4)	5.5 (3.0–10.2)	1.3 (0.2–8.2)
	99.5%	99.4%	99.6%	97.3%
CNI	8			
	19.4 (10.8–34.9)			
	97.6%			

a Includes patients admitted to coronary care units and high dependency areas.

b Only includes studies performed in an outpatient dialysis population and includes patients on both haemodialysis and peritoneal dialysis.

c Includes studies performed across different continents.

d Includes patients with pre-dialysis CKD 5 (estimated glomerular filtration rate <15 mL/min/1.73 m_2_.)

e Includes patients from ANY study setting receiving kidney-replacement therapy but NOT pre-dialysis CKD 5 or those with a kidney transplant.

f Includes patients taking ACEi, ARB, renin inhibitors and MRAs.

Empty cells are where no data were available. COVID-19, coronavirus disease 2019.

### Adult prevalence of HK

A total of 478 (88.0%) studies reported prevalence data in adults (age ≥18 years) from 63 countries between 1976 and 2021 with study size ranging from 18 to 32 910 413 patients. HK prevalence ranged from 0.1 to 73.5%. The pooled mean prevalence of HK, by any definition/threshold, in the general population (as defined in the Methods section) was 1.3% (95% CI: 1.0–1.8%; Table 2).

Prevalence in all adult studies combined was 6.3% (95% CI: 5.8–6.8%; Table 2) and there was evidence of small study bias (Egger's test P = 0.008, funnel plot in Supplementary data, Figure S1). There were 220 studies with fewer than 1250 patients with a prevalence of 13.7% (95% CI: 12.6–14.9%), whilst 258 studies with ≥1250 patients had a prevalence of 4.8% (95% CI: 4.4–5.2%). Comprehensive adult prevalence results for all subgroups are stratified by HK definition/threshold and presented in Table 2.

When stratified by sex, the overall prevalence of HK in males (134 studies) was 6.3% (95% CI: 4.9–8.0) and 5.1% (95% CI: 4.0–6.6) in females (132 studies; Table 2). Additional subgroup HK prevalence stratified by sex is presented in Supplementary data, Table S8 where study numbers permitted.

### Paediatric and neonatal prevalence of HK

A total of 26 (4.8%) studies from 19 countries reported prevalence in the general paediatric population between 1987 and 2020. Prevalence ranged from 5.5% (95% CI: 4.1–7.5%) in the outpatient setting to 17.3% (95% CI: 8.3–35.9%) amongst paediatric intensive care patients. The overall pooled mean prevalence of HK in paediatric patients was 14.0% (95% CI: 8.7–22.4%) (Supplementary data, Table S2).

There were 23 (4.2%) studies in the neonatal population from 12 countries between the years 1988 and 2020. All studies were conducted in a neonatal intensive care setting with a pooled mean prevalence of 28.0% (95% CI: 19.7–39.9%) (Supplementary data, Table S2).

### Overall incidence of HK

#### Adult population incidence and subgroups

A total of 65 (12.0%) studies reported incidence data from 16 countries between 1994 and 2020 with study size ranging from 36 to 4 148 468 patients. The pooled mean incidence rate amongst all adult studies was 2.8 (95% CI: 2.3–3.3) cases per 100 person years (Table 2), and there was evidence of small study bias (Egger's test P = 0.05, funnel plot in Supplementary data, Figure S2). Comprehensive adult incidence rate results are stratified by HK definition/threshold in Table 3, and results for all subgroups differentiated by study size are reported in the Supplementary Appendix. There were a lack of data with which to stratify incidence by sex.

**Table 3.** Pooled mean incidence rate for all adult studies, healthcare settings, geographical areas and subgroups stratified by HK definition/threshold

	HK by any definition/threshold	>5.0 mmol/L	≥5.5 mmol/L	≥6.0 mmol/L
All adult studies (*n*)	65	22	32	19
Incidence—cases per 100-person years (95% CI)	2.7 (2.3–3.3)	8.0 (7.2–8.9)	5.9 (3.5–10.0)	1.0 (0.8–1.4)
*I^2^* test for heterogeneity	100%	100%	100%	99.9%
General population	5	3	2	2
	0.3 (0.1–0.7)	1.5 (1.1–2.1)	0.6 (0.4–0.9)	(0.1–0.2)
	100%	99.9%	100%	98.3%
Study type				
Single centre	13	5	9	6
	7.7 (4.8–12.3)	13.9 (10.0–19.2)	20.7 (7.6–56.9)	1.5 (0.4–6.3)
	99.7%	99.0%	99.8%	99.6%
Multi-centre/registry/database	52	17	23	13
	2.2 (1.8–2.7)	7.1 (6.3–8.0)	4.0 (2.2–7.3)	0.9 (0.7–1.2)
	100%	100%	100%	100%
Healthcare setting^a^				
Outpatient/primary care	54	21	26	16
	2.3 (2.1–2.7)	8.7 (7.8–9.7)	3.4 (2.9–4.0)	0.9 (0.7–1.1)
	100%	100%	100%	99.9%
Hospital inpatients	4		3	1
	3.7 (1.4–9.6)		5.1 (1.2–22.3)	0.7 (0.6–0.8)
	99.9%		98.7%	One study
Dialysis^c^	5		3	2
	55.3 (25.1–121.7)		153.8 (84.5–279.9)	222.8 (1.6–106.3)
	100%		100%	99.9%
Continent^b^				
Asia	14		2	1
	(0.8–2.9)		3.8 (0.9–15.9)	0.7 (0.6–0.8)
	99.9%		98.6%	One study
Australasia	1	1		1
	5.8 (4.4–7.6)	7.1 (5.2–9.7)		4.7 (3.1–7.0)
	One study	One study		One study
Europe	25	15	19	8
	4.1 (3.4–4.9)	10.2 (8.8–11.9)	4.1 (3.5–4.9)	1.1 (0.8–1.6)
	100%	100%	99.9%	99.9%
North America	24	5	10	8
	2.3 (1.5–3.6)	6.2 (5.3–7.3)	9.1 (3.7–22.2)	0.7 (0.4–1.2)
	100%	99.9%	100%	99.9%
Global^d^	1		1	1
	9.0 (3.8–21.5)		14.0 (13.8–14.3)	5.8 (5.6–6.0)
	100%		One study	One study
Comorbidity				
CKD non-dialysis^e^	27	13	13	7
	4.2 (3.5–4.9)	8.7 (7.7–9.8)	5.9 (4.7–7.4)	2.5 (1.9–3.3)
	100%	99.9%	99.9%	99.7%
End-stage kidney disease^f^	7	1	4	3
	30.0 (14.5–61.9)	8.3 (7.9–8.6)	104.1 (56.3–193.6)	9.8 (2.4–40.4)
	100%	One study	100%	99.9%
Kidney transplant	3	2	1	2
	4.7 (3.0–7.2)	16.9 (12.0–23.6)	22.0 (15.0–32.4)	0.6 (0.5–0.9)
	99.3%	98.9%	One study	76.5%
Diabetes mellitus	14	7	6	4
	(0.7–1.8)	5.0 (2.5–10.1)	3.5 (1.8–7.0)	0.8 (0.4–1.5)
	100%	100%	99.9%	99.7%
Heart failure	19	10	14	8
	4.3 (3.1–6.0)	13.3 (8.6–20.6)	4.2 (2.9–5.9)	1.4 (0.8–2.5)
	100%	100%	99.9%	99.8%
Hypertension	6	2	3	1
	3.0 (1.8–5.0)	12.1 (3.0–48.7)	2.1 (1.6–2.8)	0.6 (0.5–0.6)
	100%	93.8%	97.9%	One study
Medications				
RAASi^g^	38	10	17	9
	1.7 (1.4–2.1)	7.6 (6.7–8.7)	3.6 (2.3–5.6)	0.9 (0.5–1.7)
	100%	99.9%	99.9%	99.9%
ACEi	12	2	4	3
	0.7 (0.4–1.1)	12.3 (8.0–18.9)	1.3 (0.7–2.3)	0.4 (0.2–1.1)
	99.9%	99.8%	99.5%	99.0%
ARB	20	4	8	4
	(0.8–2.1)	8.1 (5.2–12.8)	3.2 (1.3–8.1)	0.8 (0.2–2.8)
	99.9%	99.8%	99.8%	99.5%
ACEi/ARB	31	9	15	9
	1.3 (1.1–1.8)	7.3 (6.5–8.1)	3.7 (2.3–5.9)	1.0 (0.5–1.8)
	100%	99.8%	99.9%	99.9%
MRA	9	2	4	1
	4.0 (0.2–71.7)	9.9 (2.9–33.6)	4.0 (0.9–18.0)	0.3 (0.2–0.4
	99.6%	99.8%	99.4%	One study
Diuretics	3		3	
	4.0 (0.2–71.7)		4.0 (0.2–71.7)	
	99.9%		99.9%	
CNI	4		4	
	7.1 (0.8–66.2)		7.1 (0.8–66.2)	
	99.7%		99.7%	

a There were no emergency or intensive care studies reporting incidence.

b There were no studies reporting incidence in Africa or South America.

c Only includes studies performed in an outpatient dialysis population. There were no studies reporting incidence of HK in peritoneal dialysis patients, results are specific to patients on haemodialysis.

d Includes studies performed across different continents.

e Includes patients with pre-dialysis CKD 5 (estimated glomerular filtration rate <15 mL/min/1.73 m_2_.)

f Includes patients receiving kidney-replacement therapy from ANY study setting but NOT pre-dialysis CKD 5 or those with a kidney transplant.

g Includes patients taking ACEi, ARB, renin inhibitors and MRAs.

Empty cells are where no data were available.

#### Paediatric and neonatal incidence of HK

Only one single-centre paediatric study of solid organ transplant recipients taking calcineurin inhibitors (CNI) reported an incidence rate of HK of 22.0 (95% CI: 15.0–32.4) cases per 100 person years. There were no neonatal studies that reported incidence data.

#### Additional results

Forest plots of prevalence by K^+^ thresholds (Supplementary data, Figure S3) and further results detailing prevalence and incidence stratified by study size (Supplementary data, Tables S2 and S3), decade of study publication (Supplementary data, Table S6) and study setting plus comorbidities (Supplementary data, Table S7) can be found in the Supplementary data.

## DISCUSSION

This study provides the first and most comprehensive overview of the epidemiology of HK to date using a range of HK thresholds across different healthcare settings, diseases and continents. In the general and adult population, we report prevalence of HK of 2.3% (95% CI: 1.9–2.8%) and 6.3% (95% CI: 5.8–6.8%), respectively, with an incidence of HK in the adult population of 2.8 (95% CI: 2.3–3.3) cases per 100 person years. We observed that in recent years, there has been a notable increase in the number of studies reporting prevalence and incidence of HK with 385 (71.2%) studies published on or after 2012, although no substantial change in prevalence or incidence, respectively, was reported (8.6% and 4.8 per 100 person years in studies published before 2012 versus 6.2% and 2.6 per 100 person years in studies published on or after 2012).

When evaluating HK by different diseases, the prevalence of HK (by any definition/threshold) was >20% amongst patients receiving dialysis, those with a kidney transplant and patients with AKI, compared with <3% in the general population (Table 2). Prevalence increases to >30% in patients with AKI or a kidney transplant when an sK^+^ threshold of ≥5.5 mmol/L is used (Table 2). This is likely to reflect differences in study population, setting and HK definition/threshold used but is also expected as patients with kidney transplants typically have abnormal kidney function. Kidney transplant recipients also often take medications that pre-dispose patients to HK such as CNI for immunosuppression, co-trimoxazole for pneumocystis prophylaxis, and RAASi for blood pressure control and reducing proteinuria. Those with AKI suffer an abrupt loss of kidney function that does not allow for physiological adaptations to control whole body K^+^; metabolic acidosis can also occur, which contributes to HK through movement of K^+^ from the intra- to extra-cellular space. The incidence of HK in these populations followed a similar trend.

For specific classes of medications, we report an increased prevalence of HK in users of RAASi compared to the general population (5.8% versus 2.3%) with no difference between users of ACEi or ARB (5.0% versus 5.5%) (Table 2). Users of MRAs had a higher prevalence of HK at 8.9% (95% CI: 7.2–11.0%), whilst users of dual ACEi/ARB and MRA therapy had a still higher prevalence of 14.6 (9.6–22.0) (Table 2). Patients taking CNIs had the highest HK prevalence [19.4% (95% CI: 10.8–34.9%)] amongst the medications assessed in this review (Table 2). This is likely related to both the patient groups who take CNIs, such as those with kidney transplants, and also to direct class effects of CNIs, which can lower glomerular filtration rate, impair renin release and directly interfere with secretion of K^+^ in the kidneys collecting duct [15].

The observed increasing prevalence and incidence from primary care to secondary and tertiary care is not surprising since those in hospital are likely to be sicker and will also undergo increased monitoring of bloods. HK was highest in the dialysis population and those with impaired kidney function. This is primarily due to impaired removal of K^+^ via the kidney but may also reflect increased K^+^ testing that patients with impaired kidney function undergo [1].

The prevalence of HK reported in observational studies across different continents varies, with the highest prevalence reported in African studies [21.8% (95% CI: 14.4–32.9%)] and lowest in studies from North America [5.0% (95% CI: 4.4–5.8%)] (Table 2). It should be noted, however, that whilst 67% of North American studies examined HK amongst outpatients or those in primary care (Supplementary data, Table S4), African studies were smaller, and focussed on patients at increased risk of HK [primarily those with either CKD or AKI in conjunction with infectious diseases such as Ebola, malaria and human immunodeficiency virus (HIV)]. African populations carry the heaviest burden of HIV infection, with 25.7 million people living with HIV in the WHO Africa Region in 2018 (https://www.afro.who.int/health-topics/hivaids). A broad spectrum of kidney diseases is seen in patients with HIV infection and high prevalence of HK might therefore be expected because of HIV-related kidney disease and the widespread use in these patients of medications (such as trimethoprim), which impair renal K^+^ excretion. There are however a paucity of studies from Africa exploring HK in these specific patient populations and additional data would be invaluable to better quantify the burden of HK in Africa. The prevalence of HK in Asian studies was approximately double that in European and North American studies (10.1% versus 5.9% and 5.0%, respectively) (Table 2), though it should be noted that there was a higher proportion of studies of hospital inpatients in Asia (28% versus 13% and 18%, respectively) (Supplementary data, Table S4).

### Strengths

This systematic review offers a comprehensive overview and meta-analysis of the epidemiology of HK from observational studies both within the general population and specific healthcare settings covering all continents, languages, key subgroups of comorbidities and medication classes. Our review has broad inclusivity of different definitions/thresholds of HK and included multiple bibliographical databases and congress proceedings to capture as many studies as possible, reducing the risk of excluding relevant articles. Other strengths include that search terms were not limited to the abstracts, and full text review included supplemental material. There were no language criteria applied for searches or data extraction in order to limit English-language bias. The outcomes extracted are objective and do not require reviewer judgement, which limits bias in study selection and data extraction. The methodology used in this review is consistent with other published analyses of prevalence data [16], follows PRISMA guidelines and is registered on the PROSPERO registry.

### Limitations

Although there are major strengths of this review, there are limitations to acknowledge. The prevalence and incidence data that contribute to the review and meta-analysis include, and are more reflective of, populations at risk of HK, so careful consideration and context are needed when utilizing these results. Additionally, there was heterogeneity in prevalence reporting between studies, with some reporting point prevalence, others period prevalence and in some studies it was not clear which method had been used. Data on blood sampling methods, storage and processing were unavailable and therefore we were unable to comment on the impact this might have, but acknowledge this could contribute to differences in results observed. Given the broad inclusion criteria, there is potential for duplication of underlying populations and double counting of patients due to the non-exclusivity of patient populations. For example, we acknowledge that studies specific to CKD will include patients who also have heart failure, diabetes, etc., and similarly with medications it is not clear in many studies whether multiple drug use occurred. The broad inclusion also includes different healthcare settings, which contribute to varying ranges of prevalence and incidence and may reflect variations in patient management and monitoring. The studies included in this review also have limitations with respect to the design (e.g. cross-sectional or longitudinal) and underlying real-world data, which include study specific inclusion/exclusion criteria applied and the populations from which they derive are subject to confounding/distortion from both controlled and residual factors, e.g. diet, lack data granularity, may under- or overestimate or misclassify patients and may be subject to publication bias. Also, although we split estimates based on median study size, smaller studies are likely to drive up estimates and may also reflect sicker populations. Lastly, in a very small number of studies, contact with authors to identify and retrieve omitted data or to answer potential queries was not possible and we were also unable to access the full text for two articles.

### Conclusions

This novel review provides a comprehensive and valuable resource on the prevalence and incidence of HK for healthcare professionals and health policymakers when considering the burden of HK for both patients and healthcare systems. This highlights the need for awareness of this common complication and for careful management and prescribing of medications that affect K^+^ homeostasis.

## Supplementary Material

sfab243_Supplemental_File
