# Supplementary material for: How common is hyperkalaemia? A systematic review and meta-analysis of the prevalence and incidence of hyperkalaemia reported in observational studies
Source: Clin Kidney J. 2021 Dec 2;15(4):727–37. doi: 10.1093/ckj/sfab243 (PMC8967676; doi:10.1093/ckj/sfab243)
Supplement: sfab243_Supplemental_File [file sfab243_supplemental_file.docx]

**Supplementary Appendix**

**Figures and Tables**

**Figure 1: Funnel plot to assess publication and small study bias amongst prevalence studies**

**Figure 2: Funnel plot to assess publication and small study bias amongst incidence studies**

**Figure 3: Forest plots displaying pooled mean prevalence for all studies by HK definition (left to right : >5.0mmol/L, ≥5.5 mmol/L, ≥ 6.0 mmol/L)**

**­­­­­­**

Table 1. Search terms for MEDLINE and Embase (to be searched simultaneously via the Ovid SP platform)

| **Domain** | **#** | **Search terms** |  |
| --- | --- | --- | --- |
| **Hyperkalaemia** | |  | exp Hyperkalemia/ |
|  |  |  | hyperkal* |
|  |  |  | Hyperpotass* |
|  |  |  | ((Raised or elevated or increased or high) adj (blood or serum or plasma) adj (potassium)) |
|  |  |  | Exp potassium blood level/ |
|  |  |  | or/1-6 |
| **Epidemiology** | |  | epidemiological data/ or epidemiology/ or epidemiologic studies/ or epidemiolog* |
|  |  |  | incidence/ or inciden* |
|  |  |  | prevalence/ or prevalen* |
|  |  |  | Rate.ti,ab. |
|  |  |  | Occurrence.ti,ab. |
|  |  |  | frequency.ti,ab. |
|  |  |  | Exp case control studies/ or case control.tw or (Case control adj (study or studies)).tw. |
|  |  |  | Exp cohort studies/ or (cohort adj (study or studies)).tw.or Cohort analy$.tw. or cohort analysis/ |
|  |  |  | (Follow up adj (study or studies)).tw. |
|  |  |  | (observational adj (study or studies)).tw. |
|  |  |  | Retrospective study/ or restrospective.tw. |
|  |  |  | Longitudinal study/ or Longitudinal.tw. |
|  |  |  | Prospective study/ |
|  |  |  | Cross-sectional studies/ or Cross sectional.tw. or (cross sectional adj (study or studies)).tw. |
|  |  |  | or/7-20 |
| **Exclusion terms** | |  | (conference abstract or conference review).pt. |
|  |  |  | limit 22 to yr="1974-2017" |
|  |  |  | exp animals/ not exp humans/ |
|  |  |  | Or/22-24 |
| **Total** | |  | 6 and 21 |
|  |  |  | 26 not 25 |

**Databases:** Ovid MEDLINE(R) and Epub Ahead of Print, In-Process & Other Non-Indexed Citations, Daily and Version(R) 1946 to Feb 2, 2021, Embase 1974 to 2021 Feb 2

**Table 2: Summary of the pooled mean prevalence for all combined studies, ages, co-morbidities, study settings, medications and continents and also stratified by study size**

|  | **All studies combined** | **Studies with N <1250** | | **Studies with N ≥1250** |
| --- | --- | --- | --- | --- |
| **Overall** | | | | |
| Percentage of population affected (95% confidence intervals)  I^2^ statistic for heterogeneity | 6.6 (6.1-7.1)  I^2^ – 100%  Egger’s test: p = 0.008 | 14.6 (13.5-15.7)  I^2^ – 96.1% | | 4.8 (4.4-5.2)  I^2^ – 100% |
| **Age** | | | | |
| **All Adults** | 6.3 (5.8-6.8)  I^2^ – 100% | 13.7 (12.6-14.9)  I^2^ – 96.3% | 4.8 (4.4-5.2)  I^2^ – 100% | |
| **Adults >65years** | 3.7 (2.7-5.2)  I^2^ – 100% | 10.4 (2.9-37.0)  I^2^ – 98.3% | 3.4 (2.4-4.9)  I^2^ – 100% | |
| **Paediatric** | 14.0 (8.7-22.4)  I^2^ – 96.7 | 15.9 (12.0-20.9)  I^2^ – 88.3% | 4.6 (0.1-55.3)  I^2^ – 99.6% | |
| **Neonatal** | 28.0 (19.7-39.9)  I^2^ – 96.4% | 33.1 (26.4-41.4)  I^2^ – 87.8% | 5.3 (2.3-12.4)  I^2^ – 80.9% | |
| **K+ Severity** | | | | |
| **>5.0 mmol/L** | 9.6 (8.7-10.6)  I^2^ – 100% | 16.0 (14.2-18.1)  I^2^ – 95% | | 8.4 (7.5-9.3)  I^2^ – 100% |
| **>5.5 mmol/L** | 7.6 (6.8-8.6)  I^2^ – 100% | 15.5 (13.7-17.5)  I^2^ – 96.2% | | 5.3 (4.5-6.1)  I^2^ – 100% |
| **>6.0 mmol/L** | 2.9 (2.4-3.5)  I^2^ – 99.9% | 8.3 (6.1-11.2)  I^2^ – 95.9% | | 1.9 (1.5-2.4)  I^2^ – 99.9% |
| **ICD Code Only** | 3.4 (2.3-4.8)  I^2^ – 100% | 8.5 (6.8-10.6)  1 study | | 3.3 (2.3-4.8)  I^2^ – 100% |
| **Co-Morbidity** | | | | |
| **CKD-ND** | 8.5 (7.8-9.3)  I^2^ – 99.9% | 13.6 (11.8-15.6)  I^2^ – 96.0% | | 7.5 (6.7-8.3)  I^2^ – 99.9 |
| >5.0 mmol/L | 14.6 (12.7-16.8)  I­­_­­_^2^ – 99.9% | 26.1 (22.8-29.8)  I^2^ – 90.1% | | 12.7 (10.9-14.9)  I^2^ – 100% |
| >5.5 mmol/L | 8.9 (7.6-10.4)  I­­^2^ – 99.9% | 13.1 (10.6-16.1)  I^2^ – 96.1% | | 7.5 (6.2-9.1)  I^2^ – 99.9% |
| >6.0 mmol/L | 2.5 (1.9 – 3.3)  I^2^ – 99.7% | 2.3 (1.4-3.9)  I^2^ – 82.4% | | 2.5 (1.9-3.4)  I^2^ – 99.8% |
| **ESKD** | 21.5 (18.3-25.3)  I^2^ – 100% | 29.6 (24.5-35.8)  I^2^ – 96.1% | | 16.3 (13.1-20.4)  I^2^ – 100% |
| >5.0 mmol/L | 33.3 (27.2-40.7)  I^2^ -99.9% | 30.6 (21.2-44.3)  I^2^ – 95.5% | | 35.0 (27.1-45.2)  I^2^ – 100% |
| >5.5 mmol/L | 23.0 (21.0-25.2)  I^2^ – 99.4% | 28.2 (21.1-37.8)  I^2^ – 96.1% | | 18.8 (16.8-21.1)  I^2^ – 99.7% |
| >6.0 mmol/L | 11.6 (9.4-14.3)  I^2^ – 99.6% | 19.6 (11.7-32.9)  I^2^ – 94.2% | | 7.8 (6.0-10.1)  I^2^ – 99.7% |
| **Kidney Transplant** | 21.8 (16.1-29.5)  I^2^ – 98.4% | 24.4 (18.8-31.7)  I^2^ – 90.3% | | 11.3 (5.7-22.3)  I^2^ – 99.7% |
| >5.0 mmol/L | 21.8 (7.0-60.8)  I^2^ -88.5% | 21.8 (7.0-67.9)  I^2^ – 88.5% | | No studies |
| >5.5 mmol/L | 30.8 (20.1-47.2)  I^2^ – 98.2% | 34.3 (25.5-46.2)  I^2^ – 89.4% | | 16.0 (15.4-16.7)  1 study |
| >6.0 mmol/L | 12.7 (6.1-26.4)  I^2^ – 92.9% | 16.8 (8.3-34.0)  I^2^ – 74.4% | | 8.0 (7.6-8.5)  1 study |
| **Diabetes** | 5.3 (4.2-6.6)  I^2^ – 99.9% | 15.0 (11.5-19.7)  I^2^ – 90.5% | | 4.2 (3.3-5.4)  I^2^ – 99.9% |
| >5.0 mmol/L | 8.4 (6.3-11.3)  I^2^ – 99.9% | 9.2 (5.5-15.4)  I^2^ – | | 8.3 (5.9-11.7)  I^2^ – 99.9% |
| >5.5 mmol/L | 7.2 (4.9-10.8)  I^2^ – 100% | 24.3 (19.5-30.1)  I^2^ – 74.3% | | 5.0 (3.2-7.8)  I^2^ – 100% |
| >6.0 mmol/L | 1.3 (0.7-2.3)  I^2^ – 99.5% | No studies | | 1.3 (0.7-2.3)  I^2^ – 99.5% |
| **Heart Failure** | 6.5 (5.6-7.7)  I^2^ – 99.9% | 10.0 (8.3-12.1)  I^2^ – 91.5% | | 5.6 (4.6-6.8)  I^2^ – 99.9% |
| >5.0 mmol/L | 8.6 (6.7-11.0)  I^2^ – 99.9% | 13.6 (9.0-20.5)  I^2^ – 92.9% | | 8.0 (6.1-10.5)  I^2^ – 99.9% |
| >5.5 mmol/L | 8.0 (6.5-9.8)  I^2^ – 99.7% | 11.1 (8.6-14.3)  I^2^ – 89.3% | | 6.6 (5.0-8.6)  I^2^ – 99.8% |
| >6.0 mmol/L | 3.1 (2.3-4.2)  I^2^ – 99.2% | 6.1 (4.1-9.1)  I^2^ – 82.4% | | 2.4 (1.7-3.4)  I^2^ – 99.5% |
| **Hypertension** | 4.7 (3.9-5.7)  I^2^ – 99.9% | 9.4 (5.9-14.9)  I^2^ – 92.3% | | 4.2 (3.5-5.2)  I^2^ – 99.9% |
| >5.0 mmol/L | 5.1 (3.8-6.8)  I^2^ – 99.9% | 9.1 (5.7-14.6)  I^2^ – 80.3% | | 4.7 (3.5-6.3)  I^2^ – 99.9% |
| >5.5 mmol/L | 3.6 (2.6-4.9)  I^2^ – 99.9% | 11.5 (6.2-21.2)  I^2^ – 92.3% | | 2.5 (1.8-3.7)  I^2^ – 99.9% |
| >6.0 mmol/L | 2.8 (0.5-16.5)  I^2^ – 84.8% | 1. (0.3-4.0)   1 study | | 6.1 (5.5-6.8)  1 study |
| **AKI** | 24.3 (19.3-30.7)  I^2^ – 99.5% | 32.2 (26.3-39.4)  I^2^ – 91.3% | | 16.9 (11.7-24.2  I^2^ – 99.8% |
| >5.0 mmol/L | 25.7 (16.1 – 41.2)  I^2^ – 99.7% | 33.6 (19.5-58.1)  I^2^ – 83.4% | | 22.1 (12.2-39.9)  I^2^ – 99.8% |
| >5.5 mmol/L | 31.8 (21.4-47.3)  I^2^ – 98.8% | 38.0 (30.5-47.4)  I^2^ – 85.4% | | 13.0 (4.5-35.5)  I^2^ – 99.8% |
| >6.0 mmol/L | 7.8 (3.5-17.5)  I^2^ – 98.6% | No studies | | 7.8 (3.5-17.5)  I^2^ – 98.6% |
| **COVID-19** | 10.4 (6.8-15.9)  I^2^ – 74.8% | 10.4 (6.8-15.9)  I^2^ – 74.8% | | No data |
| **Healthcare Setting** | | | | |
| **Outpatient/Primary Care** | 5.0 (4.5-5.5)  I^2^ – 100% | 12.7 (11.4-14.2)  I^2^ – 95.6% | | 4.0 (3.5-4.4)  I^2^ – 100% |
| >5.0 mmol/L | 8.7 (7.8-9.8)  I^2^ – 100% | 18.2 (15.7-21.0)  I^2^ – 94.9% | | 7.6 (6.7-8.6)  I^2^ – 100% |
| >5.5 mmol/L | 5.9 (4.9-7.1)  I^2^ – 100% | 14.1 (12.0-16.5)  I^2^ – 95.3% | | 4.2 (3.4-5.3)  I^2^ – 100% |
| >6.0 mmol/L | 1.7 (1.3-2.3)  I^2^ – 99.9% | 3.6 (2.4-5.5)  I^2^ – 92.1% | | 1.4 (1.1-2.0)  I^2^ – 99.9% |
| **Emergency Admissions** | 7.7 (6.1-9.6)  I^2^ – 99.8% | 16.0 (12.4-20.7)  I^2^ – 96.7% | | 5.6 (4.3-7.2)  I^2^ – 99.8% |
| >5.0 mmol/L | 10.5 (8.1-13.7)  I^2^ – 99.7% | 10.9 (6.7-17.6)  I^2^ – 95.4% | | 10.4 (7.6-14.2)  I^2^ – 99.8% |
| >5.5 mmol/L | 10.4 (7.4-14.7)  I^2^ – 99.8% | 24.7 (16.9-36.1)  I^2^ – 96.4% | | 6.8 (4.7-9.9)  I^2^ – 99.8% |
| >6.0 mmol/L | 2.3 (1.5-3.5)  I^2^ – 99.3% | 10.2 (5.1-20.4)  I^2^ – 83.8% | | 1.7 (1.1-2.7)  I^2^ – 99.4% |
| **Hospital Inpatients** | 8.7 (7.8-9.7)  I^2^ – 99.9% | 11.7 (9.9-13.9)  I^2^ – 95.1% | | 6.9 (5.9-8.0)  I^2^ – 99.9% |
| >5.0 mmol/L | 12.5 (10.1-15.5)  I^2^ – 99.9% | 13.5 (10.4-17.4)  I^2^ – 91.4% | | 11.8 (8.7-15.9)  I^2^ – 100% |
| >5.5 mmol/L | 8.6 (7.4-9.9)  I^2^ – 99.8% | 11.8 (9.1-15.4)  I^2^ – 95.3% | | 6.9 (5.7-8.3)  I^2^ – 99.9% |
| >6.0 mmol/L | 7.5 (5.4-10.5)  I^2^ – 99.5% | 11.6 (6.8-19.9)  I^2^ – 90.4% | | 5.0 (3.2-8.0)  I^2^ – 99.7% |
| **Intensive Care*** | 7.1 (5.9-8.6)  I^2^ – 99.7% | 15.2 (10.3-22.4)  I^2^ – 96.5% | | 5.5 (4.5-6.8)  I^2^ – 99.7% |
| >5.0 mmol/L | 7.9 (6.5-9.7)  I^2^ – 99.5% | 8.4 (3.7-19.0)  I^2^ – 96.6% | | 7.8 (6.3-9.6)  I^2^ – 99.6% |
| >5.5 mmol/L | 6.6 (4.1-10.6)  I^2^ – 99.5% | 18.3 (11.6-28.8)  I^2^ – 95.6% | | 2.5 (1.4-4.7)  I^2^ – 99.6% |
| >6.0 mmol/L | 6.5 (4.4-9.4)  I^2^ – 98.7% | No studies | | 6.5 (4.4-9.4)  I^2^ – 98.7% |
| **Dialysis^+^** | 20.7 (17.4-24.7)  I^2^ – 100% | 28.4 (22.4-33.6)  I^2^ – 96.0% | | 15.9 (12.4-20.3)  I^2^ – 100% |
| >5.0 mmol/L | 28.4 (22.6 – 35.6)  I^2^ - 100% | 30.6 (21.2-44.3)  I^2^ – 95.5% | | 26.5 (19.4-36.3)  I^2^ – 100% |
| >5.5 mmol/L | 21.2 (19.3-23.4)  I^2^ - 99.5% | 26.2 (18.9-36.4)  I^2^ – 96.0% | | 17.3 (15.3-19.6)  I^2^ – 99.7% |
| >6.0 mmol/L | 12.2 (9.8-15.2)  I^2^ - 99.6% | 19.6 (11.7-32.9)  I^2^ – 94.2% | | 8.0 (6.0-10.6)  I^2^ – 99.8% |
| **Medication Use** | | | | |
| **RAASi^#^** | 5.8 (5.1-6.6)  I^2^ – 99.9% | 11.4 (9.8-13.3)  I^2^ – 94.5% | | 4.4 (3.8-5.1)  I^2^ – 100% |
| >5.0 mmol/L | 9.7 (8.3-11.5)  I^2^ – 99.9% | 18.8 (15.2-23.4)  I^2^ – 93.2% | | 7.8 (6.4-9.4)  I^2^ – 100% |
| >5.5 mmol/L | 7.9 (6.6-9.5)  I^2^ – 99.8% | 11.9 (9.7-14.7)  I^2^ – 93.9% | | 6.2 (4.9-7.8)  I^2^ – 99.9% |
| >6.0 mmol/L | 2.5 (1.7-3.7)  I^2^ – 99.7% | 5.4 (3.3-8.6)  I^2^ – 92.8% | | 1.7 (1.1-2.8)  I^2^ – 99.8% |
| **ACEi** | 5.0 (4.0-6.2)  I^2^ – 99.9% | 12.3 (9.5-15.8)  I^2^ – 94.6% | | 3.4 (2.6-4.4)  I^2^ – 99.9% |
| >5.0 mmol/L | 7.9 (5.8-10.8)  I^2^ – 99.9% | 17.8 (13.0-24.4)  I^2^ – 92.3% | | 5.2 (3.6-7.6)  I^2^ – 99.9% |
| >5.5 mmol/L | 7.6 (5.7-10.0)  I^2^ – 99.4% | 13.7 (9.9-18.9)  I^2^ – 89.7% | | 6.0 (4.3-8.2)  I^2^ – 99.5% |
| >6.0 mmol/L | 2.0 (0.8-5.4)  I^2^ – 99.4% | 6.9 (3.0-16.3)  I^2^ – 95.5% | | 0.6 (0.2-1.8)  I^2^ – 99.4% |
| **ARB** | 5.5 (4.1-7.3)  I^2^ – 99.9% | 11.5 (8.5-15.6)  I^2^ – 96.2% | | 4.1 (2.9-5.7)  I^2^ – 99.9% |
| >5.0 mmol/L | 6.7 (4.8-9.3)  I^2^ – 99.9% | 14.8 (8.7-25.0)  I^2^ – 94.4% | | 5.3 (3.6-7.8)  I^2^ – 99.9% |
| >5.5 mmol/L | 8.5 (6.2-11.7)  I^2^ – 99.4% | 10.8 (6.9-16.9)  I^2^ – 96.3% | | 7.5 (5.1-11.1)  I^2^ – 99.5% |
| >6.0 mmol/L | 3.2 (1.1-9.3)  I^2^ – 99.4% | 14.1 (4.2-47.5)  I^2^ – 96.7% | | 1.2 (0.3-5.0)  I^2^ – 99.6% |
| **ACEi/ARB** | 5.4 (4.7-6.2)  I^2^ – 99.9% | 11.4 (9.6-13.6)  I^2^ – 95.1% | | 4.0 (3.4-4.8)  I^2^ – 100% |
| >5.0 mmol/L | 9.7 (8.0-11.6)  I^2^ – 99.9% | 19.7 (15.9-24.5)  I^2^ – 91.9% | | 7.8 (6.3-10.0)  I^2^ – 100% |
| >5.5 mmol/L | 7.4 (6.1-9.1)  I^2^ – 99.8% | 11.8 (9.2-15.0)  I^2^ – 95.1% | | 5.8 (4.5-7.5)  I^2^ – 99.9% |
| >6.0 mmol/L | 2.0 (1.3-3.3)  I^2^ – 99.8% | 5.0 (2.7-9.4)  I^2^ – 94.5% | | 1.3 (0.7-2.4)  I^2^ – 99.9% |
| **MRA** | 8.9 (7.2-11.0)  I^2^ – 99.1% | 13.2 (10.2-17.1)  I^2^ – 90.6% | | 6.5 (4.8-8.7)  I^2^ – 99.5% |
| >5.0 mmol/L | 10.1 (7.3-14.1)  I^2^ – 98.6% | 15.9 (8.5-29.6)  I^2^ – 95.5% | | 7.6 (5.1-11.3)  I^2^ – 98.9% |
| >5.5 mmol/L | 11.6 (8.7-15.3)  I^2^ – 96.4% | 14.5 (10.6-19.9)  I^2^ – 85.0% | | 9.0 (6.0-13.5)  I^2^ – 98.1% |
| >6.0 mmol/L | 5.9 (3.9-9.0)  I^2^ – 96.5% | 11.3 (6.8-18.9)  I^2^ – 85.4% | | 3.6 (2.0-6.3)  I^2^ – 97.8% |
| **Diuretics** | 6.6 (5.2-8.3)  I^2^ – 99.5% | 18.4 (10.2-33.1)  I^2^ – 95.9% | | 5.7 (4.4-7.3)  I^2^ – 99.5% |
| >5.0 mmol/L | 8.1 (6.4-10.4)  I^2^ – 99.4% | 37.7 (30.2-47.1)  1 study | | 7.6 (5.9-9.7)  I^2^ – 99.4% |
| >5.5 mmol/L | 5.5 (3.0-10.2)  I^2^ – 99.6% | 22.1 (17.0-28.7)  I^2^ – 46.2% | | 3.7 (1.8-7.4)  I^2^ – 99.7% |
| >6.0 mmol/L | 1.3 (0.2-8.2)  I^2^ – 97.3% | No Studies | | 1.3 (0.2-8.2)  I^2^ – 97.3% |
| **CNIs** | 11.7 (6.5-20.8)  I^2^ – 84.9% | 14.8 (11.0-19.8)  I^2^ – 0.0% | | 5.2 (3.9-6.9)  1 study |
| >5.0 mmol/L | 5.2 (3.9-6.9)  1 study | No studies | | 5.2 (3.9-6.9)  1 study |
| >5.5 mmol/L | No studies | No studies | | No studies |
| >6.0 mmol/L | No studies | No studies | | No studies |
| **Continent** | | | | |
| **Africa** | 21.8 (14.4-32.9)  I^2^ – 92.6% | 20.9 (13.5-32.5)  I^2^ – 93.1% | | 35.7 (23.0-55.3)  I^2^ – 1 study |
| >5.0 mmol/L | 12.1 (6.5-22.5)  I^2^ – 88.0% | 12.1 (6.5-22.5)  I^2^ – 88.0% | | No studies |
| >5.5 mmol/L | 36.7 (24.0-55.9)  I^2^ – 81.6% | 36.7 (24.0-56.0)  I^2^ – 81.6% | | No studies |
| >6.0 mmol/L | 11.5 (6.2-21.3)  I^2^ – 0.0% | 11.5 (6.2-21.3)  I^2^ – 0.0% | | No studies |
| **Asia** | 10.4 (9.2-11.7)  I^2^ – 99.9% | 17.0 (14.8-19.6)  I^2^ – 96.2% | | 6.9 (5.9-8.2)  I^2^ – 100% |
| >5.0 mmol/L | 11.6 (9.7-13.9)  I^2^ – 100% | 16.6 (13.6-20.2)  I^2^ – 91.7% | | 7.8 (6.0-10.2)  I^2^ – 100% |
| >5.5 mmol/L | 11.2 (7.9-15.8)  I^2^ – 99.9% | 16.4 (12.9-20.9)  I^2^ – 96.8% | | 8.9 (5.7-13.9)  I^2^ – 99.9% |
| >6.0 mmol/L | 9.4 (4.2-20.8)  I^2^ – 99.7% | 21.1 (11.2-39.6)  I^2^ – 96.6% | | 2.8 (0.8-9.7)  I^2^ – 99.9% |
| **Australasia** | 10.1 (8.4-12.0)  I^2^ – 99.5% | 25.3 (20.4-31.5)  I^2^ – 53.8 | | 8.8 (7.3-10.7)  I^2^ – 99.6% |
| >5.0 mmol/L | 23.3 (21.0-25.8)  I^2^ – 97.9% | 24.7 (15.5-39.3)  I^2^ – 63.4% | | 23.2 (20.8-25.9)  I^2^ – 98.1% |
| >5.5 mmol/L | 7.3 (5.2-10.4)  I^2^ – 99.1% | 43.3 (25.2-74.6)  1 study | | 6.6 (4.6-9.4)  I^2^ – 99.2% |
| >6.0 mmol/L | 4.6 (3.3-6.5)  I^2^ – 99.2% | 33.3 (17.9-62.0)  1 study | | 4.2 (2.9-5.9)  I^2^ – 99.3% |
| **Europe** | 5.9 (5.3-6.6)  I^2^ – 100% | 14.5 (12.7-16.6)  I^2^ – 95.3% | | 4.4 (3.8-5.0)  I^2^ – 100% |
| >5.0 mmol/L | 7.8 (6.8-9.0)  I^2^ – 100% | §3.9 (10.6-18.3)  I^2^ – 96.4% | | 7.1 (6.1-8.2)  I^2^ – 100% |
| >5.5 mmol/L | 7.3 (6.0-9.0)  I^2^ – 99.9% | 16.8 (13.9-20.3)  I^2^ – 94.7% | | 4.5 (3.5-5.8)  I^2^ – 100% |
| >6.0 mmol/L | 2.8 (1.9-4.2)  I^2^ – 99.9% | 8.4 (5.2-13.5)  I^2^ – 95.0% | | 1.9 (1.2-3.0)  I^2^ – 99.9% |
| **North America** | 5.0 (4.4-5.8)  I^2^ – 100% | 11.3 (9.7-13.3)  I^2^ – 96.2% | | 4.1 (3.5-4.7)  I^2^ – 100% |
| >5.0 mmol/L | 9.3 (7.9-11.0)  I^2^ – 100% | 14.1 (11.0-18.0)  I^2^ – 95.8% | | 8.7 (7.3-10.4)  I^2^ – 100% |
| >5.5 mmol/L | 5.4 (4.4-6.6)  I^2^ – 100% | 12.2 (9.4-15.7)  I^2^ – 96.3% | | 4.0 (3.2-5.1)  I^2^ – 100% |
| >6.0 mmol/L | 1.6 (1.2-2.3)  I^2^ – 99.9% | 4.7 (2.4-9.4)  I^2^ – 96.7% | | 1.3 (0.9-1.8)  I^2^ – 99.9% |
| **South America** | 13.4 (10.2-17.5)  I^2^ – 96.7% | 13.9 (10.9-17.9)  I^2^ – 95.6% | | 5.1 (4.3-6.1)  1 study |
| >5.0 mmol/L | 22.8 (12.3-42.6)  I^2^ – 98.3% | 32.9 (25.0-43.3)  I^2^ – 87.8% | | 5.1 (4.3-6.1)  1 study |
| >5.5 mmol/L | 14.9 (10.7-20.9)  I^2^ – 96.0% | 14.9 (10.7-20.9)  I^2^ – 96.0% | | No studies |
| >6.0 mmol/L | 6.2 (3.7-10.5)  I^2^ – 86.7% | 6.2 (3.7-10.5)  I^2^ – 86.7% | | No studies |
| **Global^^^** | 6.7 (4.1-11.0)  I^2^ – 100% | 18.9 (17.3-20.8)  I^2^ – 0.0% | | 6.2 (3.7=10.4)  I^2^ – 100% |
| >5.0 mmol/L | 16.5 (3.5-58.0)  I^2^ – 100% | 18.9 (17.3-20.8)  I^2^ – 0.0% | | 15.1 (2.0-58.6)  I^2^ – 100% |
| >5.5 mmol/L | 8.9 (3.9-20.6)  I^2^ – 100% | No studies | | 8.9 (3.9-20.6)  I^2^ – 100% |
| >6.0 mmol/L | 3.5 (2.7-4.5)  I^2^ – 98.8% | No studies | | 3.5 (2.7-4.5)  I^2^ – 98.8% |

* Includes patients admitted to coronary care units and high dependency areas

+ Includes studies performed in an outpatient dialysis population and includes patients on both haemodialysis and peritoneal dialysis

# Includes patients taking ACE-Inhibitors, Angiotensin 2 receptor blockers, Renin inhibitors and Minerallo-corticoid receptor antagonists

^ Includes studies performed across different continents

**Table 3: Summary of the pooled mean incidence rate for all combined studies, ages, co-morbidities, study settings, medications and continents and also stratified by study size**

|  | **All studies combined** | **Studies with N <1250** | | **Studies with N ≥1250** |
| --- | --- | --- | --- | --- |
| **Overall** | | | | |
| Incidence – cases per 100-person years (95% CI)  I^2^ statistic for heterogeneity | 2.8 (2.3-3.3)  I^2^ – 100%  Egger’s test: p = 0.05 | 13.5 (7.2-25.3)  I^2^ – 99.7% | | 2.3 (1.9-2.7)  I^2^ – 100% |
| **Age** | | | | |
| **All Adults** | 2.7 (2.3-3.3)  I^2^ – 100% | 13.5 (7.2-25.3)  I^2^ – 99.7% | 2.2 (1.9-2.7)  I^2^ – 100% | |
| **Adults >65** | 2.4 (1.2-4.9)  I^2^ – 100% | No studies | 2.4 (1.2-4.9)  I^2^ – 100% | |
| **Paediatric** | 22.0 (15.0-32.4)  1 study | No studies | 22.0 (15.0-32.4)  1 study | |
| **Neonatal** | No studies | No studies | No studies | |
| **K+ Severity** | | | | |
| **>5.0 mmol/L** | 8.0 (7.2-8.9)  I^2^ – 100% | 10.3 (4.8-22.4)  I^2^ – 98.9% | | 7.9 (7.0-8.8)  I^2^ – 100% |
| **>5.5 mmol/L** | 5.9 (3.5-10.0)  I^2^ – 100% | 17.8 (7.9-40.0)  I^2^ – 99.7% | | 4.1 (2.2-7.4)  I^2^ – 100% |
| **>6.0 mmol/L** | 1.0 (0.8-1.4)  I^2^ – 99.9% | 5.7 (0.6-53.5)  I^2^ – 99.5% | | 0.9 (0.7-1.1)  I^2^ – 100% |
| **ICD Code Only** | 1.9 (1.1-3.2)  I^2^ – 100% | No studies | | 1.9 (1.1-3.2)  I^2^ – 100% |
| **Co-Morbidity** | | | | |
| **CKD-ND** | 4.2 (3.5-4.9)  I^2^ – 100% | 11.5 (8.2-16.0)  I^2^ – 96.9% | | 3.5 (2.9-4.2)  I^2^ – 100% |
| >5.0 mmol/L | 8.7 (7.7-9.8)  I^2^ – 99.9% | 18.1 (14.0-23.6)  I^2^ – 20.9% | | 8.2 (7.2-9.2)  I^2^ – 99.9% |
| >5.5 mmol/L | 5.9 (4.7-7.4)  I^2^ – 99.9% | 10.1 (6.7-15.3)  I^2^ – 97.5% | | 3.8 (2.9-5.0)  I^2^ – 99.9% |
| >6.0 mmol/L | 2.5 (1.9-3.3)  I^2^ – 99.7% | 2.3 (1.4-3.9)  I2 – 82.4% | | 2.5 (1.9-3.4)  I^2^ – 99.8% |
| **ESKD** | 30.0 (14.5-61.9  I^2^ – 100% | 190.0 (121.9-296.1)  I^2^ – 97.6% | | 16.2 (7.0-37.6)  I^2^ – 100% |
| >5.0 mmol/L | 8.3 (7.9-8.6)  1 study | No studies | | 8.3 (7.9-8.6)  1 study |
| >5.5 mmol/L | 104.1 (56.3-193.6)  I^2^ – 100% | 242.0 (195.4-299.8)  I^2^ – 87.7% | | 68.3 (32.1-145.4)  I^2^ – 100% |
| >6.0 mmol/L | 9.8 (2.4-40.4)  I^2^ – 99.9% | 90.2 (76.4-106.3)  1 study | | 3.2 (1.0-10.2)  I^2^ – 99.8% |
| **Kidney Transplant** | 4.7 (3.0-7.2)  I^2^ – 99.3% | 13.9 (9.7-20.1)  1 study | | 4.3 (2.7-6.8)  I^2^ – 99.4% |
| >5.0 mmol/L | 16.9 (12.0-23.6)  I^2^ – 98.9% | No studies | | 16.9 (12.0-23.6)  I^2^ – 98.9% |
| >5.5 mmol/L | 22.0 (15.0-32.4)  1 study | No studies | | 22.0 (15.0-32.4)  1 study |
| >6.0 mmol/L | 0.6 (0.5-0.9)  I^2^ – 76.5% | No studies | | 0.6 (0.5-0.9)  I^2^ – 76.5% |
| **Diabetes** | - 1. (0.7-1.8)   I^2^ – 100% | 12.5 (5.2-29.9)  I^2^ – 95.3% | | 0.9 (0.6-1.5)  I^2^ – 100% |
| >5.0 mmol/L | 5.0 (2.5-10.1)  I^2^ – 100% | No studies | | 5.0 (2.5-10.1)  I^2^ – 100% |
| >5.5 mmol/L | 3.5 (1.8-7.0)  I^2^ – 99.9% | 12.5 (5.2-29.9)  I^2^ – 95.3% | | 1.5 (0.7-3.6)  I^2^ – 100% |
| >6.0 mmol/L | 0.8 (0.4-1.5)  I^2^ – 99.7% | No studies | | 0.8 (0.4-1.5)  I^2^ – 99.7% |
| **Heart Failure** | 4.3 (3.1-6.0)  I^2^ – 100% | 2.4 (1.5-3.8)  I^2^ – 92.3% | | 5.0 (3.5-7.2)  I^2^ – 100% |
| >5.0 mmol/L | 13.3 (8.6-20.6)  I^2^ – 100% | 5.8 (4.6-7.3)  1 study | | 14.9 (9.3-23.8)  I^2^ – 100% |
| >5.5 mmol/L | 4.2 (2.9-5.9)  I^2^ – 99.9% | 2.9 (2.1-4.2)  I^2^ – 76.5% | | 5.0 (3.2-7.8)  I^2^ – 99.9% |
| >6.0 mmol/L | 1.4 (0.8-2.5)  I^2^ – 99.8% | 0.7 (0.1-4.6)  I^2^ – 90.7% | | 1.6 (0.8-3.2)  I^2^ – 99.9% |
| **Hypertension** | 3.0 (1.8-5.0)  I^2^ – 100% | 17.6 (6.9-44.9)  I^2^ – 52.4% | | 2.5 (1.4-4.2)  I^2^ – 100% |
| >5.0 mmol/L | 12.1 (3.0-48.7)  I^2^ – 93.8% | 25.8 (12.9-51.6)  1 study | | 6.2 (6.1-6.3)  1 study |
| >5.5 mmol/L | 2.1 (1.6-2.8)  I^2^ – 97.9% | 9.7 (3.1-30.0)  1 study | | 1. (1.5-2.7)   I^2^ – 98.2% |
| >6.0 mmol/L | 0.6 (0.5-0.6)  1 study | No studies | | 0.6 (0.5-0.6)  1 study |
| **AKI** | 18.3 (17.7-18.8)  I^2^ – 0% | No studies | | 18.3 (17.7-18.8)  I^2^ – 0% |
| >5.0 mmol/L | No studies | No studies | | No studies |
| >5.5 mmol/L | No studies | No studies | | No studies |
| >6.0 mmol/L | No studies | No studies | | No studies |
| **COVID-19** | No studies | No studies | | No studies |
| **Healthcare Setting** | | | | |
| **Outpatient/Primary Care** | 2.3 (2.1-2.7)  I^2^ – 100% | 8.5 (5.8-12.7)  I^2^ – 98.7% | | 2.0 (1.8-2.3)  I^2^ – 100% |
| >5.0 mmol/L | 8.7 (7.8-9.7)  I^2^ – 100% | 10.3 (4.8-22.4)  I^2^ – 98.9% | | 8.6 (7.7-9.7)  I^2^ – 100% |
| >5.5 mmol/L | 3.4 (2.9-4.0)  I^2^ – 100% | 10.2 (6.1-16.8)  I^2^ – 98.6% | | 2.5 (2.1-3.0)  I^2^ – 100% |
| >6.0 mmol/L | 0.9 (0.7-1.1)  I^2^ – 99.9% | 2.2 (0.2-20.7)  I^2^ – 98.8% | | 0.8 (0.7-1.1)  I^2^ – 99.9% |
| **Emergency Admission** | No studies | No studies | | No studies |
| **Hospital Inpatient** | 3.7 (1.4-9.6)  I^2^ – 99.9% | 3.3 (2.2-5.0)  1 study | | 3.8 (1.3-10.6)  I^2^ – 99.9% |
| >5.0 mmol/L | No studies | No studies | | No studies |
| >5.5 mmol/L | 5.1 (1.2-22.3)  I^2^ – 98.7% | 3.3 (2.2-5.0)  1 study | | 6.3 (0.5-72.5)  I^2^ – 99.3% |
| >6.0 mmol/L | 0.7 (0.6-0.8)  1 study | No studies | | 0.7 (0.6-0.8)  1 study |
| **Intensive Care*** | No studies | No studies | | No studies |
| **Dialysis^+^** | 55.3 (25.1-121.7)  I^2^ – 100% | 190.0 (121.9-296.1)  I^2^ – 97.6% | | 29.9 (11.3-78.9)  I^2^ – 100% |
| >5.0 mmol/L | No studies | No studies | | No studies |
| >5.5 mmol/L | 153.8 (84.5-279.9)  I^2^ – 100% | 242.0 (195.4-299.8)  I^2^ – 87.7% | | 117.1 (54.8-250.2)  I^2^ – 100% |
| >6.0 mmol/L | 22.8 (1.6-106.3)  I^2^ – 99.9% | 90.2 (76.4-106.3)  1 study | | 5.8 (5.6-6.0)  1 study |
| **Medication Use** | | | | |
| **RAASi^#^** | 1.7 (1.4-2.1)  I^2^ – 100% | 6.8 (1.5-30.5)  I^2^ – 99.6% | | 1.5 (1.2-1.9)  I^2^ – 100% |
| >5.0 mmol/L | 7.6 (6.7-8.7)  I^2^ – 99.9% | 5.8 (4.6-7.3)  1 study | | 7.7 (6.8-8.9)  I^2^ – 99.9% |
| >5.5 mmol/L | 3.6 (2.3-5.6)  I^2^ – 99.9% | 13.1 (2.1-79.8)  I^2^ – 99.6% | | 2.2 (1.4-3.4)  I^2^ – 99.9% |
| >6.0 mmol/L | 0.9 (0.5-1.7)  I^2^ – 99.9% | 0.2 (0.1-0.7)   1. study | | 1. (0.5-1.8)   I^2^ – 99.9% |
| **ACEi** | 0.7 (0.4-1.1)  I^2^ – 99.9% | No studies | | 0.7 (0.4-1.1)  I^2^ – 99.9% |
| >5.0 mmol/L | 12.3 (8.0-18.9)  I^2^ – 99.8% | No studies | | 12.3 (8.0-18.9)  I^2^ – 99.8% |
| >5.5 mmol/L | 1.3 (0.7-2.3)  I^2^ – 99.5% | No studies | | - 1. (0.7-2.3)   I^2^ – 99.5% |
| >6.0 mmol/L | 0.4 (0.2-1.1)  I^2^ – 99.0% | No studies | | 0.4 (0.2-1.1)  I^2^ – 99.0% |
| **ARB** | - 1. (0.8-2.1)   I^2^ – 99.9% | 4.9 (1.8-13.6)  I^2^ – 67.2% | | - 1. (0.8-1.9)   I^2^ – 99.9% |
| >5.0 mmol/L | 8.1 (5.2-12.8)  I^2^ – 99.8% | No studies | | 8.1 (5.2-12.8)  I^2^ – 99.8% |
| >5.5 mmol/L | 3.2 (1.3-8.1)  I^2^ – 99.8% | 4.9 (1.8-13.6)  I^2^ – 67.2% | | 2.7 (0.9-8.0)  I^2^ – 99.9% |
| >6.0 mmol/L | 0.8 (0.2-2.8)  I^2^ – 99.5% | No studies | | 0.8 (0.2-2.8)  I^2^ – 99.5% |
| **ACEi/ARB** | - 1. (1.1-1.8)   I^2^ – 100% | 6.8 (1.5-30.5)  I^2^ – 99.6% | | - 1. (1.0-1.5)   I^2^ – 100% |
| >5.0 mmol/L | 7.3 (6.5-8.1)  I^2^ – 99.8% | 5.8 (4.6-7.3)  1 study | | 7.4 (6.6-8.3)  I^2^ – 99.8% |
| >5.5 mmol/L | 3.7 (2.3-5.9)  I^2^ – 99.9% | 13.1 (2.1-79.8)  I^2^ – 99.6% | | - 1. (1.3-3.3)   I^2^ – 99.9% |
| >6.0 mmol/L | 1.0 (0.5-1.8)  I^2^ – 99.9% | 0.2 (0.1-0.7)  1 study | | 1. (0.5-2.0)   I^2^ – 99.9% |
| **MRA** | 4.0 (0.2-71.7)  I^2^ – 99.6% | 9.7 (3.1-30.0)  1 study | | 5.1 (3.3-7.9)  I^2^ – 99.6% |
| >5.0 mmol/L | 9.9 (2.9-33.6)  I^2^ – 99.8% | No studies | | 9.9 (2.9-33.6)  I^2^ – 99.8% |
| >5.5 mmol/L | 4.0 (0.9-18.0)  I^2^ – 99.4% | 9.7 (3.1-30.0)  1 study | | 2.8 (0.5-17.0)  I^2^ – 99.7% |
| >6.0 mmol/L | 0.3 (0.2-0.4  1 study | No studies | | 0.3 (0.2-0.4)  1 study |
| **Diuretics** | 4.0 (0.2-71.7)  I^2^ – 99.9% | 17.5 (15.0-20.5)  1 study | | 0.9 (0.8-1.1)  1 study |
| >5.0 mmol/L | No studies | No studies | | No studies |
| >5.5 mmol/L | 4.0 (0.2-71.7)  I^2^ – 99.9% | 17.5 (15.0-20.5)  1 study | | 0.9 (0.8-1.1)  1 study |
| >6.0 mmol/L | No studies | No studies | | No studies |
| **CNIs** | 7.1 (0.8-66.2)  I^2^ – 99.7% | No studies | | 7.1 (0.8-66.2)  I^2^ – 99.7% |
| >5.0 mmol/L | No studies | No studies | | No studies |
| >5.5 mmol/L | 7.1 (0.8-66.2)  I^2^ – 99.7% | No studies | | 7.1 (0.8-66.2)  I^2^ – 99.7% |
| >6.0 mmol/L | No studies | No studies | | No studies |
| **Continent** | | | | |
| **Africa** | No studies | No studies | | No studies |
| **Asia** | - 1. (0.8-2.9)   I^2^ – 99.9% | 10.4 (6.0-18.2)  I^2^ – 80.9% | | 1.3 (0.7-2.6)  I^2^ – 100% |
| >5.0 mmol/L | No studies | No studies | | No studies |
| >5.5 mmol/L | 3.8 (0.9-15.9)  I^2^ – 98.6% | 7.9 (5.7-10.9)  1 study | | - 1. (1.6-2.0)   1 study |
| >6.0 mmol/L | 0.7 (0.6-0.8)  1 study | No studies | | 0.7 (0.6-0.8)  1 study |
| **Australasia** | 5.8 (4.4-7.6)  I^2^ – 99.5% | No studies | | 5.8 (4.4-7.6)  I^2^ – 99.5% |
| >5.0 mmol/L | 7.1 (5.2-9.7)  I^2^ – 99.3% | No studies | | 7.1 (5.2-9.7)  I^2^ – 99.3% |
| >5.5 mmol/L | No studies | No studies | | No studies |
| >6.0 mmol/L | 4.7 (3.1-7.0)  I^2^ – 99.2% | No studies | | 4.7 (3.1-7.0)  I^2^ – 99.2% |
| **Europe** | 4.1 (3.4-4.9)  I^2^ – 100% | 5.8 (3.6-9.3)  I^2^ – 98.6% | | 3.8 (3.1-4.6)  I^2^ – 100% |
| >5.0 mmol/L | 10.2 (8.8-11.9)  I^2^ – 100% | 10.1 (3.5-29.2)  I^2^ – 98.5% | | 10.2 (8.7-12.0)  I^2^ – 100% |
| >5.5 mmol/L | 4.1 (3.5-4.9)  I^2^ – 99.9% | 7.7 (4.4-13.5)  I^2^ – 98.5% | | 3.3 (2.7-4.0)  I^2^ – 100% |
| >6.0 mmol/L | 1.1 (0.8-1.6)  I^2^ – 99.9% | 0.7 (0.1-4.6)  I^2^ – 90.7% | | 1.2 (0.8-1.7)  I^2^ – 100% |
| **North America** | 2.3 (1.5-3.6)  I^2^ – 100% | 42.5 (13.2-137.3)  I^2^ – 99.8% | | - 1. (1.0-2.7)   I^2^ – 100% |
| >5.0 mmol/L | 6.2 (5.3-7.3)  I^2^ – 99.9% | 11.1 (2.3-53.8)  I^2^ – 95.1% | | 6.1 (5.2-7.1)  I^2^ – 99.9% |
| >5.5 mmol/L | 9.1 (3.7-22.2)  I^2^ – 100% | 64.6 (17.7-235.9)  I^2^ – 99.7% | | 5.1 (1.8-14.2)  I^2^ – 100% |
| >6.0 mmol/L | 0.7 (0.4-1.2)  I^2^ – 99.9% | 46.7 (12.6-172.3)  I^2^ – 97.9% | | 0.5 (0.3-0.8)  I^2^ – 99.8% |
| **South America** | No studies | No studies | | No studies |
| **Global^^^** | 9.0 (3.8-21.5)  I^2^ – 100% | No studies | | 9.0 (3.8-21.5)  I^2^ – 100% |
| >5.0 mmol/L | No studies | No studies | | No studies |
| >5.5 mmol/L | 14.0 (13.8-14.3)  1 study | No studies | | 14.0 (13.8-14.3)  1 study |
| >6.0 mmol/L | 5.8 (5.6-6.0)  1 study | No studies | | 5.8 (5.6-6.0)  1 study |

* Includes patients admitted to coronary care units and high dependency areas

+ Includes studies performed in an outpatient dialysis population and includes patients on both haemodialysis and peritoneal dialysis

# Includes patients taking ACE-Inhibitors, Angiotensin 2 receptor blockers, Renin inhibitors and Minerallo-corticoid receptor antagonists

^ Includes studies performed across different continents

**Table 4: Breakdown of studies by study type and continent**

|  | **Africa** | **Asia** | **Australasia** | **Europe** | **North America** | **South America** | **Global** |
| --- | --- | --- | --- | --- | --- | --- | --- |
| **Outpatient/ Primary Care** | 3 | 50 | 3 | 92 | 93 | 7 | 5 |
| **Emergency Admissions** | 4 | 15 | 2 | 17 | 8 | 2 | 0 |
| **Hospital Inpatients** | 5 | 45 | 3 | 44 | 44 | 1 | 2 |
| **Intensive Care** | 0 | 7 | 0 | 10 | 10 | 1 | 0 |
| **Dialysis** | 1 | 19 | 0 | 9 | 15 | 4 | 3 |
| **Paediatric** | 4 | 10 | 1 | 6 | 6 | 0 | 0 |
| **Neonatal** | 1 | 12 | 1 | 2 | 7 | 0 | 0 |
| **Total Studies** | **14** | **148** | **9** | **176** | **177** | **14** | **10** |

**Table 5: Breakdown of studies by hyperkalaemia definition including date range of studies using each definition**

| **Definition**  **(mmol/L)** | **>4.5** | **>4.7** | **>4.8** | **>5.0** | **>5.2** | **>5.3** | **>=5.5** | **>=5.8** | **>=6.0** | **>6.5** | **>=6.5** | **>=7.0** | **ICD code** | **NR** |
| --- | --- | --- | --- | --- | --- | --- | --- | --- | --- | --- | --- | --- | --- | --- |
| **Number of studies** | 7 | 1 | 1 | 203 | 5 | 5 | 241 | 1 | 100 | 2 | 23 | 13 | 39 | 36 |
| **Date range of studies** | 2006-2018 | 2018 | 2012 | 1977 -2021 | 2012-2020 | 1997 -2018 | 1976 -2021 | 2006 | 1982 -2021 | 1992 -2003 | 1988 -2020 | 1988 -2019 | 2003 -2020 | 1988 -2020 |

**Table 6: Hyperkalaemia pooled mean prevalence and pooled mean incidence rate by decade of study publication**

| **Decade of study publication** | **1976-1980** | **1981-1990** | **1991-2000** | **2001-2010** | **2011-present** |
| --- | --- | --- | --- | --- | --- |
| **Prevalence** | | | | | |
| Number of studies  Percentage of population affected (95% confidence intervals)  I^2^ statistic for heterogeneity | 4  10.3 (3.0-36.1)  98.1% | 18  6.4 (4.0-10.4)  99.0% | 24  10.9 (7.4-16.2)  98.3% | 97  8.7 (7.5-9.9)  99.9% | 373  6.2 (5.7-6.8)  100% |
| **Incidence** | | | | | |
| Number of studies  Cases per 100-person years (95% CI)  I^2^ statistic for heterogeneity | 0  -  - | 0  -  - | 3  11.5 (4.3-30.5)  97.3% | 5  4.9 (1.2-20.0)  99.9% | 55  2.6 (2.2-3.1)  100% |

**Table 7: Summary of the pooled mean prevalence in different healthcare settings by HK definition, co-morbidity and medications (where number of studies permitted)**

|  | **HK by any definition** | **>5.0 mmol/L** | **>5.5 mmol/L** | **>6.0 mmol/L** |
| --- | --- | --- | --- | --- |
| **Outpatient/Primary Care studies** | | | | |
| Overall (number of studies)  Percentage of population affected (95% confidence intervals)  I^2^ statistic for heterogeneity | 251  5.0 (4.5-5.5)  100% | 110  8.7 (7.8-9.8)  100% | 105  5.9 (4.9-7.1)  100% | 49  1.7 (1.3-2.3)  99.9% |
| CKD-Non Dialysis | 93  8.1 (7.3-9.0)  100% | 45  14.2 (12.1-16.6)  100% | 43  (7.2-10.2)  99.9% | 18  2.2 (1.6-2.9)  99.8% |
| Diabetes Mellitus | 44  4.5 (3.5-5.8)  99.9% | 28  8.3 (5.9-11.6)  100% | 18  6.6 (4.0-10.8)  100% | 9  (0.6-1.7)  99.1% |
| Heart Failure | 73  6.0 (4.9-7.3)  99.9% | 39  8.6 (6.3-11.6)  93.9% | 39  7.7 (6.0-9.9)  99.7% | 19  2.9 (2.0-4.0)  99.4% |
| Patients taking RAASi | 104  4.6 (4.0-5.4)  99.9% | 42  (7.3-10.6)  100% | 47  6.6 (5.2-8.4)  99.9% | 25  (1.2-3.1)  99.8% |
| **Emergency Admissions** | | | | |
| Overall | 49  7.7 (6.1-9.8)  99.8% | 17  10.5 (8.1-13.7)  99.7% | 20  10.4 (7.4-14.7)  99.8% | 8  2.3 (1.5-3.5)  99.3% |
| CKD-Non Dialysis | 4  13.1 (8.6-19.9)  99.6% | 3  22.1 (15.8-31.0)  99.0% | 2  15.8 (7.9-31.4)  99.4% | 1  4.7 (1.9-12.0)  99.1% |
| Diabetes Mellitus | 5  11.0 (6.0-20.3)  99.15 | 3  7.1 (1.8-28.0)  98.4% | 3  19.4 (10.2-37.1)  97.3% | 1  4.7 (4.1-5.5)  - |
| Heart Failure | 4  6.4 (4.4-9.3)  96.4% | 3  6.4 (4.0-10.5)  97.3% | 1  8.0 (6.6-9.8)  - | 2  5.7 (3.1-10.6)  79.1% |
| Acute Kidney Injury | 12  22.4 (13.4-37.4)  98.8% | 4  26.2 (13.2-52.1)  95.9% | 3  30.0 (6.2-68.6)  99.4% | 1  (1.5-3.0)  - |
| Patients taking RAASi | 6  7.0 (4.6-9.8)  99.4% | 3  14.3 (11.5-17.7)  96.4% | 2  4.6 (3.4-6.1)  95.5% | 2  (1.5-2.8)  81.3% |
| **Hospital Inpatients** |  |  |  |  |
| Overall | 144  8.7 (7.8-9.7)  99.9% | 40  12.5 (10.1-15.5)  99.9% | 65  8.6 (7.4-9.9)  99.8% | 17  7.5 (5.4-10.5)  99.5% |
| CKD-Non Dialysis | 9  13.6 (11.3-16.4)  99.6% | 4  23.6 (16.4-34.0)  99.6% | 7  11.0 (6.8-18.0)  99.6% | 1  13.0 (12.0-14.2)  - |
| Diabetes Mellitus | 8  8.5 (6.1-11.8)  99.7% | 3  14.9 (9.4-23.8)  99.1% | 6  9.2 (6.3-13.5)  99.2% | 1  4.8 (4.4-5.2)  - |
| Heart Failure | 20  10.0 (7.6-13.0)  99.4% | 5  16.0 (7.9-32.2)  99.7% | 14  9.4 (6.7-13.1)  94.9% | 3  3.6 (1.3-9.8)  93.4% |
| Acute Kidney Injury | 8  23.6 (16.1-34.6)  99.7% | 1  46.4 (45.0-47.8)  - | 6  28.5 (20.8-39.1)  89.4% | 1  10.6 (9.9-11.3)  - |
| Patients taking RAASi | 21  12.2 (9.4-15.8)  98.7% | 7  20.3 (14.0-29.4)  90.0% | 14  11.9 (10.0-14.2)  95.2% | 3  9.8 (2.4-39.8)  98.1% |

**Table 8: Summary of the pooled mean prevalence for all adult studies, co-morbidities, study settings, medications and continents stratified by sex (where study numbers permit)**

|  | **Male** | **Female** |
| --- | --- | --- |
| **Overall** | | |
| **General Population** | 1.5 (0.7-3.1)  I^2^ – 100% | 1.2 (0.6-2.5)  I^2^ – 100% |
| **K^+^ Severity** | | |
| **ICD Code Only** | 1.9 (0.3-12.3)  I^2^ – 100% | 1.2 (0.2-6.7)  I^2^ – 100% |
| **Co-Morbidities** | | |
| **CKD-ND**§ | 10.6 (7.7-14.7)  I^2^ – 100% | 7.9 (5.6-11.2)  I^2^ – 100% |
| >5.0 mmol/L | 15.8 (10.4-24.1)  I^2^ – 100% | 11.6 (7.6-17.8)  I^2^ – 100% |
| >5.5 mmol/L | 10.2 (4.2-24.5)  I^2^ – 100% | 7.1 (3.2-16.1)  I^2^ – 100% |
| >6.0 mmol/L | 0.9 (0.4-2.1)  I^2^ – 99.1% | 0.6 (0.4-1.0)  I^2^ – 99.6% |
| **ESKD**\|\| | 20.7 (16.6-28.3)  I^2^ – 99.7% | 16.9 (14.8-25.5)  I^2^ – 99.7% |
| >5.0 mmol/L | 20.9 (14.9-29.4)  I^2^ – 99.6% | 17.0 (10.8-26.9)  I^2^ – 99.8% |
| >5.5 mmol/L | 18.2 (9.7-35.5)  I^2^ – 99.3% | 16.8 (9.2-34.2)  I^2^ – 99.2% |
| >6.0 mmol/L | 7.7 (7.5-7.9)  1 study | 8.3 (8.1-8.5)  1 study |
| **Heart Failure** | 7.3 (5.6-9.5)  I^2^ – 99.9% | 6.9 (5.2-9.0)  I^2^ – 99.9% |
| >5.0 mmol/L | 11.9 (8.8-16.2)  I^2^ – 99.9% | 10.6 (7.7-14.5)  I^2^ – 99.9% |
| >5.5 mmol/L | 4.9 (2.9-8.4)  I^2^ – 99.7% | 4.5 (2.6-7.9)  I^2^ – 99.7% |
| >6.0 mmol/L | 1.2 (0.3-5.4)  I^2^ – 98.9% | 0.6 (0.1-3.3)  I^2^ – 99.1% |
| **Hypertension** | 5.6 (2.2-14.5)  I^2^ – 100% | 3.4 (1.3-8.9)  I^2^ – 100% |
| **AKI** | 22.9 (17.9-29.2)  I^2^ – 72.8% | 22.0 (19.0-25.5)  I^2^ – 41.1% |
| **Healthcare Setting** | | |
| **Outpatient/Primary Care** | 4.9 (3.3-7.1)  I^2^ – 100% | 3.8 (2.6-5.7)  I^2^ – 100% |
| >5.0 mmol/L | 8.5 (6.3-11.3)  I^2^ – 100% | 7.0 (5.2-9.3)  I^2^ – 100% |
| >5.5 mmol/L | 5.4 (2.7-10.5)  I^2^ – 100% | 4.0 (2.1-7.5)  I^2^ – 100% |
| >6.0 mmol/L | 0.5 (0.2-1.2)  I^2^ –99.9% | 0.3 (0.2-0.7)  I^2^ –99.9% |
| **Emergency Admissions** | 5.7 (3.5-9.5)  I^2^ – 99.9% | 4.9 (2.8-8.3)  I^2^ – 99.9% |
| >5.0 mmol/L | 5.8 (3.3-10.4)  I^2^ – 99.8% | 5.0 (3.0-8.5)  I^2^ – 99.8% |
| >5.5 mmol/L | 8.2 (2.3-29.0)  I^2^ – 99.9% | 6.9 (1.7-28.2)  I^2^ – 99.9% |
| >6.0 mmol/L | 3.0 (0.6-15.5)  I^2^ – 99.7% | 2.5 (0.4-16.8)  I^2^ – 99.7% |
| **Hospital Inpatients** | 7.7 (5.9-10.1)  I^2^ – 99.9% | 6.6 (5.0-8.9)  I^2^ – 99.9% |
| >5.0 mmol/L | 12.6 (7.5-21.2)  I^2^ – 99.9% | 8.0 (3.6-17.9)  I^2^ – 100% |
| >5.5 mmol/L | 6.0 (4.0-8.8)  I^2^ – 99.9% | 5.5 (3.9-7.8)  I^2^ – 99.9% |
| >6.0 mmol/L | 5.2 (2.4-11.2)  I^2^ – 99.4% | 5.2 (1.5-17.9)  I^2^ –99.8% |
| **Intensive Care*** | 5.8 (4.0-8.6)  I^2^ – 99.9% | 5.3 (3.4-8.3)  I^2^ – 99.9% |
| **Dialysis^+^** | 20.2 (15.4-26.6)  I^2^ – 99.7% | 18.5 (14.0-24.3)  I^2^ – 99.7% |
| >5.0 mmol/L | 20.9 (14.9-29.4)  I^2^ – 99.6% | 17.0 (10.8-26.9)  I^2^ – 99.8% |
| >5.5 mmol/L | 18.2 (9.7-35.5)  I^2^ – 99.3% | 16.8 (9.2-34.2)  I^2^ – 99.2% |
| >6.0 mmol/L | 7.7 (7.5-7.9)  1 study | 8.3 (8.1-8.5)  1 study |
| **Medication Use** | | |
| **RAASi^#^** | 9.4 (5.4-16.3)  I^2^ – 100% | 8.2 (4.5-15.0)  I^2^ – 100% |
| >5.0 mmol/L | 21.6 (13.3-35.1)  I^2^ – 100% | 17.7 (10.9-28.5)  I^2^ – 100% |
| >5.5 mmol/L | 7.8 (4.7-13.0)  I^2^ – 99.2% | 6.6 (4.1-10.7)  I^2^ – 98.9% |
| **ACEi** | 7.7 (4.4-13.4)  I^2^ – 98.0% | 5.9 (3.3-10.5)  I^2^ – 98.2% |
| **ARB** | 8.1 (1.5-33.8)  I^2^ – 100% | 8.3 (1.3-45.2)  I^2^ – 100% |
| **MRA** | 13.2 (7.0-24.8)  I^2^ – 97.3% | 12.6 (3.6-43.7)  I^2^ – 98.4% |
| **Diuretics** | 5.7 (3.1-10.4)  I^2^ – 99.6% | 5.8 (2.9-11.6)  I^2^ – 99.7% |
| **CNIs** | 7.1 (6.7-7.5)  1 study | 6.7 (6.3-7.1)  1 study |
| **Continent** | | |
| **Africa** | 28.9 (18.4-45.3)  I^2^ – 44.1% | 19.8 (8.8-44.3)  I^2^ – 74.1% |
| **Asia** | 15.1 (10.5-21.8)  I^2^ – 99.3% | 14.6 (10.1-21.1)  I^2^ – 99.3% |
| **Australasia** | 8.7 (2.9-25.7)  I^2^ – 99.9% | 5.7 (1.8-17.9)  I^2^ – 99.9% |
| **Europe** | 5.7 (4.3-7.7)  I^2^ – 100% | 5.1 (3.9-6.6)  I^2^ – 100% |
| >5.0 mmol/L | 7.6 (5.5-10.5)  I^2^ – 100% | 6.7 (5.0-9.0)  I^2^ – 100% |
| >5.5 mmol/L | 6.5 (3.3-12.8)  I^2^ – 100% | 5.7 (3.0-10.9)  I^2^ – 100% |
| >6.0 mmol/L | 0.9 (0.3-2.9)  I^2^ – 99.9% | 0.8 (0.3-2.4)  I^2^ – 99.9% |
| **North America** | 4.5 (2.8-7.3)  I^2^ – 100% | 3.1 (1.9-5.1)  I^2^ – 100% |
| >5.0 mmol/L | 8.7 (6.0-12.7)  I^2^ – 100% | 6.7 (4.4-10.3)  I^2^ – 100% |
| >5.5 mmol/L | 4.2 (2.4-7.2)  I^2^ – 100% | 2.8 (1.7-4.8)  I^2^ – 100% |
| >6.0 mmol/L | 1.6 (0.5-5.9)  I^2^ – 100% | 1.1 (0.2-4.5)  I^2^ – 100% |
| **South America** | 23.1 (4.7-52.1)  I^2^ – 99.2% | 18.8 (3.6-49.2)  I^2^ – 99.3% |
| **Global^^^** | 14.7 (4.1-28.1)  I^2^ – 100% | 15.4 (4.6-28.7)  I^2^ – 100% |

§ Includes patients with pre-dialysis CKD 5 (eGFR<15)

|| Includes patients from ANY study setting receiving kidney-replacement therapy but NOT pre-dialysis CKD5 or those with a kidney transplant

* Includes patients admitted to coronary care units and high dependency areas

+ Includes studies performed in an outpatient dialysis population and includes patients on both haemodialysis and peritoneal dialysis

# Includes patients taking ACE-Inhibitors, Angiotensin 2 receptor blockers, Renin inhibitors and Minerallo-corticoid receptor antagonists

^ Includes studies performed across different continents

**Reference list for all included studies**

1. Abbas, S., Ihle, P., Harder, S. & Schubert, I. Risk of hyperkalemia and combined use of spironolactone and long-term ACE inhibitor/angiotensin receptor blocker therapy in heart failure using real-life data: a population- and insurance-based cohort. *Pharmacoepidemiol. Drug Saf.* 24, 406–413 (2015).

2. Abbas, Z., Mumtaz, K., Salam, A. & Jafri, W. Factors predicting hyperkalemia in patients with cirrhosis receiving spironolactone. *J. Coll. Physicians Surg. Pak.* 13, 382–384 (2003).

3. Abensur Vuillaume, L. *et al.* Hypokalemia is frequent and has prognostic implications in stable patients attending the emergency department. *PLoS One* 15, e0236934 (2020).

4. Aboudara, M. C., Hurst, F. P., Abbott, K. C. & Perkins, R. M. Hyperkalemia after packed red blood cell transfusion in trauma patients. *J. Trauma* 64, S86–S91 (2008).

5. Abrich, V. A. *et al.* Clinical Outcomes of Various Management Strategies for Symptomatic Bradycardia. *Clin. Med. Res.* 18, 75–81 (2020).

6. Ackerman, B. H. *et al.* Trimethoprim-Induced Hyperkalemia in Burn Patients Treated With Intravenous or Oral Trimethoprim Sulfamethoxazole for Methicillin-Resistant Staphylococcus aureus and Other Infections. *J. Burn Care Res.* 34, 127–132 (2013).

7. Adelborg, K. *et al.* Predictors for repeated hyperkalemia and potassium trajectories in high-risk patients - A population-based cohort study. *PLoS One* 14, e0218739 (2019).

8. Adelmann, D. *et al.* Intraoperative Management of Liver Transplant Patients Without the Routine Use of Renal Replacement Therapy. *Transplantation* 102, e229–e235 (2018).

9. Adie, S. *et al.* Real-World Prevalence of Adverse Events After Initiating Sacubitril/Valsartan Compared With Angiotensin-Converting Enzyme Inhibitors or Angiotensin Receptor Blockers in Systolic Heart Failure. *J. Card. Fail.* 25, 412–413 (2019).

10. Adrish, M. *et al.* The Association of Renin-Angiotensin-Aldosterone System Inhibitors With Outcomes Among a Predominantly Ethnic Minority Patient Population Hospitalized With COVID-19: The Bronx Experience. *Cureus* 12, (2020).

11. Adwaney, A., Randall, D. W., Blunden, M. J., Prowle, J. R. & Kirwan, C. J. Perioperative Plasma-Lyte use reduces the incidence of renal replacement therapy and hyperkalaemia following renal transplantation when compared with 0.9% saline: a retrospective cohort study. *Clin. Kidney J.* 10, 838–844 (2017).

12. Aguilera Morales, W., BURGOS MARTIN, J., ALONSO GARCIA, F., Moyano Franco, M. J. & Salgueira Lazo, M. P0837HYPERKALEMIA IN RENAL PATIENTS, THE GREAT FORGOT? *Nephrol. Dial. Transplant.* 35, (2020).

13. Agustina, P. S., Yunir, E., Prawiroharjo, P., Damanik, J. & Sauriasari, R. Comparison of Effects of ACEIs and ARBs on Albuminuria and Hyperkalemia in Indonesian Hypertensive Type 2 Diabetes Mellitus Patients. *Int. J. Hypertens.* 2020, 1–8 (2020).

14. Ahmad, M., Wahid, A., Ahmad, M. & Mahboob, N. Prevalence of electrolyte disorders among cases of diarrhea with severe dehydration and correlation of electrolyte levels with age of the patients. *J. Coll. Physicians Surg. Pakistan* 26, 394–398 (2016).

15. Ahmed, M. I. *et al.* Mild hyperkalemia and outcomes in chronic heart failure: a propensity matched study. *Int. J. Cardiol.* 144, 383–388 (2010).

16. Ahuja, T. S. *et al.* Predictors of the development of hyperkalemia in patients using angiotensin-converting enzyme inhibitors. *Am. J. Nephrol.* 20, 268–272 (2000).

17. Ahuja, W., Kumar, N., Kumar, S. & Rizwan, A. Precipitating Risk Factors, Clinical Presentation, and Outcome of Diabetic Ketoacidosis in Patients with Type 1 Diabetes. *Cureus* 11, e4789 (2019).

18. Alappan, R., Perazella, M. A. & Buller, G. K. Hyperkalemia in hospitalized patients treated with trimethoprim-sulfamethoxazole. *Ann. Intern. Med.* 124, 316–320 (1996).

19. Aldahl, M. *et al.* Associations of serum potassium levels with mortality in chronic heart failure patients. *Eur. Heart J.* 38, 2890–2896 (2017).

20. Aloni, M., Nsibu, C., Meeko-Mimaniye, M., Ekulu, P. & Bodi, J. Acute renal failure in Congolese children: A tertiary institution experience. *Acta Paediatr. Int. J. Paediatr.* 101, e514–e518 (2012).

21. Amir, O. *et al.* Incidence of risk factors for developing hyperkalemia when using ACE inhibitors in cardiovascular diseases. *Pharm. World Sci.* 31, 387–393 (2009).

22. An, J. *et al.* Severe hyperkalemia requiring hospitalization: predictors of mortality. *Crit. Care* 16, R225 (2012).

23. Anees, M. *et al.* Referral pattern for hemodialysis patients to nephrologists. *J. Coll. Physicians Surg. Pak.* 17, 671–674 (2007).

24. Anton, C., Cox, A. R., Watson, R. D. S. & Ferner, R. E. The safety of spironolactone treatment in patients with heart failure. *J. Clin. Pharm. Ther.* 28, 285–287 (2003).

25. Antoniou, T. *et al.* Trimethoprim-sulfamethoxazole-induced hyperkalemia in patients receiving inhibitors of the renin-angiotensin system: A population-based study. *Arch. Intern. Med.* 170, 1045–1049 (2010).

26. Antoniou, T. *et al.* Trimethoprim–sulfamethoxazole and risk of sudden death among patients taking spironolactone. *Can. Med. Assoc. J.* 187, E138–E143 (2015).

27. Aoki, K. & Akaba, K. Characteristics of nonoliguric hyperkalemia in preterm infants: A case-control study in a single center. *Pediatr. Int.* 62, 576–580 (2020).

28. Arampatzis, S. *et al.* Impact of diuretic therapy-associated electrolyte disorders present on admission to the emergency department: A cross-sectional analysis. *BMC Med.* 11, 83 (2013).

29. Arora, P. *et al.* Preoperative serum potassium predicts the clinical outcome after non-cardiac surgery. *Clin. Chem. Lab. Med.* 55, 145–153 (2017).

30. Asadollahi, K., Hastings, I. M., Gill, G. V. & Beeching, N. J. Prediction of hospital mortality from admission laboratory data and patient age: A simple model. *Emerg. Med. Australas.* 23, 354–363 (2011).

31. Asante-Korang, A., Boyle, G. J., Webber, S. A., Miller, S. A. & Fricker, F. J. Experience of FK506 immune suppression in pediatric heart transplantation: a study of long-term adverse effects. *J. Heart Lung Transplant.* 15, 415–422 (1996).

32. Au, B. K. *et al.* Hyperkalemia Following Massive Transfusion in Trauma1. *J. Surg. Res.* 157, 284–289 (2009).

33. Bandak, G. *et al.* Hyperkalemia After Initiating Renin–Angiotensin System Blockade: The Stockholm Creatinine Measurements (SCREAM) Project. *J. Am. Heart Assoc.* 6, (2017).

34. Barbance, O. *et al.* Potassium disorders in pediatric emergency department: Clinical spectrum and management. *Arch. Pediatr.* 27, 146–151 (2020).

35. Beck, O. & Hochrein, H. Initial serum potassium level in relation to cardiac arrhythmias in acute myocardial infarction. *Z. Kardiol.* 66, 187–190 (1977).

36. Beilhack, G., Lindner, G., Funk, G.-C., Monteforte, R. & Schwarz, C. Electrolyte disorders in stable renal allograft recipients. *Swiss Med. Wkly.* 150, w20366 (2020).

37. Belmar Vega, L. *et al.* Epidemiology of hyperkalemia in chronic kidney disease. *Nefrologia* 39, 277–286 (2019).

38. Ben Mahmoud, L. *et al.* Prospective observational study of angiotensin converting enzyme inhibitors-induced hyperkalemia in hospitalized patients with chronic renal failure. *Nephrol. Ther.* 9, 98–102 (2013).

39. Betrosian, A. *et al.* Bacterial sepsis-induced rhabdomyolysis. *Intensive Care Med.* 25, 469–474 (1999).

40. Betts, K. *et al.* Postdischarge Healthcare Costs and Readmission in Patients With Hyperkalemia-Related Hospitalizations. *Kidney Int. Reports* (2020).

41. Betts, K. A. *et al.* The prevalence of hyperkalemia in the United States. *Curr. Med. Res. Opin.* 34, 971–978 (2018).

42. Betz, M., Steenes, A., Peterson, L. & Saunders, M. Knowledge Does Not Correspond to Adherence of Renal Diet Restrictions in Patients With Chronic Kidney Disease Stage 3-5. *J. Ren. Nutr.* 1–10 (2020). doi:10.1053/j.jrn.2020.08.007

43. Beusekamp, J. C. *et al.* Potassium and the use of renin-angiotensin-aldosterone system inhibitors in heart failure with reduced ejection fraction: data from BIOSTAT-CHF. *Eur. J. Heart Fail.* 20, 923–930 (2018).

44. Beusekamp, J. C. *et al.* Potassium abnormalities in patients with heart failure from 11 Asian regions: insights from the ASIAN‐HF registry. *Eur. J. Heart Fail.* 22, 751–754 (2020).

45. Bian, J., Zuo, L., Zhao, H. & Han, X. EPIDEMIOLOGY AND TREATMENT PATTERN OF HYPERKALAEMIA AMONG OUTPATIENTS IN CHINA: A DESCRIPTIVE STUDY USING AN ADMINISTRATIVE DATABASE IN CHINA. *Nephrol. Dial. Transplant.* 35, (2020).

46. Bird, J. G., McCully, R. B., Pellikka, P. A. & Kane, G. C. Dobutamine Stress Echocardiography: Impact of Abnormal Blood Potassium Levels on Cardiac Arrhythmias. *J. Am. Soc. Echocardiogr.* 30, 595–601 (2017).

47. Bird, S. T. *et al.* The association between drospirenone and hyperkalemia: a comparative-safety study. *BMC Clin. Pharmacol.* 11, 23 (2011).

48. Biswas, P. N., Wilton, L. V & Shakir, S. W. The safety of valsartan: results of a postmarketing surveillance study on 12 881 patients in England. *J. Hum. Hypertens.* 16, 795–803 (2002).

49. Blanc, A.-L. *et al.* Development of a predictive score for potentially avoidable hospital readmissions for general internal medicine patients. *PLoS One* 14, e0219348 (2019).

50. Bolotova, O. *et al.* Safety, tolerability, and outcomes of losartan use in patients hospitalized with SARS-CoV-2 infection: A feasibility study. *PLoS One* 15, e0244708 (2020).

51. Boo, A. Y. Y., Koh, Y. L. E., Hu, P. L. & Tan, N. C. Prevalence and factors associated with false hyperkalaemia in Asians in primary care: a cross-sectional study (the Unlysed Hyperkalaemia- the Unseen Burden (UHUB) study). *BMJ Open* 10, e033755 (2020).

52. Bookman, J. S., Romanelli, F., Hutzler, L., Bosco, J. A. & Lajam, C. The Utility and Cost Effectiveness of Immediate Postoperative Laboratory Studies in Hip and Knee Arthroplasty. *Bull. Hosp. Jt. Dis.* 77, 132–135 (2019).

53. Borra, S., Shaker, R. & Kleinfeld, M. Hyperkalemia in an adult hospitalized population. *Mt. Sinai J. Med.* 55, 226–229 (1988).

54. Bouadma, L. *et al.* Influence of dyskalemia at admission and early dyskalemia correction on survival and cardiac events of critically ill patients. *Crit. Care* 23, 415 (2019).

55. Bramlage, P. *et al.* Patient and disease characteristics of type-2 diabetes patients with or without chronic kidney disease: An analysis of the German DPV and DIVE databases. *Cardiovasc. Diabetol.* 18, 1–12 (2019).

56. Brion, L. P. *et al.* Early hyperkalaemia in very low birthweight infants in the absence of oliguria. *Arch. Dis. Child.* 64, 270–272 (1989).

57. Brookes, E. M., Snider, J., Hart, G. K., Robbins, R. & Power, D. A. Serum potassium in chronic kidney disease: prevalence, patient characteristics and clinical outcomes. *Intern. Med. J.* (2020). doi:10.1111/imj.14970

58. Browne, L. & Austin, S. PROFILING HYPOKALAEMIC AND HYPERKALAEMIC STATES IN THE IRISH HEALTH SYSTEM: TESTING RATES, INCIDENCE AND DETERMINANTS. *Nephrol. Dial. Transplant.* 35, (2020).

59. Brueske, B. *et al.* Hyperkalemia Is Associated With Increased Mortality Among Unselected Cardiac Intensive Care Unit Patients. *J. Am. Heart Assoc.* 8, e011814 (2019).

60. Brunelli, S. M., Du Mond, C., Oestreicher, N., Rakov, V. & Spiegel, D. M. Serum Potassium and Short-term Clinical Outcomes Among Hemodialysis Patients: Impact of the Long Interdialytic Interval. *Am. J. Kidney Dis.* 70, 21–29 (2017).

61. Bruno, N. *et al.* Mineralocorticoid receptor antagonists for heart failure: a real-life observational study. *ESC Hear. Fail.* 5, 267–274 (2018).

62. Buck, M. L. Clinical experience with spironolactone in pediatrics. *Ann. Pharmacother.* 39, 823–828 (2005).

63. Buckallew, A. R. *et al.* Evaluation of the safety and tolerability of spironolactone in patients with heart failure and chronic kidney disease. *Eur. J. Clin. Pharmacol.* 77, 955–960 (2021).

64. Bucsa, C., Moga, D. C., Farcas, A., Mogosan, C. & Dumitrascu, D. L. An investigation of the concomitant use of angiotensin-converting enzyme inhibitors, non-steroidal anti-inflammatory drugs and diuretics. *Eur. Rev. Med. Pharmacol. Sci.* 19, 2938–2944 (2015).

65. Cai, J.-J., Wang, K., Jiang, H.-Q. & Han, T. Characteristics, risk factors, and adverse outcomes of hyperkalemia in acute-on-chronic liver failure patients. *Biomed Res. Int.* 2019, 6025726 (2019).

66. Calvino, J. *et al.* Acute renal failure related to nonsteroidal antiinflammatory drugs and angiotensin-converting enzyme inhibitors. *Nefrologia* 17, 405–410 (1997).

67. Caravaca Perez, P. *et al.* Serum potassium dynamics during acute heart failure hospitalization. *Clin. Res. Cardiol.* (2020). doi:10.1007/s00392-020-01753-3

68. Caravaca-Fontan, F. *et al.* Association of hyperkalemia with clinical outcomes in advanced chronic kidney disease. *Asoc. entre hiperkaliemia y Evol. Clin. en la Enferm. Ren. Cron. Av.* 39, 513–522 (2019).

69. Caravaca-Fontán, F. *et al.* Renal potassium management in chronic kidney disease: Differences between patients with or without hyperkalemia. *Nefrol. (English Ed.* 40, 152–159 (2020).

70. Caulder, C. R., Kocherla, C. S., Qureshi, Z. P., Magagnoli, J. & Bookstaver, P. B. Dose-Dependent Hyperkalemia Among Hospitalized, HIV-Infected Patients Receiving Sulfamethoxazole/Trimethoprim. *Ann. Pharmacother.* 54, 852–857 (2020).

71. Chan, K. E. *et al.* Combined angiotensin-converting enzyme inhibition and receptor blockade associate with increased risk of cardiovascular death in hemodialysis patients. *Kidney Int.* 80, 978–985 (2011).

72. Chang, A. R. *et al.* Antihypertensive Medications and the Prevalence of Hyperkalemia in a Large Health System. *Hypertension* 67, 1181–1188 (2016).

73. Changlin, M. *et al.* Development of a hyperkalemia risk assessment model for patients with chronic kidney disease. *Natl. Med. J. China* 100, 3498–3503 (2020).

74. Chapin, E. *et al.* Adverse safety events in chronic kidney disease: The frequency of ‘Multiple Hits’. *Clin. J. Am. Soc. Nephrol.* 5, 95–101 (2010).

75. Chazard, E., Dumesnil, C. & Beuscart, R. How much does hyperkalemia lengthen inpatient stays? About methodological issues in analyzing time-dependant events. *Stud. Health Technol. Inform.* 210, 835–839 (2015).

76. Chazard, E., Luyckx, M., Beuscart, J.-B., Ferret, L. & Beuscart, R. Routine use of the ‘ADE scorecards’, an application for automated ADE detection in a general hospital. *Stud. Health Technol. Inform.* 192, 308–312 (2013).

77. Checherita, I. ., David, C., Diaconu, V., Ciocalteu, A. . & Lascar, I. Potassium level changes-arrhythmia contributing factor in chronic kidney disease patients. *Rom J Morphol Embryol* 52, 1047–1050 (2011).

78. Chen, T. *et al.* Clinical characteristics of 113 deceased patients with coronavirus disease 2019: Retrospective study. *BMJ* 368, m1091 (2020).

79. Chen, Y. *et al.* Serum Potassium, Mortality, and Kidney Outcomes in the Atherosclerosis Risk in Communities Study. *Mayo Clin. Proc.* 91, 1403–1412 (2016).

80. Chen, Y. *et al.* Race, Serum Potassium, and Associations With ESRD and Mortality. *Am. J. Kidney Dis.* 70, 244–251 (2017).

81. Cheng, M.-F. *et al.* Clinical characteristics and outcomes of new uremic patients with extreme azotemia in southern Taiwan. *Hemodial. Int.* 10, 294–302 (2006).

82. Cheungpasitporn, W. *et al.* Impact of admission serum potassium on mortality in patients with chronic kidney disease and cardiovascular disease. *QJM* 110, 713–719 (2017).

83. Chien, S.-C. *et al.* Comparative Effectiveness of Angiotensin-Converting Enzyme Inhibitors and Angiotensin II Receptor Blockers in Terms of Major Cardiovascular Disease Outcomes in Elderly Patients: A Nationwide Population-Based Cohort Study. *Med. (United States)* 94, e1751 (2015).

84. Chisti, M. J. *et al.* Hypoxaemia and septic shock were independent risk factors for mechanical ventilation in Bangladeshi children hospitalised for diarrhoea. *Acta Paediatr.* 106, 1159–1164 (2017).

85. Cho, J.-H., Hwang, J.-Y., Lee, S.-E., Jang, S. P. & Kim, W.-Y. Nutritional status and the role of diabetes mellitus in hemodialysis patients. *Nutr. Res. Pract.* 2, 301–307 (2008).

86. Choi, D. S., Shin, S. Do, Ro, Y. S. & LEE, K. W. Relationship between serum potassium level and survival outcome in out-of-hospital cardiac arrest using CAPTURES database of Korea: Does hypokalemia have good neurological outcomes in out-of-hospital cardiac arrest? *Adv. Clin. Exp. Med.* 29, 727–734 (2020).

87. Chon, S.-B., Kwak, Y. H., Hwang, S.-S., Oh, W. S. & Bae, J.-H. Severe hyperkalemia can be detected immediately by quantitative electrocardiography and clinical history in patients with symptomatic or extreme bradycardia: A retrospective cross-sectional study. *J. Crit. Care* 29, 310 (2014).

88. Chung, M.-Y. & Huang, C. B. Hyperkalemia in premature infants weighing less than 1,500 grams. *Clin. Neonatol.* 6, 1–5 (1999).

89. Cirillo, M. *et al.* Low glomerular filtration in the population: Prevalence, associated disorders, and awareness. *Kidney Int.* 70, 800–806 (2006).

90. Collins, A. J. *et al.* Association of Serum Potassium with All-Cause Mortality in Patients with and without Heart Failure, Chronic Kidney Disease, and/or Diabetes. *Am. J. Nephrol.* 46, 213–221 (2017).

91. Colombo, M. G. *et al.* Admission serum potassium concentration and long-term mortality in patients with acute myocardial infarction: results from the MONICA/KORA myocardial infarction registry. *BMC Cardiovasc. Disord.* 17, 198 (2017).

92. Conway, R., Creagh, D., Byrne, D. G., O’Riordan, D. & Silke, B. Serum potassium levels as an outcome determinant in acute medical admissions. *Clin. Med. (Northfield. Il).* 15, 239–243 (2015).

93. Cook, E., Davis, J. & Wu, E. Prevalence of metabolic acidosis among patients with CKD and hyperkalemia. *National Kidney Foundation Abstracts* (2020). Available at: https://casehippo.com/spa/symposium/national-kidney-foundation-2020-spring-clinical-meetings/event/gallery/abstracts?abstractId=930. (Accessed: 17th September 2020)

94. Cooper, L. B. *et al.* Association between potassium level and outcomes in heart failure with reduced ejection fraction: a cohort study from the Swedish Heart Failure Registry. *Eur. J. Heart Fail.* (2020).

95. Cooper, L. B. *et al.* Characterization of Mineralocorticoid Receptor Antagonist Therapy Initiation in High-Risk Patients With Heart Failure. *Circ. Cardiovasc. Qual. Outcomes* 10, e002946 (2017).

96. Cooper, L. B. *et al.* Use of Mineralocorticoid Receptor Antagonists in Patients With Heart Failure and Comorbid Diabetes Mellitus or Chronic Kidney Disease. *J. Am. Heart Assoc.* 6, (2017).

97. Cooper, W. D. *et al.* Cardiac arrhythmias following acute myocardial infarction: associations with the serum potassium level and prior diuretic therapy. *Eur. Heart J.* 5, 464–469 (1984).

98. Crellin, E. *et al.* Trimethoprim use for urinary tract infection and risk of adverse outcomes in older patients: cohort study. *BMJ* 360, k341 (2018).

99. Crespo-Leiro, M. G. *et al.* Hiperpotasemia en pacientes con insuficiencia cardiaca en España y su impacto en las recomendaciones. Registro ESC-EORP-HFA Heart Failure Long-Term. *Rev. Española Cardiol.* 73, 313–323 (2020).

100. Crook, M. A Study of Hypermagnesaemia in a Hospital Population. *Clin. Chem. Lab. Med.* 37, 449–451 (1999).

101. Cruz, C. S., Cruz, A. A. & Marcilio de Souza, C. A. Hyperkalaemia in congestive heart failure patients using ACE inhibitors and spironolactone. *Nephrol. Dial. Transplant.* 18, 1814–1819 (2003).

102. Cruz, C. S., Cruz, L. S., Silva, G. R. & Marcílio de Souza, C. A. Incidence and Predictors of Development of Acute Renal Failure Related to Treatment of Congestive Heart Failure with ACE Inhibitors. *Nephron Clin. Pract.* 105, c77–c83 (2006).

103. Cummings, B. M., Macklin, E. A., Yager, P. H., Sharma, A. & Noviski, N. Potassium abnormalities in a pediatric intensive care unit: frequency and severity. *J. Intensive Care Med.* 29, 269–274 (2014).

104. D’Alessandro, C. *et al.* Prevalence and correlates of hyperkalemia in a renal nutrition clinic. *Intern. Emerg. Med.* (2020).

105. Dalrymple, L. S. *et al.* Comparison of Hospitalization Rates among For-Profit and Nonprofit Dialysis Facilities. *Clin. J. Am. Soc. Nephrol.* 9, 73–81 (2014).

106. Dansirikul, C. *et al.* Relationships of tacrolimus pharmacokinetic measures and adverse outcomes in stable adult liver transplant recipients. *J. Clin. Pharm. Ther.* 31, 17–25 (2006).

107. Dantas, L. G. *et al.* Prevalence and predictors of nonadherence to hemodialysis. *Nephron. Clin. Pract.* 124, 67–71 (2013).

108. Dashputre, A., Sumida, K. & Kumar Potukuchi, P. Predictors of Hyperkalemia in Patients with Advanced CKD. in *American Society of Nephrology | Kidney Week - Abstract Details* (eds. Dashputre, A., Sumida, K., Potukuchi, P. K., Akbilgic, O. & Kovesdy, C. P.) (2019).

109. Davis, J., Done, N., Chamberlain, C. & Wu, E. Hyperkalemia and Progression of CKD. in *American Society of Nephrology - Kidney Week* (2019).

110. Dawwas, M. F. *et al.* The impact of serum potassium concentration on mortality after liver transplantation: a cohort multicenter study. *Transplantation* 88, 402–410 (2009).

111. Day, G. M., Radde, I. C., Balfe, J. W. & Chance, G. W. Electrolyte abnormalities in very low birthweight infants. *Pediatr. Res.* 10, 522–526 (1976).

112. Dayal, R. *et al.* A clinico-hematological profile of hemolytic-uremic syndrome. *Southeast Asian J. Trop. Med. Public Health* 24, 280–283 (1993).

113. de Denus, S. *et al.* Quantification of the risk and predictors of hyperkalemia in patients with left ventricular dysfunction. *Am. Heart J.* 152, 705–712 (2006).

114. De Rooij, E., Dekker, F. W., Le Cessie, S., De Fijter, J. W. & Hoogeveen, E. K. ESPECIALLY HYPOKALEMIA IS A RISK FACTOR FOR ALL-CAUSE MORTALITY IN INCIDENT HEMODIALYSIS PATIENTS. *Nephrol. Dial. Transplant.* 35, (2020).

115. De Vecchis, R., Ariano, C., Di Biase, G. & Noutsias, M. Sacubitril/valsartan for heart failure with reduced left ventricular ejection fraction. *Herz* 44, 425–432 (2019).

116. de Vries, B. C. S., Berger, S. P., Bakker, S. J. L., de Borst, M. H. & de Jong, M. F. C. Pre-Transplant Plasma Potassium as a Potential Risk Factor for the Need of Early Hyperkalaemia Treatment after Kidney Transplantation: A Cohort Study. *Nephron* 145, 63–70 (2021).

117. Degli Esposti, L. *et al.* Effect of hyperkalemia and RAASi nonadherence on patients affected by heart failure or chronic kidney disease. *G. Ital. Nefrol.* 36, (2019).

118. Derington, C. G. *et al.* Twice-daily versus once-daily lisinopril and losartan for hypertension: Real-world effectiveness and safety. *PLoS One* 15, e0243371 (2020).

119. Desai, N. R. *et al.* The economic implications of hyperkalemia in a medicaid managed care population. *Am. Heal. Drug Benefits* 12, 352–361 (2019).

120. Deskur-Smielecka, E. *et al.* Use of renal risk drugs in a nation-wide Polish older adult population: an analysis of PolSenior database. *BMC Geriatr.* 19, 70 (2019).

121. Diamantidis, C. J. *et al.* A varying patient safety profile between black and nonblack adults with decreased estimated GFR. *Am. J. Kidney Dis.* 60, 47–53 (2012).

122. Dickson, S. J. *et al.* Enhanced case management can be delivered for patients with EVD in Africa: Experience from a UK military Ebola treatment centre in Sierra Leone. *J. Infect.* 76, 383–392 (2018).

123. Doenyas-Barak, K., Beberashvili, I. & Vinker, S. Serum potassium is an age-dependent risk factor for pre-diabetes and diabetes in the Israeli population. *Diabetes Vasc. Dis. Res.* 11, 103–109 (2014).

124. Dolores, C., Manuel, C., Francisco, M. & Miguel, F. Evaluation of use, efficacy and safety of sacubitril/valsartan. *Eur. J. Clin. Pharm.* 21, 144–148 (2019).

125. Douvris, A. *et al.* Safety Lapses Prior to Initiation of Hemodialysis for Acute Kidney Injury in Hospitalized Patients: A Patient Safety Initiative. *J. Clin. Med.* 7, 317 (2018).

126. Drawz, P. E., Babineau, D. C. & Rahman, M. Metabolic Complications in Elderly Adults with Chronic Kidney Disease. *J. Am. Geriatr. Soc.* 60, 310–315 (2012).

127. Drion, I., Joosten, H., Dikkeschei, L. D., Groenier, K. H. & Bilo, H. J. G. eGFR and creatinine clearance in relation to metabolic changes in an unselected patient population. *Eur. J. Intern. Med.* 20, 722–727 (2009).

128. Duran, J. M., Gad, S., Brann, A. & Greenberg, B. Mineralocorticoid receptor antagonist use following heart failure hospitalization. *ESC Hear. Fail.* 7, 482–492 (2020).

129. Einhorn, L. M. *et al.* The Frequency of Hyperkalemia and Its Significance in Chronic Kidney Disease. *Arch. Intern. Med.* 169, 1156 (2009).

130. Eliacik, E. *et al.* Potassium abnormalities in current clinical practice: Frequency, causes, severity and management. *Med. Princ. Pract.* 24, 271–275 (2015).

131. Elmaasarani, Z. *et al.* Protocol-based nurse coordinator management of ambulatory tacrolimus dosing in de novo renal transplant recipients-A single-center experience with a large African American population. *Clin. Transplant.* 33, e13701 (2019).

132. Engbaek, M., Hjerrild, M., Hallas, J. & Jacobsen, I. A. The effect of low-dose spironolactone on resistant hypertension. *J. Am. Soc. Hypertens.* 4, 290–294 (2010).

133. Engwerda, E., van den Berg, M., Blans, M., Bech, A. & de Boer, H. Efficacy and safety of a phosphate replacement strategy for severe hypophosphatemia in the ICU. *Neth. J. Med.* 76, 437–441 (2018).

134. Epstein, M. *et al.* Evaluation of the treatment gap between clinical guidelines and the utilization of renin-angiotensin-aldosterone system inhibitors. *Am. J. Manag. Care* 21, S212-20 (2015).

135. Erek, E. *et al.* An overview of morbidity and mortality in patients with acute renal failure due to crush syndrome: the Marmara earthquake experience. *Nephrol. Dial. Transplant.* 17, 33–40 (2002).

136. Eriguchi, R. *et al.* Racial and Ethnic Differences in Mortality Associated with Serum Potassium in Incident Peritoneal Dialysis Patients. *Am. J. Nephrol.* 50, 361–369 (2019).

137. Eschalier, R. *et al.* Safety and Efficacy of Eplerenone in Patients at High Risk for Hyperkalemia and/or Worsening Renal Function. *J. Am. Coll. Cardiol.* 62, 1585–1593 (2013).

138. Eschmann, E., Beeler, P. E., Schneemann, M. & Blaser, J. Developing strategies for predicting hyperkalemia in potassium-increasing drug-drug interactions. *J. Am. Med. Informatics Assoc.* 24, 60–66 (2017).

139. Falk, R. S. *et al.* Fasting Serum Levels of Potassium and Sodium in Relation to Long-Term Risk of Cancer in Healthy Men. *Clin. Epidemiol.* Volume 12, 1–8 (2020).

140. Fang, G. *et al.* Incidence of and Risk Factors for Severe Adverse Events in Elderly Patients Taking Angiotensin-Converting Enzyme Inhibitors or Angiotensin II Receptor Blockers after an Acute Myocardial Infarction. *Pharmacother. J. Hum. Pharmacol. Drug Ther.* 38, 29–41 (2018).

141. Faulkner, M., Mears, A. J., Adams, P. M., Frock, J. T. & Dunlay, R. W. Can Selective Beta-Blockers Be Prescribed Safely for Hemodialysis Patients? A Retrospective Review. *Hosp. Pharm.* 39, 144–148 (2004).

142. Faxen, J. *et al.* Potassium levels and risk of in-hospital arrhythmias and mortality in patients admitted with suspected acute coronary syndrome. *Int. J. Cardiol.* 274, 52–58 (2019).

143. Ficheur, G. *et al.* Adverse drug events with hyperkalaemia during inpatient stays: evaluation of an automated method for retrospective detection in hospital databases. *BMC Med. Inform. Decis. Mak.* 14, 83 (2014).

144. Fitch, K., Woolley, J. M., Engel, T. & Blumen, H. The Clinical and Economic Burden of Hyperkalemia on Medicare and Commercial Payers. *Am. Heal. Drug Benefits* 10, 202–210 (2017).

145. Fleet, J. L. *et al.* Validity of the International Classification of Diseases 10th revision code for hyperkalaemia in elderly patients at presentation to an emergency department and at hospital admission. *BMJ Open* 2, e002011 (2012).

146. Formica, R. N., Friedman, A. L., Lorber, M. I. & Bia, M. J. Angiotensin-converting enzyme inhibitors and angiotensin II receptor blockers used for the treatment of hypertension appear to be safe in the early posttransplant period. *Transplant. Proc.* 36, 2675–2678 (2004).

147. Formiga, F. *et al.* Influence of potassium levels on one-year outcomes in elderly patients with acute heart failure. *Eur. J. Intern. Med.* 60, 24–30 (2019).

148. Frohlich, H., Nelges, C., Tager, T., Katus, H. A. & Frankenstein, L. Association between long-term changes of renal function and ACE-Inhibitor/ angiotensin receptor blocker dosing in patients with cardiorenal syndrome. *Nieren- und Hochdruckkrankheiten* 48, 288–293 (2019).

149. Frohlich, H. *et al.* Long-term changes of renal function in relation to ace inhibitor/angiotensin receptor blocker dosing in patients with heart failure and chronic kidney disease. *Am. Heart J.* 178, 28–36 (2016).

150. Furuland, H. *et al.* Serum potassium as a predictor of adverse clinical outcomes in patients with chronic kidney disease: new risk equations using the UK clinical practice research datalink. *BMC Nephrol.* 19, 211 (2018).

151. Gallo-Bernal, S., Hernandez-Linares, I. & Rodriguez Maria, J. Incidence of Hyperkalemia in a Referral Center for Latin-American Cardiology: It is Time for New Therapies. *Circulation* 140, A17249–A17249 (2019).

152. Gallo-Bernal, S. *et al.* Impact of a pharmacist-based multidimensional intervention aimed at decreasing the risk of hyperkalemia in heart failure patients: A Latin-American experience. *Int. J. Cardiol.* 329, 136–143 (2021).

153. Galloway, C. D. *et al.* Development and Validation of a Deep-Learning Model to Screen for Hyperkalemia From the Electrocardiogram. *JAMA Cardiol.* 4, 428–436 (2019).

154. Gao, X.-P. *et al.* Admission serum sodium and potassium levels predict survival among critically ill patients with acute kidney injury: a cohort study. *BMC Nephrol.* 20, 311 (2019).

155. Garg, N. *et al.* Efficacy and outcome of intermittent peritoneal dialysis in patients with acute kidney injury: A single-center experience. *Saudi J. Kidney Dis. Transplant.* 31, 423–430 (2020).

156. Garlo, K. G., Bates, D. W., Seger, D. L., Fiskio, J. M. & Charytan, D. M. Association of Changes in Creatinine and Potassium Levels After Initiation of Renin Angiotensin Aldosterone System Inhibitors With Emergency Department Visits, Hospitalizations, and Mortality in Individuals With Chronic Kidney Disease. *JAMA Netw. Open* 1, e183874 (2018).

157. Gatti, M., Antonazzo, I. C., Diemberger, I., De Ponti, F. & Raschi, E. Adverse events with sacubitril/valsartan in the real world: emerging signals to target preventive strategies from the FDA adverse event reporting system. *Eur. J. Prev. Cardiol.* 204748732091566 (2020). doi:10.1177/2047487320915663

158. Genovesi, S. *et al.* Sudden death and associated factors in a historical cohort of chronic haemodialysis patients. *Nephrol. Dial. Transplant.* 24, 2529–2536 (2009).

159. Gentry, C. A. & Nguyen, A. T. An Evaluation of Hyperkalemia and Serum Creatinine Elevation Associated With Different Dosage Levels of Outpatient Trimethoprim-Sulfamethoxazole With and Without Concomitant Medications. *Ann. Pharmacother.* 47, 1618–1626 (2013).

160. Gheno, G. *et al.* Variations of serum potassium level and risk of hyperkalemia in inpatients receiving low-molecular-weight heparin. *Eur. J. Clin. Pharmacol.* 59, 373–377 (2003).

161. Gilbert, C. J. *et al.* No Increase in Adverse Events During Aliskiren Use Among Ontario Patients Receiving Angiotensin-Converting Enzyme Inhibitors or Angiotensin-Receptor Blockers. *Can. J. Cardiol.* 29, 586–591 (2013).

162. Gill, K. *et al.* Red blood cell transfusion, hyperkalemia, and heart failure in advanced chronic kidney disease. *Pharmacoepidemiol. Drug Saf.* 24, 654–662 (2015).

163. Ginsberg, J. S. *et al.* Patient-Reported and Actionable Safety Events in CKD. *J. Am. Soc. Nephrol.* 25, 1564–1573 (2014).

164. Giordano, M. *et al.* Diseases associated with electrolyte imbalance in the ED: age-related differences. *Am. J. Emerg. Med.* 34, 1923–1926 (2016).

165. Goland, S. *et al.* Appropriateness and complications of the use of spironolactone in patients treated in a heart failure clinic. *Eur. J. Intern. Med.* 22, 424–427 (2011).

166. Golestaneh, L. *et al.* All-cause costs increase exponentially with increased chronic kidney disease stage. *Am. J. Manag. Care* 23, S163–S172 (2017).

167. Goncalves, F. A. *et al.* Hypokalemia and hyperkalemia in patients on peritoneal dialysis: incidence and associated factors. *Int. Urol. Nephrol.* 52, 393–398 (2020).

168. González-Ortiz, A. *et al.* Nutritional status, hyperkalaemia and attainment of energy/protein intake targets in haemodialysis patients following plant-based diets: a longitudinal cohort study. *Nephrol. Dial. Transplant.* 36, 681–688 (2021).

169. Gosavi, S. *et al.* Effect of Haemodialysis on QTc in Newly Diagnosed Chronic Kidney Disease Patients. *J. Clin. DIAGNOSTIC Res.* 14, 15–17 (2020).

170. Gosmanova, E. O. *et al.* Longer Predialysis ACEi/ARB Utilization Is Associated With Reduced Postdialysis Mortality. *Am. J. Med.* (2020).

171. Graber, M., Subramani, K., Corish, D. & Schwab, A. Thrombocytosis elevates serum potassium. *Am. J. Kidney Dis.* 12, 116–120 (1988).

172. Grandy, S. *et al.* Assessing the Impact of Hyperkalemia on Patient-Reported Quality of Life: A Global Analysis of the KDQOL-36 in a Real-World CKD Patient Population. in *American Society of Nephrology | Kidney Week - Abstract Details* (2018).

173. Grodzinsky, A. *et al.* Prevalence and Prognosis of Hyperkalemia in Patients with Acute Myocardial Infarction. *Am. J. Med.* 129, 858–865 (2016).

174. Gruskay, J., Costarino, A. T., Polin, R. A. & Baumgart, S. Nonoliguric hyperkalemia in the premature infant weighing less than 1000 grams. *J. Pediatr.* 113, 381–386 (1988).

175. Gwoo, S., Kim, Y. N., Shin, H. S., Jung, Y. S. & Rim, H. Predictors of hyperkalemia risk after hypertension control with aldosterone blockade according to the presence or absence of chronic kidney disease. *Nephron. Clin. Pract.* 128, 381–386 (2014).

176. Haas, J. S. *et al.* The burden of hyperkalemia in Germany – a real world evidence study assessing the treatment and costs of hyperkalemia. *BMC Nephrol.* 21, 332 (2020).

177. Hagengaard, L. *et al.* Association between serum potassium levels and short-term mortality in patients with atrial fibrillation or flutter co-treated with diuretics and rate- or rhythm-controlling drugs. *Eur. Hear. J. - Cardiovasc. Pharmacother.* 6, 137–144 (2020).

178. Hahn, B., Wranze, E., Wulf, H. & Kill, C. Point-of-Care-Lab Investigation in Prehospital Emergency Medicine A Profit of Information for Emergency Care on Scene? *Notarzt* 32, 117–121 (2016).

179. Hawkins, R. C. Gender and age as risk factors for hypokalemia and hyperkalemia in a multiethnic Asian population. *Clin. Chim. Acta* 331, 171–172 (2003).

180. Hayajneh, W. A., Jdaitawi, H., Al Shurman, A. & Hayajneh, Y. A. Comparison of Clinical Associations and Laboratory Abnormalities in Children With Moderate and Severe Dehydration. *J. Pediatr. Gastroenterol. Nutr.* 50, 290–294 (2010).

181. Hayes, J. *et al.* Association of Hypo- and hyperkalemia with disease progression and mortality in males with chronic kidney disease: The role of race. *Nephron - Clin. Pract.* 120, C8–C16 (2012).

182. Hayes, W. *et al.* Plasma electrolyte imbalance in pediatric kidney transplant recipients. *Pediatr. Transplant.* 23, e13411 (2019).

183. Hecking, E. *et al.* Haemodialysis prescription, adherence and nutritional indicators in five European countries: results from the Dialysis Outcomes and Practice Patterns Study (DOPPS). *Nephrol. Dial. Transplant.* 19, 100–107 (2004).

184. Henz, S. *et al.* Influence of drugs and comorbidity on serum potassium in 15 000 consecutive hospital admissions. *Nephrol. Dial. Transplant.* 23, 3939–3945 (2008).

185. Hernandez, A. F. *et al.* Associations between aldosterone antagonist therapy and risks of mortality and readmission among patients with heart failure and reduced ejection fraction. *JAMA - J. Am. Med. Assoc.* 308, 2097–2107 (2012).

186. Heshka, J., Ruzicka, M., Hiremath, S. & McCormick, B. B. Spironolactone for difficult to control hypertension in chronic kidney disease: an analysis of safety and efficacy. *J. Am. Soc. Hypertens.* 4, 295–301 (2010).

187. Hessels, L. *et al.* The relationship between serum potassium, potassium variability and in-hospital mortality in critically ill patients and a before-after analysis on the impact of computer-assisted potassium control. *Crit. Care* 19, 4 (2015).

188. Higashioka, K. *et al.* Renal Insufficiency in Concert with Renin-angiotensin-aldosterone Inhibition Is a Major Risk Factor for Hyperkalemia Associated with Low-dose Trimethoprim-sulfamethoxazole in Adults. *Intern. Med.* 55, 467–471 (2016).

189. Hirai, T., Yamaga, R., Fujita, A. & Itoh, T. Low body mass index is a risk factor for hyperkalaemia associated with angiotensin converting enzyme inhibitors and angiotensin II receptor blockers treatments. *J. Clin. Pharm. Ther.* 43, 829–835 (2018).

190. Hirsch, S., Hirsch, J., Bhatt, U. & Rovin, B. . Tolerating increases in the serum creatinine following aggressive treatment of chronic kidney disease, hypertension and proteinuria: Pre-renal success. *Am. J. Nephrol.* 36, 430–437 (2012).

191. Horne, L. *et al.* Epidemiology and health outcomes associated with hyperkalemia in a primary care setting in England. *BMC Nephrol.* 20, 85 (2019).

192. Hoss, S. *et al.* Serum Potassium Levels and Outcome in Patients With Chronic Heart Failure. *Am. J. Cardiol.* 118, 1868–1874 (2016).

193. Hsu, F.-Y., Lin, F.-J., Ou, H.-T., Huang, S.-H. & Wang, C.-C. Renoprotective Effect of Angiotensin-Converting Enzyme Inhibitors and Angiotensin II Receptor Blockers in Diabetic Patients with Proteinuria. *Kidney Blood Press. Res.* 42, 358–368 (2017).

194. Hsu, T.-W. *et al.* Renoprotective effect of renin-angiotensin-aldosterone system blockade in patients with predialysis advanced chronic kidney disease, hypertension,and anemia. *JAMA Intern. Med.* 174, 347–354 (2014).

195. Huang, C.-W. *et al.* Low Potassium Dialysate as a Protective Factor of Sudden Cardiac Death in Hemodialysis Patients with Hyperkalemia. *PLoS One* 10, e0139886 (2015).

196. Hughes-Austin, J. M. *et al.* The Relation of Serum Potassium Concentration with Cardiovascular Events and Mortality in Community-Living Individuals. *Clin. J. Am. Soc. Nephrol.* 12, 245–252 (2017).

197. Humphrey, T. J. L., Wilkinson, I. B. & Hiemstra, T. F. ACUTE TREATMENT OF HYPERKALAEMIA WITH INTRAVENOUS INSULIN – COMPARATIVE EVALUATION OF PATIENT CHARACTERISTICS. *Nephrol. Dial. Transplant.* 34, (2019).

198. Humphrey, T., Torpey, N. & Hiemstra, T. HYPERKALAEMIA IN PREVALENT KIDNEY TRANSPLANT RECIPIENTS. *Nephrol. Dial. Transplant.* 35, (2020).

199. Hung, K. C., Su, B. H., Lin, T. W., Peng, C. T. & Tsai, C. H. Glucose-insulin infusion for the early treatment of non-oliguric hyperkalemia in extremely-low-birth-weight infants. *Acta Paediatr. Taiwanica* 42, 282–286 (2001).

200. Hvidt, S., Bechmann, T., Behrndtz, N. H., Dorph-Petersen, A. & Madsen, G. Hyperkalemia among 10,074 patients admitted to a medical department. *Ugeskr. Laeger* 148, 1208–1212 (1986).

201. Hwang, J.-C., Wang, C.-T., Chen, C.-A. & Chen, H.-C. Hypokalemia is associated with increased mortality rate in chronic hemodialysis patients. *Blood Purif.* 32, 254–261 (2011).

202. Ideguchi, T., Tsuruda, T., Sato, Y. & Kitamura, K. Coexisting Hyponatremia and Decline in Diastolic Blood Pressure Predispose to Atrial Standstill in Hyperkalemic Patients. *Circ. J.* 80, 1781–1786 (2016).

203. Igiraneza, G., Ndayishimiye, B., Nkeshimana, M., Dusabejambo, V. & Ogbuagu, O. Clinical Profile and Outcome of Patients with Acute Kidney Injury Requiring Hemodialysis: Two Years’ Experience at a Tertiary Hospital in Rwanda. *Biomed Res. Int.* 2018, 1716420 (2018).

204. Iseki, K. *et al.* Impact of the initial levels of laboratory variables on survival in chronic dialysis patients. *Am. J. Kidney Dis.* 28, 541–548 (1996).

205. Ismail Hassan, K., Hodan M, J. & Li, C. A Retrospective Study of Acute Renal Failure in Children: Its Incidence, Etiology, Complications and Prognosis. *Cureus* 9, e1274 (2017).

206. Ito, H., Fujimaki, H., Inoue, J. & Shiraki, M. Disorders of fluid and electrolyte metabolism in elderly diabetics. *Nihon Ronen Igakkai Zasshi.* 26, 233–239 (1989).

207. Jacob, J. *et al.* Acute heart failure and adverse events associated with the presence of renal dysfunction and hyperkalaemia. EAHFE- renal dysfunction and hyperkalaemia. *Eur. J. Intern. Med.* 67, 89–96 (2019).

208. Jain, A. B. *et al.* Long-term results of cyclosporine in cadaveric renal transplantation from a single center. *Transplant. Proc.* 20, 82–85 (1988).

209. Jain, A. B., Kashyap, R., Rakela, J., Starzl, T. E. & Fung, J. J. Primary adult liver transplantation under tacrolimus: More than 90 months actual follow-up survival and adverse events. *Liver Transplant. Surg.* 5, 144–150 (1999).

210. Jain, A. *et al.* What have we learned about primary liver transplantation under tacrolimus immunosuppression? Long-term follow-up of the first 1000 patients. *Ann. Surg.* 230, 441–449 (1999).

211. Jain, N. *et al.* Predictors of Hyperkalemia and Death in Patients With Cardiac and Renal Disease. *Am. J. Cardiol.* 109, 1510–1513 (2012).

212. Jarman, P. R., Kehely, A. M. & Mather, H. M. Hyperkalaemia in diabetes: prevalence and associations. *Postgrad. Med. J.* 71, 551–552 (1995).

213. Jebali, H. *et al.* Evaluation of electrocardiographic findings before and after hemodialysis session. *Saudi J. Kidney Dis. Transplant.* 31, 639–646 (2020).

214. Jha, C. M., Chatterjee, A. R. & Dastoor, H. D. Epidemiology of Sodium and Potassium Electrolyte Imbalance in Ambulatory Care in United Arab Emirates: A Single Centre Study. in *American Society of Nephrology | Kidney Week - Abstract Details* (2018).

215. Jiménez-Marrero, S. *et al.* Real-World Epidemiology of Potassium Derangements Among Chronic Cardiovascular, Metabolic and Renal Conditions: A Population-Based Analysis. *Clin. Epidemiol.* Volume 12, 941–952 (2020).

216. Jimenez-Marrero, S. *et al.* Impact on clinical outcomes and health costs of deranged potassium levels in patients with chronic cardiovascular, metabolic, and renal conditions. *Rev. Esp. Cardiol. (Engl. Ed).* (2020).

217. Jin, A. *et al.* Normal range of serum potassium, prevalence of dyskalaemia and associated factors in Chinese older adults: a cross-sectional study. *BMJ Open* 10, e039472 (2020).

218. Johnson, E. S. *et al.* Predicting the risk of hyperkalemia in patients with chronic kidney disease starting lisinopril. *Pharmacoepidemiol. Drug Saf.* 19, 266–272 (2010).

219. Juang, S.-E. *et al.* Predictive Risk Factors in the Development of Intraoperative Hyperkalemia in Adult Living Donor Liver Transplantation. *Transplant. Proc.* 48, 1022–1024 (2016).

220. Jun, M. *et al.* Hyperkalemia and renin-angiotensin aldosterone system inhibitor therapy in chronic kidney disease: A general practice-based, observational study. *PLoS One* 14, e0213192 (2019).

221. Jun, M. *et al.* Hyperkalemia and renin-angiotensin aldosterone system inhibitor therapy in chronic kidney disease: A general practice-based, observational study. *PLoS One* 14, e0213192 (2019).

222. Juurlink, D. N. Drug-Drug Interactions Among Elderly Patients Hospitalized for Drug Toxicity. *JAMA* 289, 1652 (2003).

223. Kamath, S. U., Patil, B., Shelke, U. & Patwardhan, S. K. Comparing diabetic and nondiabetic emphysematous pyelonephritis and evaluating predictors of mortality. *Saudi J. Kidney Dis. Transplant.* 30, 1266–1275 (2019).

224. Kanda, E., Ai, M., Kuriyama, R., Yoshida, M. & Shiigai, T. Dietary Acid Intake and Kidney Disease Progression in the Elderly. *Am. J. Nephrol.* 39, 145–152 (2014).

225. Kanda, E., Kashikara, N., Kohsaka, S., Okami, S. & Yajima, T. Long-Term Healthcare Cost and Resource Use in Patients with Hyperkalemia. in *American Society of Nephrology - Kidney Week* (2019).

226. Kapelios, C. J. *et al.* Association Between High-Dose Spironolactone and Decongestion in Patients with Acute Heart Failure: An Observational Retrospective Study. *Am. J. Cardiovasc. Drugs* 18, 415–422 (2018).

227. Kaplan, B. *et al.* FREQUENCY OF HYPERKALEMIA IN RECIPIENTS OF SIMULTANEOUS PANCREAS AND KIDNEY TRANSPLANTS WITH BLADDER DRAINAGE. *Transplantation* 62, 1174,1175 (1996).

228. Karaboyas, A., Robinson, B., James, G., Hedman, K. & Pecoits-Filho, R. Hyperkalemia Excursions and Mortality in Hemodialysis Patients: Results from the DOPPSn Society of Nephrology | Kidney Week - Abstract Details. in *American Society of Nephrology - Kidney Week* (2019).

229. Karaboyas, A. *et al.* DOPPS data suggest a possible survival benefit of renin angiotensin-aldosterone system inhibitors and other antihypertensive medications for hemodialysis patients. *Kidney Int.* 94, 589–598 (2018).

230. Karaboyas, A. *et al.* Dialysate Potassium, Serum Potassium, Mortality, and Arrhythmia Events in Hemodialysis: Results From the Dialysis Outcomes and Practice Patterns Study (DOPPS). *Am. J. Kidney Dis.* 69, 266–277 (2017).

231. Karuppan, A. *et al.* Electrolyte disturbances among diabetic patients admitted in a multi-specialty hospital in southern India. *J. Clin. Diagnostic Res.* 13, OC12–OC15 (2019).

232. Kashihara, N. *et al.* Hyperkalemia in Real-World Patients Under Continuous Medical Care in Japan. *Kidney Int. Reports* 4, 1248–1260 (2019).

233. Kaya, A., Keskin, M., Tatlisu, M. A. & Kayapinar, O. Effect of Dynamic Potassium Change on In-Hospital Mortality, Ventricular Arrhythmias, and Long-Term Mortality in STEMI. *Angiology* 70, 69–77 (2019).

234. Keskin, M. *et al.* The effect of serum potassium level on in-hospital and long-term mortality in ST elevation myocardial infarction. *Int. J. Cardiol.* 221, 505–510 (2016).

235. Khedr, E., Abdelwhab, S., El-Sharkawy, M., Ali, M. & El Said, K. Prevalence of hyperkalemia among hemodialysis patients in Egypt. *Ren. Fail.* 31, 891–898 (2009).

236. Khorasani, B., Gholizadeh Pasha, A. & Khorasani, M. Evaluating mortality rate caused by electrolyte abnormalities in patients hospitalized. *Acta Med. Iran.* 46, 141–148 (2008).

237. Kieneker, L. M. *et al.* Plasma potassium, diuretic use and risk of developing chronic kidney disease in a predominantly White population. *PLoS One* 12, e0174686 (2017).

238. Kilbride, H. W., Cater, G., Warady, B. A., H.W., K. & G., C. Early onset hyperkalemia in extremely low birth weight infants. *J. Perinatol.* 8, 211–214 (1988).

239. Kim, H. C. *et al.* Primary immunosuppression with tacrolimus in kidney transplantation: Three-year follow-up in a single center. *Transplant. Proc.* 36, 2082–2083 (2004).

240. Kim, H. Y., Bae, E. H., Ma, S. K. & Kim, S. W. Effects of spironolactone in combination with angiotensin-converting enzyme inhibitors or angiotensin receptor blockers in patients with proteinuria. *Kidney Blood Press. Res.* 39, 573–580 (2014).

241. Kim, H. W., Lee, D. H., Lee, S. A. & Koh, G. A relationship between serum potassium concentration and insulin resistance in patients with type 2 diabetes mellitus. *Int. Urol. Nephrol.* 47, 991–999 (2015).

242. Kim, K. *et al.* Healthcare resource utilisation and cost associated with elevated potassium levels: A Danish population-based cohort study. *BMJ Open* 9, e026465 (2019).

243. Kim, T. *et al.* Racial and Ethnic Differences in Mortality Associated with Serum Potassium in a Large Hemodialysis Cohort. *Am. J. Nephrol.* 45, 509–521 (2017).

244. Knoll, G. A. *et al.* Renin-angiotensin system blockade and the risk of hyperkalemia in chronic hemodialysis patients. *Am. J. Med.* 112, 110–114 (2002).

245. Ko, D. T. *et al.* Appropriateness of Spironolactone Prescribing in Heart Failure Patients: A Population-Based Study. *J. Card. Fail.* 12, 205–210 (2006).

246. Kohsaka, S., Okami, S., Kanda, E., Kashihara, N. & Yajima, T. Cardiovascular and Renal Outcomes Associated With Hyperkalemia in Chronic Kidney Disease: A Hospital-Based Cohort Study. *Mayo Clin. Proc. Innov. Qual. Outcomes* 5, 274–285 (2021).

247. Koivunen, R.-J. *et al.* Medical Acute Complications of Intracerebral Hemorrhage in Young Adults. *Stroke Res. Treat.* 2015, 1–7 (2015).

248. Koren-Michowitz, M. *et al.* Early onset of hyperkalemia in patients treated with low molecular weight heparin: A prospective study. *Pharmacoepidemiol. Drug Saf.* 13, 299–302 (2004).

249. Korgaonkar, S. *et al.* Serum potassium and outcomes in CKD: insights from the RRI-CKD cohort study. *Clin. J. Am. Soc. Nephrol.* 5, 762–769 (2010).

250. Kovesdy, C. P. *et al.* Serum potassium and adverse outcomes across the range of kidney function: a CKD Prognosis Consortium meta-analysis. *Eur. Heart J.* 39, 1535–1542 (2018).

251. Krijthe, B. P. *et al.* Serum potassium levels and the risk of atrial fibrillation: the Rotterdam Study. *Int. J. Cardiol.* 168, 5411–5415 (2013).

252. Krogager, M. L. *et al.* Impact of plasma potassium normalization on short-term mortality in patients with hypertension and hypokalemia or low normal potassium. *BMC Cardiovasc. Disord.* 20, 386 (2020).

253. Krogager, M. L. *et al.* Short-term mortality risk of serum potassium levels in hypertension: a retrospective analysis of nationwide registry data. *Eur. Heart J.* 38, 104–112 (2017).

254. Kromah, F. *et al.* Relative adrenal insufficiency in the critical care setting: debunking the classic myth. *World J. Surg.* 35, 1818–1823 (2011).

255. Ku, L. C. *et al.* Safety of Enalapril in Infants Admitted to the Neonatal Intensive Care Unit. *Pediatr. Cardiol.* 38, 155–161 (2017).

256. Kuijvenhoven, M. A., Haak, E. A. F., Gombert-Handoko, K. B. & Crul, M. Evaluation of the concurrent use of potassium-influencing drugs as risk factors for the development of hyperkalemia. *Int. J. Clin. Pharm.* 35, 1099–1104 (2013).

257. Kulkarni, G., Rao, S., Netto, A., Taly, A. & Uma Maheshwara Rao, G. Prognosis of patients with Guillain-Barré syndrome requiring mechanical ventilation. *Neurol. India* 59, 707 (2011).

258. Kurnik, D. *et al.* Hyperkalemia and renal function during monotherapy and dual renin-angiotensin blockade in the community setting. *Clin. Ther.* 33, 456–464 (2011).

259. Kwak, J. R., Gwon, M., Lee, J. H., Park, M. S. & Kim, S. H. Non-Oliguric Hyperkalemia in Extremely Low Birth Weight Infants. *Yonsei Med. J.* 54, 696 (2013).

260. Laflamme, E. *et al.* Usefulness of a titration algorithm for de novo users of sacubitril/valsartan in a tertiary centre heart failure clinic. *Cardiovasc. J. Afr.* 29, 352–356 (2018).

261. Lafrance, J.-P. & Miller, D. R. Dispensed selective and nonselective nonsteroidal anti-inflammatory drugs and the risk of moderate to severe hyperkalemia: a nested case-control study. *Am. J. Kidney Dis.* 60, 82–89 (2012).

262. Lawson, D. H., O’Connor, P. C. & Jick, H. Drug attributed alterations in potassium handling in congestive cardiac failure. *Eur. J. Clin. Pharmacol.* 23, 21–25 (1982).

263. Lean, Q., Pham, B., Shamsuddin, N. & Wan Ahmad, W. . Underutilization of angiotensin converting enzyme inhibitors among heart failure patients. *Med. J. Malaysia* 63, 216–221 (2008).

264. Lee, J.-H. *et al.* The effect of renin-angiotensin system blockade on renal protection in chronic kidney disease patients with hyperkalemia. *JRAAS - J. Renin-Angiotensin-Aldosterone Syst.* 15, 491–497 (2014).

265. Lee, K. K. *et al.* Effectiveness and safety of spironolactone for systolic heart failure. *Am. J. Cardiol.* 112, 1427–1432 (2013).

266. Lee, S. *et al**.* Lower serum potassium associated with increased mortality in dialysis patients: A nationwide prospective observational cohort study in Korea. *PLoS One* 12, e0171842 (2017).

267. Legrand, M. *et al.* Association between hypo- and hyperkalemia and outcome in acute heart failure patients: the role of medications. *Clin. Res. Cardiol.* 107, 214–221 (2018).

268. Lemoine, L. *et al.* Incidence of hyperkalemia in the emergency department: a 10-year retrospective study. *Intern. Emerg. Med.* 15, 727–728 (2020).

269. Leslie, G. I., Carman, G. & Arnold, J. D. Early neonatal hyperkalaemia in the extremely premature newborn infant. *J. Paediatr. Child Health* 26, 58–61 (1990).

270. Lewis, J. R., Hassan, S. K. Z., Wenn, R. T. & Moran, C. G. Mortality and serum urea and electrolytes on admission for hip fracture patients. *Injury* 37, 698–704 (2006).

271. Li, Y. *et al.* Electrolyte and acid-base disorders in cancer patients and its impact on clinical outcomes: evidence from a real-world study in China. *Ren. Fail.* 42, 234–243 (2020).

272. Liamis, G. *et al.* Electrolyte disorders in community subjects: Prevalence and risk factors. *Am. J. Med.* 126, 256–263 (2013).

273. Liborio, A. B., Leite, T. T., Neves, F. M. de O., Teles, F. & Bezerra, C. T. de M. AKI complications in critically ill patients: Association with mortality rates and RRT. *Clin. J. Am. Soc. Nephrol.* 10, 21–28 (2015).

274. Lim, C. C. *et al.* Short-Course Systemic and Topical Non-Steroidal Anti-Inflammatory Drugs: Impact on Adverse Renal Events in Older Adults with Co-Morbid Disease. *Drugs and Aging* 38, 147–156 (2021).

275. Lima, M. V. *et al.* Hyperkalemia during spironolactone use in patients with decompensated heart failure. *Arq. Bras. Cardiol.* 91, 177–199 (2008).

276. Lin, C.-C. *et al.* Angiotensin receptor blockers are associated with lower mortality than ACE inhibitors in predialytic stage 5 chronic kidney disease: A nationwide study of therapy with renin-angiotensin system blockade. *PLoS One* 12, e0189126 (2017).

277. Lin, C.-S. *et al.* A Deep-Learning Algorithm (ECG12Net) for Detecting Hypokalemia and Hyperkalemia by Electrocardiography: Algorithm Development. *JMIR Med. Informatics* 8, e15931 (2020).

278. Lin, H.-H. *et al.* Renin-Angiotensin System Blockade Is Not Associated with Hyperkalemia in Chronic Hemodialysis Patients. *Ren. Fail.* 31, 942–945 (2009).

279. Lin, Y.-F. *et al.* Potential target-organ protection of mineralocorticoid receptor antagonist in acute kidney disease. *J. Hypertens.* 37, 125–134 (2019).

280. Linde, C. *et al.* Real-World Associations of Renin-Angiotensin-Aldosterone System Inhibitor Dose, Hyperkalemia, and Adverse Clinical Outcomes in a Cohort of Patients With New-Onset Chronic Kidney Disease or Heart Failure in the United Kingdom. *J. Am. Heart Assoc.* 8, e012655 (2019).

281. Linde, C. *et al.* Serum potassium and clinical outcomes in heart failure patients: results of risk calculations in 21 334 patients in the UK. *ESC Hear. Fail.* 6, 280–290 (2019).

282. Lindner, G., Pfortmuller, C. A., Leichtle, A. B., Fiedler, G. M. & Exadaktylos, A. K. Age-Related Variety in Electrolyte Levels and Prevalence of Dysnatremias and Dyskalemias in Patients Presenting to the Emergency Department. *Gerontology* 60, 420–423 (2014).

283. Ling, L. L. *et al.* Serum potassium and handgrip strength as predictors of sleep quality among hemodialysis patients in Malaysia. *Asia Pac. J. Clin. Nutr.* 28, 401–410 (2019).

284. Lisi, F. *et al.* Mineralcorticoid Receptor Antagonist Withdrawal for Hyperkalemia and Mortality in Patients with Heart Failure. *CardioRenal Med.* 10, 145–153 (2020).

285. Liu, B. *et al.* Utilization of antihypertensive drugs among chronic kidney disease patients: Results from the Chinese cohort study of chronic kidney disease (C-STRIDE). *J. Clin. Hypertens.* 22, 57–64 (2020).

286. Liu, J. *et al.* Association of Sex with Serum Potassium, Sodium, and Calcium Disorders after Hypertensive Intracerebral Hemorrhage. *World Neurosurg.* (2020).

287. Liu, S. *et al.* Association between average plasma potassium levels and 30-day mortality during hospitalization in patients with covid-19 in wuhan, china. *Int. J. Med. Sci.* 18, 736–743 (2021).

288. Liu, Y. *et al.* Serum Potassium Profile and Associated Factors in Incident Peritoneal Dialysis Patients. *Kidney Blood Press. Res.* 41, 545–551 (2016).

289. Livingston, M. H., Singh, S. & Merritt, N. H. Massive transfusion in paediatric and adolescent trauma patients: incidence, patient profile, and outcomes prior to a massive transfusion protocol. *Injury* 45, 1301–1306 (2014).

290. Lopes, R. J., Lourenço, A. P., Mascarenhas, J., Azevedo, A. & Bettencourt, P. Safety of Spironolactone Use in Ambulatory Heart Failure Patients. *Clin. Cardiol.* 31, 509–513 (2008).

291. Lorenz, J. M., Kleinman, L. I. & Markarian, K. Potassium metabolism in extremely low birth weight infants in the first week of life. *J. Pediatr.* 131, 81–86 (1997).

292. Loutradis, C., Tolika, P., Skodra, A., Avdelidou, A. & Sarafidis, P. A. Prevalence of Hyperkalemia in Diabetic and Non-Diabetic Patients with Chronic Kidney Disease: A Nested Case-Control Study. *Am. J. Nephrol.* 42, 351–360 (2015).

293. Lu, X.-H., Su, C., Sun, L. . & Chen, S. Implementing Continuous Quality Improvement Process in Potassium Management in Peritoneal Dialysis Patients. *J. Ren. Nutr.* 19, 469–474 (2009).

294. Luo, J., Brunelli, S. M., Jensen, D. E. & Yang, A. Association between Serum Potassium and Outcomes in Patients with Reduced Kidney Function. *Clin. J. Am. Soc. Nephrol.* 11, 90–100 (2016).

295. Ma, W. *et al.* Serum Potassium Levels and Short-Term Outcomes in Patients With ST-Segment Elevation Myocardial Infarction. *Angiology* 67, 729–736 (2016).

296. Macedo, E., Awdishu, L., Lee, E. & Mehta, R. Course and Outcomes of Hyperkalemia in Hospitalized Patients. in *American Society of Nephrology | Kidney Week - Abstract Details* (2018).

297. Maciejewski, M. L. *et al.* Appropriate baseline laboratory testing following ACEI or ARB initiation by Medicare FFS beneficiaries. *Pharmacoepidemiol. Drug Saf.* 25, 1015–1022 (2016).

298. Maddirala, S. *et al.* Effect of angiotensin converting enzyme inhibitors and angiotensin receptor blockers on serum potassium levels and renal function in ambulatory outpatients: Risk factors analysis. *Am. J. Med. Sci.* 336, 330–335 (2008).

299. Maitland, K. *et al.* Perturbations in Electrolyte Levels in Kenyan Children with Severe Malaria Complicated by Acidosis. *Clin. Infect. Dis.* 40, 9–16 (2005).

300. Maiwall, R. *et al.* AKI in patients with acute on chronic liver failure is different from acute decompensation of cirrhosis. *Hepatol. Int.* 9, 627–639 (2015).

301. Malabu, U. H. *et al.* Prevalence of hyponatremia in acute medical admissions in tropical Asia Pacific Australia. *Asian Pac. J. Trop. Med.* 7, 40–43 (2014).

302. Malvia, S., Goyal, S., Meena, P., Poswal, L. & Meena, M. Renal functions in term neonates admitted with dehydration in a tertiary care centre in southern Rajasthan. *Curr. Pediatr. Res.* 22, 215–218 (2018).

303. Marcussen, M. *et al.* Abnormal serum potassium levels and 6-month all-cause mortality in patients co-treated with antipsychotic and diuretic drugs - A Danish register-based cohort study. *Eur. Neuropsychopharmacol.* 28, 1006–1014 (2018).

304. Marrie, T. & Wu, L. Factors Influencing In-hospital Mortality in Community Acquired Pneumonia. *Chest* 127, (2005).

305. Martens, P. *et al.* The importance of developing hyperkalaemia in heart failure during long-term follow-up. *Acta Cardiol.* 1–9 (2020).

306. Martin-Perez, M., Ruigomez, A., Michel, A. & Garcia Rodriguez, L. A. Impact of hyperkalaemia definition on incidence assessment: implications for epidemiological research based on a large cohort study in newly diagnosed heart failure patients in primary care. *BMC Fam. Pract.* 17, 51 (2016).

307. Masoudi, F. A. *et al.* Adoption of spironolactone therapy for older patients with heart failure and left ventricular systolic dysfunction in the United States, 1998-2001. *Circulation* 112, 39–47 (2005).

308. Mateti, U. V. *et al.* Pattern of Angiotensin-converting enzyme inhibitors induced adverse drug reactions in South Indian teaching hospital. *N. Am. J. Med. Sci.* 4, 185–189 (2012).

309. Mathieson, L., Severn, A. & Guthrie, B. Monitoring and adverse events in relation to ACE inhibitor/angiotensin receptor blocker initiation in people with diabetes in general practice: a population database study. *Scott. Med. J.* 58, 69–76 (2013).

310. Matsushita, K. *et al.* Dyskalemia, its patterns, and prognosis among patients with incident heart failure: A nationwide study of US veterans. *PLoS One* 14, e0219899 (2019).

311. May, H. *et al.* Abstract 10620: The HyperK Score is a Powerful Predictor of Incident Hyperkalemia in a Large General Healthcare Population | Circulation. in *Circulation* (2019).

312. Mazzoni, M. B. *et al.* Hyponatremia in infants with community-acquired infections on hospital admission. *PLoS One* 14, e0219299 (2019).

313. McCauley, J. *et al.* Labetalol-Induced Hyperkalemia in Renal Transplant Recipients. *Am. J. Nephrol.* 22, 347–351 (2002).

314. McDowell, S., Coleman, J., Evans, S., Gill, P. & Ferner, R. Laboratory monitoring and adverse patient outcomes with antihypertensive therapy in primary care. *Pharmacoepidemiol. Drug Saf.* 19, 482–489 (2010).

315. McMahon, G. M., Mendu, M. L., Gibbons, F. K. & Christopher, K. B. Association between hyperkalemia at critical care initiation and mortality. *Intensive Care Med.* 38, 1834–1842 (2012).

316. Medani, S. A., Kheir, A. E. M. & Mohamed, M. B. Acute kidney injury in asphyxiated neonates admitted to a tertiary neonatal unit in Sudan. *Sudan. J. Paediatr.* 14, 29–34 (2014).

317. Mehrotra, S., Sharma, R. K. & Patel, M. R. Vitamin D, 1,25-Dihydroxyvitamin D, FGF23, and graft function after renal transplantation. *Indian J. Nephrol.* 29, 242–247 (2019).

318. Mezzano, G. *et al.* Hyperkalemia influences the outcome of patients with cirrhosis with acute decompensation (AD) and acute-on-chronic liver failure (ACLF). *Dig. Liver Dis.* 53, 738–745 (2021).

319. Michel, A., Martin-Perez, M., Ruigomez, A. & Garcia Rodriguez, L. A. Risk factors for hyperkalaemia in a cohort of patients with newly diagnosed heart failure: a nested case-control study in UK general practice. *Eur. J. Heart Fail.* 17, 205–213 (2015).

320. Milani, G. P. *et al.* Electrolyte and acid-base abnormalities in infants with community-acquired acute pyelonephritis: Prospective cross-sectional study. *Nephron* 137, 99–104 (2017).

321. Molla, M. D. *et al.* Assessment of serum electrolytes and kidney function test for screening of chronic kidney disease among Ethiopian Public Health Institute staff members, Addis Ababa, Ethiopia. *BMC Nephrol.* 21, 1–11 (2020).

322. Moore, C. R., Lin, J. J., O’Connor, N. & Halm, E. A. Follow-up of markedly elevated serum potassium results in the ambulatory setting: implications for patient safety. *Am. J. Med. Qual.* 21, 115–124 (2006).

323. Moore, M. L. & Bailey, R. R. Hyperkalaemia in patients in hospital. *N. Z. Med. J.* 102, 557–558 (1989).

324. Moranne, O. *et al.* Timing of Onset of CKD-Related Metabolic Complications. *J. Am. Soc. Nephrol.* 20, 164–171 (2009).

325. Morris, T. G. *et al.* The potential role of the eGFR in differentiating between true and pseudohyperkalaemia. *Ann. Clin. Biochem. Int. J. Lab. Med.* 57, 444–455 (2020).

326. Mousavi, S.-A. J. *et al.* Comparison of the Serum Electrolyte Levels among Patients Died and Survived in the Intensive Care Unit. *Tanaffos* 11, 36–42 (2012).

327. Movilli, E., Camerini, C., Gaggia, P., Zubani, R. & Cancarini, G. Use of Renin-Angiotensin System Blockers Increases Serum Potassium in Anuric Hemodialysis Patients. *Am. J. Nephrol.* 48, 79–86 (2018).

328. Mu, F. *et al.* Prevalence and economic burden of hyperkalemia in the United States Medicare population. *Curr. Med. Res. Opin.* 36, 1333–1341 (2020).

329. Muhlestein, J. B. *et al.* Frequency and clinical impact of hyperkalaemia within a large, modern, real-world heart failure population. *ESC Hear. Fail.* 8, 691–696 (2021).

330. Mumtaz, M., Raza, M. & Ali, I. Prevalence of hyperkalemia in myocardial infarction. *Pakistan J. Med. Heal. Sci.* 11, 1224–1226 (2017).

331. Nakhoul, G. N. *et al.* Serum Potassium, End-Stage Renal Disease and Mortality in Chronic Kidney Disease. *Am. J. Nephrol.* 41, 456–463 (2015).

332. Nash, D. M. *et al.* Nonsteroidal anti-inflammatory drug use and risk of acute kidney injury and hyperkalemia in older adults: a population-based study. *Nephrol. Dial. Transplant.* 34, 1145–1154 (2019).

333. Nash, D. & Garg, A. X. Risk of AKI and Hyperkalemia Among Older Patients Prescribed Non-Steroidal Anti-Inflammatory Drugs. in *American Society of Nephrology | Kidney Week - Abstract Details* (2018).

334. Navarrete, N. Hyperkalemia in electrical burns: A retrospective study in Colombia. *Burns* 44, 941–946 (2018).

335. Nayeri, A. *et al.* Temporal Pattern and Prognostic Significance of Hypokalemia in Patients Undergoing Targeted Temperature Management Following Cardiac Arrest. *Am. J. Cardiol.* 120, 1110–1113 (2017).

336. Nemati, E. & Taheri, S. Electrocardiographic manifestations of hyperkalemia in hemodialysis patients. *Saudi J. Kidney Dis. Transplant.* 21, 471–477 (2010).

337. Nguyen, A. T., Gentry, C. A. & Furrh, R. Z. A comparison of adverse drug reactions between high- and standard-dose trimethoprim-sulfamethoxazole in the ambulatory setting. *Curr. Drug Saf.* 8, 114–119 (2013).

338. Nilsson, E. *et al.* Incidence and determinants of hyperkalemia and hypokalemia in a large healthcare system. *Int. J. Cardiol.* 245, 277–284 (2017).

339. Nishihara, T. *et al.* Serum Potassium and Cardiovascular Events in Heart Failure With Preserved Left Ventricular Ejection Fraction Patients. *Am. J. Hypertens.* 31, 1098–1105 (2018).

340. Norring-Agerskov, D. *et al.* Hyperkalemia is Associated with Increased 30-Day Mortality in Hip Fracture Patients. *Calcif. Tissue Int.* 101, 9–16 (2017).

341. Nowak, E. *et al.* Safety of fixed dose of antihypertensive drug combinations compared to (single pill) free-combinations a nested matched case-control analysis. *Med. (United States)* 94, e2229 (2015).

342. Nunez, J. *et al.* Long-Term Potassium Monitoring and Dynamics in Heart Failure and Risk of Mortality. *Circulation* 137, 1320–1330 (2018).

343. Ogedegbe, G. *et al.* Comparative Effectiveness of Angiotensin-Converting Enzyme Inhibitor-Based Treatment on Cardiovascular Outcomes in Hypertensive Blacks Versus Whites. *J. Am. Coll. Cardiol.* 66, 1224–1233 (2015).

344. Oh, Y. J. *et al.* The Impact of Renin-Angiotensin System Blockade on Renal Outcomes and Mortality in Pre-Dialysis Patients with Advanced Chronic Kidney Disease. *PLoS One* 12, e0170874 (2017).

345. Okparavero, A. *et al.* Prevalence and complications of chronic kidney disease in a representative elderly population in Iceland. *Nephrol. Dial. Transplant.* 31, 439–447 (2016).

346. Olson, R. P., Schow, A. J., McCann, R., Lubarsky, D. A. & Gan, T. J. Absence of adverse outcomes in hyperkalemic patients undergoing vascular access surgery. *Can. J. Anaesth.* 50, 553–557 (2003).

347. Ookuma, T. *et al.* The clinical relevance of plasma potassium abnormalities on admission in trauma patients: a retrospective observational study. *J. Intensive Care* 3, 37 (2015).

348. Overwyk, K. J. *et al.* Serum Sodium and Potassium Distribution and Characteristics in the US Population, National Health and Nutrition Examination Survey 2009–2016. *J. Appl. Lab. Med.* 6, 63–78 (2021).

349. Paice, B., Gray, J. M., McBride, D., Donnelly, T. & Lawson, D. H. Hyperkalaemia in patients in hospital. *BMJ* 286, 1189–1192 (1983).

350. Palaka, E., Jackson, J., Moon, R. & Grandy, S. HEALTH STATE UTILITY OF CKD PATIENTS WITH HYPERKALEMIA: ANALYSIS OF EQ-5D IN A REAL WORLD POPULATION ACROSS THE EU-5, CHINA AND USA. *Nephrol. Dial. Transplant.* 34, (2019).

351. Pan, X. *et al.* Perioperative complications in liver transplantation using donation after cardiac death grafts: A propensity-matched study. *Liver Transplant.* 20, 823–830 (2014).

352. Panuccio, V. *et al.* EPIDEMIOLOGY OF HYPERKALIEMIA IN CKD: A LONGITUDINAL STUDY. *Nephrol. Dial. Transplant.* 33, i146–i146 (2018).

353. Park, I.-W. *et al.* Onset time of hyperkalaemia after angiotensin receptor blocker initiation: when should we start serum potassium monitoring?. *J. Clin. Pharm. Ther.* 39, 61–68 (2014).

354. Park, I. *et al.* Comparison of hyperkalemic risk in hospitalized patients treated with different angiotensin receptor blockers: A retrospective cohort study using a Korean clinical research database. *Am. J. Cardiovasc. Drugs* 12, 255–262 (2012).

355. Park, K. S. *et al.* Clinical risk factors of postoperative hyperkalemia after adrenalectomy in patients with aldosterone-producing adenoma. *Eur. J. Endocrinol.* 172, 725–731 (2015).

356. Passare, G., Viitanen, M., Torring, O., Winblad, B. & Fastbom, J. Sodium and potassium disturbances in the elderly: Prevalence and association with drug use. *Clin. Drug Investig.* 24, 535–544 (2004).

357. Pathak, J. V & Dass, E. E. A retrospective study of the effects of angiotensin receptor blockers and angiotensin converting enzyme inhibitors in diabetic nephropathy. *Indian J. Pharmacol.* 47, 148–152 (2015).

358. Pecoits‐Filho, R. *et al.* Prescription of renin‐angiotensin‐aldosterone system inhibitors (RAASi) and its determinants in patients with advanced CKD under nephrologist care. *J. Clin. Hypertens.* 21, 991–1001 (2019).

359. Pedersen, K. R. *et al.* Clinical outcome in children with acute renal failure treated with peritoneal dialysis after surgery for congenital heart disease. *Kidney Int.* 73, S81–S86 (2008).

360. Peppin, K. L., Tellor, K. B., Armbruster, A. L. & Schwarze, M. W. Evaluating the safety and tolerability of inpatient sacubitril/valsartan initiation in a community hospital. *J. Community Hosp. Intern. Med. Perspect.* 10, 38–44 (2020).

361. Perkins, R. M., Aboudara, M. C., Abbott, K. C. & Holcomb, J. B. Resuscitative hyperkalemia in noncrush trauma: a prospective, observational study. *Clin. J. Am. Soc. Nephrol.* 2, 313–319 (2007).

362. Peter, S. A. Electrolyte disorders and renal dysfunction in acquired immunodeficiency syndrome patients. *J. Natl. Med. Assoc.* 83, 889–891 (1991).

363. Pfortmüller, C. A., Leichtle, A. B., Fiedler, G. M., Exadaktylos, A. K. & Lindner, G. Hyperkalemia in the emergency department: Etiology, symptoms and outcome of a life threatening electrolyte disorder. *Eur. J. Intern. Med.* 24, e59–e60 (2013).

364. Phillips, B. M. *et al.* Severe hyperkalaemia: demographics and outcome. *Clin. Kidney J.* 7, 127–133 (2014).

365. Pillay, P., Pillay, S. & Mchunu, N. The spectrum of electrolyte abnormalities in black African people living with human immunodeficiency virus and diabetes mellitus at Edendale Hospital, Pietermaritzburg, South Africa. *South. Afr. J. HIV Med.* 21, 1–8 (2020).

366. Pisoni, R. *et al.* Long-term effects of aldosterone blockade in resistant hypertension associated with chronic kidney disease. *J. Hum. Hypertens.* 26, 502–506 (2012).

367. Polcwiartek, C. *et al.* Prognostic role of serum sodium levels across different serum potassium levels in heart failure patients: A Danish register-based cohort study. *Int. J. Cardiol.* 272, 244–249 (2018).

368. Polson, M. *et al.* Clinical and economic impact of hyperkalemia in patients with chronic kidney disease and heart failure. *J. Manag. Care Spec. Pharm.* 23, S2–S20 (2017).

369. Popp, D. & Achtenburg, J. Hyperkalemia and hyperglycemic increments in plasma potassium in diabetes mellitus. *Arch. Intern. Med.* 140, 1617–1621 (1980).

370. Pourfarziani, V. *et al.* Laboratory variables and treatment adequacy in hemodialysis patients in Iran. *Saudi J. Kidney Dis. Transplant.* 19, 842–846 (2008).

371. Pradhan, D., Meher, B., Panda, S. & Samal, D. Prevalence and factors affecting prognosis in neonates with acute kidney injury in a neonatal intensive care unit. *J. Clin. Neonatol.* 7, 237–242 (2018).

372. Prakash, J., Hota, J. K., Singh, S. & Sharma, O. P. Clinical spectrum of chronic renal failure in the elderly: a hospital based study from eastern India. *Int. Urol. Nephrol.* 38, 821–827 (2006).

373. Prasad, G. V. R. *et al.* Safety and efficacy of prophylaxis for Pneumocystis jirovecii pneumonia involving trimethoprim-sulfamethoxazole dose reduction in kidney transplantation. *BMC Infect. Dis.* 19, 311 (2019).

374. Provenzano, M. *et al.* Competing-Risk Analysis of Death and End Stage Kidney Disease by Hyperkalaemia Status in Non-Dialysis Chronic Kidney Disease Patients Receiving Stable Nephrology Care. *J. Clin. Med.* 7, 499 (2018).

375. Pun, P. H., Goldstein, B. A., Gallis, J. A., Middleton, J. P. & Svetkey, L. P. Serum Potassium Levels and Risk of Sudden Cardiac Death Among Patients With Chronic Kidney Disease and Significant Coronary Artery Disease. *Kidney Int. Reports* 2, 1122–1131 (2017).

376. Qiao, Y. *et al.* Association Between Renin-Angiotensin System Blockade Discontinuation and All-Cause Mortality Among Persons With Low Estimated Glomerular Filtration Rate. *JAMA Intern. Med.* 180, 718 (2020).

377. Qiao, Y. *et al.* Discontinuation of Angiotensin Converting Enzyme Inhibitors and Angiotensin Receptor Blockers in Chronic Kidney Disease. *Mayo Clin. Proc.* 94, 2220–2229 (2019).

378. Raebel, M. A. *et al.* Increasingly restrictive definitions of hyperkalemia outcomes in a database study: effect on incidence estimates. *Pharmacoepidemiol. Drug Saf.* 19, 19–25 (2010).

379. Raebel, M. A. *et al.* Diabetes and drug-associated hyperkalemia: Effect of potassium monitoring. *J. Gen. Intern. Med.* 25, 326–333 (2010).

380. Raebel, M. A. *et al.* The positive predictive value of a hyperkalemia diagnosis in automated health care data. *Pharmacoepidemiol. Drug Saf.* 19, 1204–1208 (2010).

381. Rajput, J., Moore, L. S. P., Mughal, N. & Hughes, S. Evaluating the risk of hyperkalaemia and acute kidney injury with cotrimoxazole: a retrospective observational study. *Clin. Microbiol. Infect.* (2020).

382. Ramadan, F. H., Masoodi, N. & El-Solh, A. A. Clinical factors associated with hyperkalemia in patients with congestive heart failure. *J. Clin. Pharm. Ther.* 30, 233–239 (2005).

383. Ramirez, E. *et al.* Drug-induced life-threatening potassium disturbances detected by a pharmacovigilance program from laboratory signals. *Eur. J. Clin. Pharmacol.* 69, 97–110 (2013).

384. Raml, A., Schmekal, B., Grafinger, P. & Biesenbach, G. Risk for hyperkalemia during long-term treatment with angiotensin-converting enzyme inhibitors in insulin-dependent type 2 diabetics in relation to the glomerular filtration rate. *Dtsch. Medizinische Wochenschrift* 126, 1327–1330 (2001).

385. Ramos, C. I. *et al.* Does dietary potassium intake associate with hyperkalemia in patients with chronic kidney disease? *Nephrol. Dial. Transplant.* 1–9 (2020). doi:10.1093/ndt/gfaa232

386. Raphael, K. L., Zhang, Y., Ying, J. & Greene, T. Prevalence of and risk factors for reduced serum bicarbonate in chronic kidney disease. *Nephrology* 19, 648–654 (2014).

387. Ravioli, S., Gygli, R., Funk, G.-C., Exadaktylos, A. & Lindner, G. Prevalence and impact on outcome of sodium and potassium disorders in patients with community-acquired pneumonia: A retrospective analysis. *Eur. J. Intern. Med.* 85, 63–67 (2021).

388. Ravioli, S. *et al.* Dyskalemias in patients with acute kidney injury presenting to the emergency department are common and independent predictors of adverse outcome. *Int. J. Clin. Pract.* 75, 1–6 (2021).

389. Ravn Jacobsen, M. *et al.* Potassium Disturbances and Risk of Ventricular Fibrillation Among Patients With ST‐Segment–Elevation Myocardial Infarction. *J. Am. Heart Assoc.* 9, (2020).

390. Rayan, N., Baird, R. & Masica, A. Rapid response team interventions for severe hyperkalemia: evaluation of a patient safety initiative. *Hosp. Pract.* 39, 161–169 (2011).

391. Raza, S. *et al.* A prospective study on red blood cell transfusion related hyperkalemia in critically ill patients. *J. Clin. Med. Res.* 7, 417–421 (2015).

392. Reardon, L. C. & Macpherson, D. S. Hyperkalemia in Outpatients Using Angiotensin-Converting Enzyme Inhibitors. *Arch. Intern. Med.* 158, 26 (1998).

393. Regolisti, G. *et al.* Electrocardiographic T wave alterations and prediction of hyperkalemia in patients with acute kidney injury. *Intern. Emerg. Med.* 15, 463–472 (2020).

394. Reshma, S. *et al.* Serum electrolytes levels in patients with type 2 diabetes mellitus: a cross-sectional study. *Diabetes Mellit.* 23, 223–228 (2020).

395. Ribeiro, H. S. *et al.* PREVALENCE AND RISK FACTORS OF HYPERKALEMIA AFTER LIVER TRANSPLANTATION. *Arq. Bras. Cir. Dig.* 31, e1357 (2018).

396. Ribeiro, S. C., Figueiredo, A. E., Barretti, P., Pecoits-Filho, R. & De Moraes, T. P. Impact of Renin-Angiotensin Aldosterone System Inhibition on Serum Potassium Levels among Peritoneal Dialysis Patients. *Am. J. Nephrol.* 46, 150–155 (2017).

397. Rimmer, J. M., Horn, J. & Gennari, F. Hyperkalemia as a complication of drug therapy. *Arch. Intern. Med.* 147, 867–869 (1987).

398. Rivera‐Juárez, A. *et al.* Clinical Characteristics and Electrophysiological Mechanisms Underlying Brugada ECG in Patients With Severe Hyperkalemia. *J. Am. Heart Assoc.* 8, e010115 (2019).

399. Ronksley, P. E. *et al.* Potentially preventable hospitalization among patients with CKD and high inpatient use. *Clin. J. Am. Soc. Nephrol.* 11, 2022–2031 (2016).

400. Ronksley, P. E. *et al.* Emergency department use among patients with CKD: A Population-Based Analysis. *Clin. J. Am. Soc. Nephrol.* 12, 304–314 (2017).

401. Ronksley, P. E. *et al.* Derivation and Internal Validation of a Clinical Risk Prediction Tool for Hyperkalemia-Related Emergency Department Encounters Among Hemodialysis Patients. *Can. J. Kidney Heal. Dis.* 7, 205435812095328 (2020).

402. Ross, J. & DeatherageHand, D. Evaluation of potassium levels before hemodialysis access procedures. *Semin. Dial.* 28, 90–93 (2015).

403. Rossignol, P. *et al.* Hyperkalaemia prevalence, recurrence and management in chronic haemodialysis: a prospective multicentre French regional registry 2-year survey. *Nephrol. Dial. Transplant.* 32, 2112–2118 (2017).

404. Ruggenenti, P., Cravedi, P., Chianca, A., Caruso, M. & Remuzzi, G. Achieving remission of proteinuria in childhood CKD. *Pediatr. Nephrol.* 32, 321–330 (2017).

405. Sadeghi-Bojd, S., Noori, S., Damani, E. & Teimouri, A. Electrolyte disturbances in PICU: A cross sectional study. *Nephrourol. Mon.* 11, e87925 (2019).

406. Sadjadi, S. A., McMillan, J. I., Jaipaul, N., Blakely, P. & Hline, S. S. A comparative study of the prevalence of hyperkalemia with the use of angiotensinconverting enzyme inhibitors versus angiotensin receptor blockers. *Ther. Clin. Risk Manag.* 5, 547–552 (2009).

407. Saevik, Å. B. *et al.* Clues for early detection of autoimmune Addison’s disease - myths and realities. *J. Intern. Med.* 283, 190–199 (2018).

408. Safari, S., Hashemi, B., Forouzanfar, M. M., Shahhoseini, M. & Heidari, M. Epidemiology and Outcome of Patients with Acute Kidney Injury in Emergency Department; a Cross-Sectional Study. *Emergency* 6, e30 (2018).

409. Safari, S. *et al.* 20-Day Trend of Serum Potassium Changes in Bam Earthquake Victims with Crush Syndrome; a Cross-sectional Study. *Emerg. (Tehran, Iran)* 5, e5 (2017).

410. Saito, M., Nakayama, D., Takada, M., Hirooka, K. & Yasumura, Y. Carvedilol accelerate elevation of serum potassium in chronic heart failure patients administered spironolactone plus furosemide and either enalapril maleate or candesartan cilexetil. *J. Clin. Pharm. Ther.* 31, 535–540 (2006).

411. Saito, Y., Yamamoto, H., Nakajima, H., Takahashi, O. & Komatsu, Y. Incidence of and risk factors for newly diagnosed hyperkalemia after hospital discharge in non-dialysis-dependent CKD patients treated with RAS inhibitors. *PLoS One* 12, e0184402 (2017).

412. Sánchez, J. L. C. *et al.* HYPERKALAEMIA AND HAEMODIALYSIS PATIENTS: ELECTROCARDIOGRAPHIC CHANGES. *J. Ren. Care* 33, 124–129 (2007).

413. Santos, R. P. Dos, Carvalho, A. R. da S., Peres, L. A. B., Delfino, V. D. A. & Grion, C. M. C. Non-recovery of renal function is a strong independent risk factor associated with mortality in AKI patients. *Brazilian J. Nephrol.* 42, 290–299 (2020).

414. Sarafidis, P. A. *et al.* Prevalence and factors associated with hyperkalemia in predialysis patients followed in a low-clearance clinic. *Clin. J. Am. Soc. Nephrol.* 7, 1234–1241 (2012).

415. Sarvazad, H., Cahngaripour, S. H., Eskandari Roozbahani, N. & Izadi, B. Evaluation of electrolyte status of sodium, potassium and magnesium, and fasting blood sugar at the initial admission of individuals with COVID-19 without underlying disease in Golestan Hospital, Kermanshah. *New Microbes New Infect.* 38, 100807 (2020).

416. Savarese, G. *et al.* Factors associated with underuse of mineralocorticoid receptor antagonists in heart failure with reduced ejection fraction: an analysis of 11 215 patients from the Swedish Heart Failure Registry. *Eur. J. Heart Fail.* 20, 1326–1334 (2018).

417. Savarese, G. *et al.* Incidence, Predictors, and Outcome Associations of Dyskalemia in Heart Failure With Preserved, Mid-Range, and Reduced Ejection Fraction. *JACC Hear. Fail.* 7, 65–76 (2019).

418. Saxena, A. & Meshram, S. V. Predictors of Mortality in Acute Kidney Injury Patients Admitted to Medicine Intensive Care Unit in a Rural Tertiary Care Hospital. *Indian J. Crit. Care Med.* 22, 231–237 (2018).

419. Schroeder, E. B. *et al.* Predictors of Hyperkalemia and Hypokalemia in Individuals with Diabetes: a Classification and Regression Tree Analysis. *J. Gen. Intern. Med.* (2020).

420. Schwartz, D., Connelly, N. R., Manikantan, P. & Nichols, J. H. Hyperkalemia and Pyloric Stenosis. *Anesth. Analg.* 97, 355–357 (2003).

421. Secora, A. M. *et al.* Hyperkalemia and Acute Kidney Injury with Spironolactone Use Among Patients with Heart Failure. *Mayo Clin. Proc.* 95, 2408–2419 (2020).

422. Semama, D. & Martin-Delgado, M. Metabolism of potassium in preterm infants. *Arch. Pediatr.* 14, 249–253 (2007).

423. Sever, M. S. *et al.* Serum potassium in the crush syndrome victims of the Marmara disaster. *Clin. Nephrol.* 59, 326–333 (2003).

424. Shaffer, S. G., Kilbride, H. W., Hayen, L. K., Meade, V. M. & Warady, B. A. Hyperkalemia in very low birth weight infants. *J. Pediatr.* 121, 275–279 (1992).

425. Shah, G. S., Das, B. K., Kumar, S., Singh, M. K. & Bhandari, G. P. Acid base and electrolyte disturbance in diarrhoea. *Kathmandu Univ. Med. J. (KUMJ)* 5, 60–62 (2007).

426. Shah, K. B., Rao, K., Sawyer, R. & Gottlieb, S. S. The adequacy of laboratory monitoring in patients treated with spironolactone for congestive heart failure. *J. Am. Coll. Cardiol.* 46, 845–849 (2005).

427. Shanmugapriya, S., Bhuvaneswari, K. & Rashmi, K. A study on the effect of low molecular weight on potassium homeostasis in patients administered heparin for thromboprophylaxis in a tertiary care hospital. *Int. J. Pharm. Pharm. Sci.* 9, 85–89 (2017).

428. Shariq, O. A. *et al.* Contralateral suppression of aldosterone at adrenal venous sampling predicts hyperkalemia following adrenalectomy for primary aldosteronism. *Surg. (United States)* 163, 183–190 (2018).

429. Sharma, A., Alvarez, P. J., Woods, S. D. & Dai, D. A model to predict risk of hyperkalemia in patients with chronic kidney disease using a large administrative claims database. *Clin. Outcomes Res.* 12, 657–667 (2020).

430. Sheen, S. S. *et al.* The Model for End-stage Liver Disease score is potentially a useful predictor of hyperkalemia occurrence among hospitalized angiotensin receptor blocker users. *J. Clin. Pharm. Ther.* 40, 48–54 (2015).

431. Shemer, J., Modan, M., Ezra, D. & Cabili, S. Incidence of hyperkalemia in hospitalized patients. *Isr. J. Med. Sci.* 19, 659–661 (1983).

432. Shi, L.-T., Cao, X.-Y., Gu, R., Zhao, J.-X. & Zhang, Y. Incidence and clinical significance of abnormalities in potassium, sodium and calcium levels in elderly patients with hip fractures during the perioperative period. *Ann. Ital. Chir.* 91, 187–191 (2020).

433. Shida, H. *et al.* Serum potassium level on hospital arrival and survival after out-of-hospital cardiac arrest: The CRITICAL study in Osaka, Japan. *Eur. Hear. Journal. Acute Cardiovasc. Care* 9, S175–S183 (2020).

434. Shih, C.-J. *et al.* Angiotensin-converting enzyme inhibitors, angiotensin II receptor blockers and the risk of major adverse cardiac events in patients with diabetes and prior stroke: a nationwide study. *J. Hypertens.* 34, 567–575 (2016).

435. Shih, C.-J. *et al.* Comparative effectiveness of angiotensin-converting-enzyme inhibitors and angiotensin II receptor blockers in patients with type 2 diabetes and retinopathy. *Can. Med. Assoc. J.* 188, E148–E157 (2016).

436. Shih, C.-J., Chu, H., Ou, S.-M. & Chen, Y.-T. Comparative effectiveness of angiotensin-converting enzyme inhibitors and angiotensin II receptor blockers on major adverse cardiac events in patients with newly diagnosed type 2 diabetes: A nationwide study. *Int. J. Cardiol.* 199, 283–289 (2015).

437. Shin, J.-I., Palta, M., Djamali, A., Kaufman, D. B. & Astor, B. C. The Association Between Renin-Angiotensin System Blockade and Long-term Outcomes in Renal Transplant Recipients. *Transplantation* 100, 1541–1549 (2016).

438. Shiyovich, A., Gilutz, H. & Plakht, Y. Serum electrolyte/metabolite abnormalities among patients with acute myocardial infarction: comparison between patients with and without diabetes mellitus. *Postgrad. Med.* 133, 395–403 (2021).

439. Si, J. *et al.* Efficacy and safety of early initiation of sacubitril-valsartan therapy in patients with acute decompensated heart failure. *Chinese J. Cardiol.* 48, 477–483 (2020).

440. Silvarino, R. *et al.* Is Chronic Kidney Disease Progression Influenced by the Type of Renin-Angiotensin-System Blocker Used?. *Nephron* 143, 100–107 (2019).

441. Singer, A. J., Thode Jr, H. C. & Peacock, W. F. A retrospective study of emergency department potassium disturbances: severity, treatment, and outcomes. *Clin. Exp. Emerg. Med.* 4, 73–79 (2017).

442. Singer, A. J., Thode, H. C. & Peacock, W. F. Rapid correction of hyperkalemia is associated with reduced mortality in ED patients. *Am. J. Emerg. Med.* 38, 2361–2364 (2020).

443. Singh, S. *et al.* Impact of blood pressure and serum electrolytes level on ejection fraction in acute myocardial infarction patients. *Natl. J. Physiol. Pharm. Pharmacol.* 10, 1 (2020).

444. Singhi, S. & Dhawan, A. Frequency and significance of electrolyte abnormalities in pneumonia. *Indian Pediatr.* 29, 735–740 (1992).

445. Slob, E. M. A., Shulman, R. & Singer, M. Experience using high-dose glucose-insulin-potassium (GIK) in critically ill patients. *J. Crit. Care* 41, 72–77 (2017).

446. Smith, N. *et al.* Serum potassium and stroke risk among treated hypertensive adults. *Am. J. Hypertens.* 16, 806–813 (2003).

447. Sofue, T. *et al.* Prevalences of hyperuricemia and electrolyte abnormalities in patients with chronic kidney disease in Japan: A nationwide, cross-sectional cohort study using data from the Japan Chronic Kidney Disease Database (J-CKD-DB). *PLoS One* 15, e0240402 (2020).

448. Soleimani, A., Foroozanfard, F. & Tamadon, M. R. Evaluation of water and electrolytes disorders in severe acute diarrhea patients treated by WHO protocol in eight large hospitals in Tehran; a nephrology viewpoint. *J. Ren. Inj. Prev.* 6, 109–112 (2017).

449. Soman, S. *et al.* Limitations in using angiotensin-converting enzyme inhibitors and angiotensin II receptor blockers in the management of heart failure due to comorbidities: An Indian scenario. *Ann. Clin. Cardiol.* 2, 24 (2020).

450. Song, X., Farmer, D. G. & Xia, V. W. Intraoperative Management and Postoperative Outcome in Intestine-Inclusive Liver Transplantation Versus Liver Transplantation. *Transplant. Proc.* 47, 2473–2477 (2015).

451. Song, Y.-H. *et al.* Can we predict who will develop postoperative hyperkalaemia after parathyroidectomy in dialysis patients with secondary hyperparathyroidism? *BMC Nephrol.* 20, 225 (2019).

452. Soule, S. Addison’s disease in Africa-a teaching hospital experience. *Clin. Endocrinol. (Oxf).* 50, 115–120 (1999).

453. Sowunmi, A. Clinical study of cerebral malaria in African children. *Afr. J. Med. Med. Sci.* 26, 9–11 (1997).

454. Spino, M., Sellers, E. M., Kaplan, H. L., Stapleton, C. & MacLeod, S. M. Adverse biochemical and clinical consequences of furosemide administration. *Can. Med. Assoc. J.* 118, 1513–8 (1978).

455. Staatz, C. E., Taylor, P. J., Lynch, S. V & Tett, S. E. A pharmacodynamic investigation of tacrolimus in pediatric liver transplantation. *Liver Transplant.* 10, 506–512 (2004).

456. Sternthal, M. B. *et al.* Adverse Events Associated With the Use of Cyclosporine in Patients With Inflammatory Bowel Disease. *Am. J. Gastroenterol.* 103, 937–943 (2008).

457. Stevens, M. S. & Dunlay, R. W. *Hyperkalemia in hospitalized patients*. *International Urology and Nephrology* 32, (2000).

458. Stewart, I. J. *et al.* Hyperkalemia in Combat Casualties: Implications for Delayed Evacuation. *Mil. Med.* 182, e2046–e2051 (2017).

459. Stewart, R., Marks, R. & Hale, W. Epidemiology of serum potassium levels in an ambulatory elderly population: Effect of age, sex, diuretics, and estimated creatinine clearance. *Drug Intell. Clin. Pharm.* 15, 467–469 (1981).

460. Strozecki, P. *et al.* The evaluation of renin-angiotensin-aldosterone system blocking drugs use in hemodialysed patients. *Nadcisnienie Tetnicze* 11, 310–317 (2007).

461. Su, H. *et al.* Detection and awareness of chronic renal insufficiency and related complications at a tertiary care hospital in China. *Int. J. Clin. Exp. Med.* 10, 12764–12772 (2017).

462. Sui, Z. *et al.* Aetiology of chronic kidney disease and risk factors for disease progression in Chinese subjects: A single-centre retrospective study in Beijing. *Nephrology* (2020).

463. Surabenjawong, U., Thunpiphat, N., Chatsiricharoenkul, S. & Monsomboon, A. Prevalence of hyperkalemia in adult patients taking spironolactone and angiotensin converting enzyme inhibitors or angiotensin receptor blockers. *J. Med. Assoc. Thail.* 96, 905–910 (2013).

464. Svensson, M., Gustafsson, F., Galatius, S., Hildebrandt, P. R. & Atar, D. Hyperkalaemia and impaired renal function in patients taking spironolactone for congestive heart failure: Retrospective study. *BMJ* 327, 1141–1142 (2003).

465. Svensson, M., Gustafsson, F., Galatius, Sø., Hildebrandt, P. R. & Atar, D. How prevalent is hyperkalemia and renal dysfunction during treatment with spironolactone in patients with congestive heart failure? *J. Card. Fail.* 10, 297–303 (2004).

466. Sylvanus, E. *et al.* Profile and outcome of patients with emergency complications of renal failure presenting to an urban emergency department of a tertiary hospital in Tanzania. *BMC Emerg. Med.* 19, 11 (2019).

467. Taber, D. J. *et al.* Are thiazide diuretics safe and effective antihypertensive therapy in kidney transplant recipients? *Am. J. Nephrol.* 38, 285–291 (2013).

468. Tafesse, E. *et al.* Risk factors associated with the incidence and recurrence of hyperkalaemia in patients with cardiorenal conditions. *Int. J. Clin. Pract.* 75, 1–14 (2021).

469. Takahashi, K. Two-year follow-up study of the efficacy and safety of FK 506 in kidney transplant patients. *Transpl. Int.* 7, 247–251 (1994).

470. Takahashi, N., Hoshi, J. & Nishida, H. Water balance, electrolytes and acid-base balance in extremely premature infants. *Acta Paediatr. Jpn.  Overseas Ed.* 36, 250–255 (1994).

471. Takahashi, S. *et al.* Safety and Antihypertensive Effect of Selara (Eplerenone): Results from a Postmarketing Surveillance in Japan. *Int. J. Hypertens.* 2016, 5091951 (2016).

472. Takaichi, K., Takemoto, F., Ubara, Y. & Mori, Y. Analysis of factors causing hyperkalemia. *Intern. Med.* 46, 823–829 (2007).

473. Tamirisa, K. P., Aaronson, K. D. & Koelling, T. M. Spironolactone-induced renal insufficiency and hyperkalemia in patients with heart failure. *Am. Heart J.* 148, 971–978 (2004).

474. Tanaka, K. *et al.* Association between serum potassium levels and adverse outcomes in chronic kidney disease: the Fukushima CKD cohort study. *Clin. Exp. Nephrol.* 25, 410–417 (2021).

475. Terano, C. *et al.* Incidence of and risk factors for severe acute kidney injury in children with heart failure treated with renin-angiotensin system inhibitors. *Eur. J. Pediatr.* 175, 631–637 (2016).

476. Thakur, J. *et al.* Prevalence of electrolyte disturbances in perinatal asphyxia: a prospective study. *Ital. J. Pediatr.* 44, 56 (2018).

477. Thayyil, S., Kempley, S. T. & Sinha, A. Can early-onset nonoliguric hyperkalemia be predicted in extremely premature infants?. *Am. J. Perinatol.* 25, 129–133 (2008).

478. Thiede, R. M. *et al.* Hyperkalemia in women with acne exposed to oral spironolactone: A retrospective study from the RADAR (Research on Adverse Drug Events and Reports) program. *Int. J. Women’s Dermatology* 5, 155–157 (2019).

479. Thomas, A. *et al.* The Frequency of Routine Blood Sampling and Patient Outcomes Among Maintenance Hemodialysis Recipients. *Am. J. Kidney Dis.* 75, 471–479 (2020).

480. Thomsen, R. W. *et al.* Hyperkalaemia in people with diabetes: occurrence, risk factors and outcomes in a Danish population‐based cohort study. *Diabet. Med.* 35, 1051–1060 (2018).

481. Thomsen, R. W. *et al.* Elevated potassium levels in patients with chronic kidney disease: occurrence, risk factors and clinical outcomes—a Danish population-based cohort study. *Nephrol. Dial. Transplant.* 33, 1610–1620 (2018).

482. Thomsen, R. W. *et al.* Elevated Potassium Levels in Patients With Congestive Heart Failure: Occurrence, Risk Factors, and Clinical Outcomes. *J. Am. Heart Assoc.* 7, (2018).

483. Thongprayoon, C. *et al.* Admission Serum Potassium Levels in Hospitalized Patients and One-Year Mortality. *Med. (Basel, Switzerland)* 7, (2019).

484. Thongprayoon, C. *et al.* Serum Potassium Levels at Hospital Discharge and One-Year Mortality among Hospitalized Patients. *Medicina (B. Aires).* 56, (2020).

485. Tiamkao, S., Pranboon, S., Thepsuthammarat, K. & Sawanyawisuth, K. Status epilepticus in the elderly patients: A national data study in Thailand. *J. Neurol. Sci.* 372, 501–505 (2017).

486. Timerga, A. *et al.* Serum electrolytes disorder and its associated factors among adults admitted with metabolic syndrome in Jimma Medical Center, South West Ethiopia: Facility based crossectional study. *PLoS One* 15, e0241486 (2020).

487. Torle, K., Kalantar-Zadeh, K., Molnar, M., Vashistha, T. & Mehrotra, R. Serum potassium and cause-specific mortality in a large peritoneal dialysis cohort. *Clin. J. Am. Soc. Nephrol.* 7, 1272–1284 (2012).

488. Torres, O. H. *et al.* Effect of prophylactic treatment with low-molecular-weight heparin bemiparin sodium on serum potassium levels: a prospective observational study. *Drugs & Ageing* 27, 399–406 (2010).

489. Trakulsrichai, S. *et al.* Clinical characteristics of zinc phosphide poisoning in Thailand. *Ther. Clin. Risk Manag.* Volume 13, 335–340 (2017).

490. Trevisan, M. *et al.* Incidence, predictors and clinical management of hyperkalaemia in new users of mineralocorticoid receptor antagonists. *Eur. J. Heart Fail.* 20, 1217–1226 (2018).

491. Tseng, W.-C. *et al.* Effect of spironolactone on the risks of mortality and hospitalization for heart failure in pre-dialysis advanced chronic kidney disease: A nationwide population-based study. *Int. J. Cardiol.* 238, 72–78 (2017).

492. Uehara, K. *et al.* Incidence and risk factors of postoperative emergency treatment in dialysis patients after elective noncardiac surgery: a five-year retrospective study. *Masui.* 63, 309–314 (2014).

493. Ueno, H. *et al.* Clinical factors affecting serum potassium concentration in cardio-renal decompensation syndrome. *Int. J. Cardiol.* 138, 174–181 (2010).

494. Uijtendaal, E. V, Zwart-van Rijkom, J. E. F., van Solinge, W. W. & Egberts, T. C. G. Frequency of laboratory measurement and hyperkalaemia in hospitalised patients using serum potassium concentration increasing drugs. *Eur. J. Clin. Pharmacol.* 67, 933–940 (2011).

495. Ullah, I. & Khan, I. To determine the frequency and pattern of common electrolyte abnormalities in children presenting with acute gastroenteritis. *Med. Forum Mon.* 31, (2020).

496. Ullian, M., Elliott, A. & Soliman, K. Hyperkalemia in Chronic Peritoneal Dialysis. in *American Society of Nephrology | Kidney Week - Abstract Details* (2019).

497. Uriel, M. *et al.* Incidence and Clinical Significance of Hyperkalemia Following Heart Transplantation. *Transplant. Proc.* 53, 673–680 (2021).

498. Urshila, S., Mergenhagen, K. A. & Kellick, K. Effectiveness and safety of dual renin-angiotensin system blockade: a comparison between younger and older cohorts. *Consult. Pharm.* 28, 383–389 (2013).

499. Utiger, F. *et al.* Hyperkalemia caused by potassium sparing and potassium losing diuretics. *Schweiz. Med. Wochenschr.* 120, 1933–1936 (1990).

500. Velavan, P. *et al.* Predictors of short term mortality in heart failure - insights from the Euro Heart Failure survey. *Int. J. Cardiol.* 138, 63–69 (2010).

501. Vereijken, T. L. J. *et al.* Risk calculation for hyperkalaemia in heart failure patients. *Neth. J. Med.* 65, 208–211 (2007).

502. Vikrant, S. & Parashar, A. Acute kidney injury due to multiple hymenoptera stings-a clinicopathological study. *Clin. Kidney J.* 10, 532–538 (2017).

503. Vikrant, S., Jaryal, A., Gupta, D. & Parashar, A. Epidemiology and outcome of acute kidney injury due to venomous animals from a subtropical region of India. *Clin. Toxicol.* 57, 240–245 (2019).

504. Volterrani, M. *et al.* Effects of hyperkalaemia and non‐adherence to renin–angiotensin–aldosterone system inhibitor therapy in patients with heart failure in <scp>Italy</scp> : a propensity‐matched study. *Eur. J. Heart Fail.* 22, 2049–2055 (2020).

505. von Lilien, T., Salusky, I. B., Boechat, I., Ettenger, R. B. & Fine, R. N. Five year’s experience with continuous ambulatory or continuous cycling peritoneal dialysis in children. *J. Pediatr.* 111, 513–518 (1987).

506. Wada, M., Kusuda, S., Takahashi, N. & Nishida, H. Fluid and electrolyte balance in extremely preterm infants <24 weeks of gestation in the first week of life. *Pediatr. Int.* 50, 331–336 (2008).

507. Wada, N. *et al.* Hyperkalemia in both surgically and medically treated patients with primary aldosteronism. *J. Hum. Hypertens.* 31, 627–632 (2017).

508. Wagner, S. *et al.* Association of plasma potassium with mortality and end-stage kidney disease in patients with chronic kidney disease under nephrologist care - The NephroTest study. *BMC Nephrol.* 18, 295 (2017).

509. Wahr, J. *et al.* Preoperative serum potassium levels and perioperative outcomes in cardiac surgery patients. *J. Am. Med. Assoc.* 281, 2203–2210 (1999).

510. Wallerstedt, S. *et al.* Moderate hyperkalemia in hospitalized patients with cirrhotic ascites indicates a poor prognosis. *Scand. J. Gastroenterol.* 48, 358–365 (2013).

511. Wang, C.-H. *et al.* The effects of calcium and sodium bicarbonate on severe hyperkalaemia during cardiopulmonary resuscitation: A retrospective cohort study of adult in-hospital cardiac arrest. *Resuscitation* 98, 105–111 (2016).

512. Wang, H.-H. *et al.* Hypokalemia, Its Contributing Factors and Renal Outcomes in Patients with Chronic Kidney Disease. *PLoS One* 8, e67140 (2013).

513. Wang, T. *et al.* Effectiveness and safety of aldosterone antagonist therapy use among older patients with reduced ejection fraction after acute myocardial infarction. *J. Am. Heart Assoc.* 5, e002612 (2016).

514. Wannamethee, S. G., Lever, A. F., Shaper, A. G. & Whincup, P. H. Serum potassium, cigarette smoking, and mortality in middle-aged men. *Am. J. Epidemiol.* 145, 598–606 (1997).

515. Wei, L., Struthers, A. D., Fahey, T., Watson, A. D. & Macdonald, T. M. Spironolactone use and renal toxicity: Population based longitudinal analysis. *BMJ* 340, 1233 (2010).

516. Weinberg, L. *et al.* Potassium levels after liver reperfusion in adult patients undergoing cadaveric liver transplantation: A retrospective cohort study. *Ann. Med. Surg.* 55, 111–118 (2020).

517. Wetmore, J. B., Yan, H., Horne, L., Peng, Y. & Gilbertson, D. T. Risk of hyperkalemia from renin–angiotensin–aldosterone system inhibitors and factors associated with treatment discontinuities in a real-world population. *Nephrol. Dial. Transplant.* 36, 826–839 (2021).

518. Wiebe, N. *et al.* Potentially preventable hospitalization as a complication of CKD: A cohort study. *Am. J. Kidney Dis.* 64, 230–238 (2014).

519. Williams, E. M., Katholi, R. E. & Karambelas, M. R. Use and side-effect profile of spironolactone in a private cardiologist’s practice. *Clin. Cardiol.* 29, 149–153 (2006).

520. Wilson, R. F. *et al.* Electrolyte and acid-base changes with massive blood transfusions. *Am. Surg.* 58, 535 (1992).

521. Witham, M. D., Gillespie, N. D. & Struthers, A. Tolerability of spironolactone in patients with chronic heart failure - A cautionary message. *Br. J. Clin. Pharmacol.* 58, 554–557 (2004).

522. Woo, K.-T. *et al.* A retrospective Aliskiren and Losartan study in non-diabetic chronic kidney disease. *World J. Nephrol.* 2, 129–135 (2013).

523. Wu, H.-Y. *et al.* Comparative effectiveness of angiotensin-converting enzyme inhibitors versus angiotensin II receptor blockers for major renal outcomes in patients with diabetes: A 15-year cohort study. *PLoS One* 12, e0177654 (2017).

524. Wu, J.-L. *et al.* Effects of long-term low-dose spironolactone treatment in patients with New York heart association functional class ii heart failure: A 10-year prospective study. *Int. J. Clin. Exp. Med.* 9, 15689–15698 (2016).

525. Wu, Y., Hou, B., Liu, J., Chen, Y. & Zhong, P. Risk Factors Associated With Long-Term Hospitalization in Patients With COVID-19: A Single-Centered, Retrospective Study. *Front. Med.* 7, 315 (2020).

526. Xu, H. *et al.* Dyskalemias and adverse events associated with discharge potassium in acute myocardial infarction. *Am. Heart J.* 205, 53–62 (2018).

527. Yada, Y. *et al.* Synergic interaction between ritodrine and magnesium sulfate on the occurrence of critical neonatal hyperkalemia: A Japanese nationwide retrospective cohort study. *Sci. Rep.* 10, 7804 (2020).

528. Yamada, M. *et al.* Incidence and Outcomes of Hyperkalemia in Solid Organ Transplant Recipients. in *American Society of Nephrology | Kidney Week - Abstract Details* (2018).

529. Yang, C.-T., Kor, C.-T. & Hsieh, Y.-P. Long-Term Effects of Spironolactone on Kidney Function and Hyperkalemia-Associated Hospitalization in Patients with Chronic Kidney Disease. *J. Clin. Med.* 7, 459 (2018).

530. Yang, G. *et al.* Perioperative hyperkalemia in hemodialysis patients undergoing parathyroidectomy for renal hyperparathyroidism. *Intern. Emerg. Med.* 14, 1065–1071 (2019).

531. Yao, D., Wang, L., Curran, S. & Ball, P. Adherence to treatment guidelines in the pharmacological management of chronic heart failure in an Australian population. *J. Geriatr. Cardiol.* 8, 88–92 (2011).

532. Yaseen, H. Nonoliguric Hyperkalemia in Neonates: A Case-Controlled Study. *Am. J. Perinatol.* 26, 185–189 (2009).

533. Yildirim, T. *et al.* Major Barriers against Renin–Angiotensin–Aldosterone System Blocker Use in Chronic Kidney Disease Stages 3–5 in Clinical Practice: A Safety Concern? *Ren. Fail.* 34, 1095–1099 (2012).

534. Younis, A. A. *et al.* Elevated Admission Potassium Levels and 1-Year and 10-Year Mortality Among Patients With Heart Failure. *Am. J. Med. Sci.* 354, 268–277 (2017).

535. Yuan, H., Jeng, M., Soong, W. & Chen, S. Hyperkalemia during the Early Postnatal Days in Premature Infants. *Acta Paediatr. Taiwanica* 44, 208–214 (2003).

536. Yue, Y. *et al.* Effects of long-term low-dose spironolactone in patients with NYHA class II heart failure. *Exp. Clin. Cardiol.* 20, 1747–1768 (2014).

537. Yusuf, A. A., Hu, Y., Singh, B., Menoyo, J. A. & Wetmore, J. B. Serum Potassium Levels and Mortality in Hemodialysis Patients: A Retrospective Cohort Study. *Am. J. Nephrol.* 44, 179–186 (2016).

538. Zaidan, Z. & Abdulhafedh, I. The electrocardiographic abnormalities in patients with end-stage chronic kidney disease in Kirkuk general hospital. *Indian J. Forensic Med. Toxicol.* 13, 486–492 (2019).

539. Zeman, K. *et al.* Short-term prognosis and treatment of patients hospitalized for acute heart failure in a regional hospital without a cardiocentre. *Vnitr. Lek.* 58, 273–279 (2012).

540. Zhang, W. *et al.* Clinical characteristics and outcomes of rural patients with ESRD in Guangxi, China: One dialysis center experience. *Int. Urol. Nephrol.* 42, 195–204 (2010).

541. Zhao, J., Gao, H.-Y., Feng, Z.-Y. & Wu, Q.-J. A Retrospective Analysis of the Clinical and Epidemiological Characteristics of COVID-19 Patients in Henan Provincial People’s Hospital, Zhengzhou, China. *Front. Med.* 7, 286 (2020).

542. Zhou, D. *et al.* Trends in aetiology, treatment and complications associated with diabetic ketoacidosis (DKA) – a 6-year study at a large tertiary care centre in the West Midlands, United Kingdom. *Clin. Med. (Northfield. Il).* 20, s68–s69 (2020).
